# Discovery of Novel Pleuromutilin Derivatives as Potent Antibacterial Agents for the Treatment of MRSA Infection

**DOI:** 10.3390/molecules27030931

**Published:** 2022-01-29

**Authors:** Han-Qing Fang, Jie Zeng, Shou-Kai Wang, Xiao Wang, Fang Chen, Bo Li, Jie Liu, Zhen Jin, Ya-Hong Liu, You-Zhi Tang

**Affiliations:** 1 Guangdong Provincial Key Laboratory of Veterinary Pharmaceutics Development and Safety Evaluation, College of Veterinary Medicine, South China Agricultural University, No. 483 Wushan Road, Tianhe District, Guangzhou 510642, China; fhq1994@126.com (H.-Q.F.); zfz47349@163.com (J.Z.); 18855033867@163.com (S.-K.W.); wangxiao1688@stu.scau.edu.cn (X.W.); fox280259306@163.com (F.C.); libo447051530@163.com (B.L.); clarklj517@gmail.com (J.L.); jinzhenhami@scau.edu.cn (Z.J.); gale@scau.edu.cn (Y.-H.L.); 2 Guangdong Laboratory for Lingnan Modern Agriculture, Guangzhou 510642, China

**Keywords:** antibacterial activity, MRSA, pleuromutilin, SPR, 50S ribosome

## Abstract

A series of novel pleuromutilin derivatives containing nitrogen groups on the side chain of C14 were synthesized under mild conditions. Most of the synthesized derivatives displayed potent antibacterial activities. Compound **9** was found to be the most active antibacterial derivative against MRSA (MIC = 0.06 μg/mL). Furthermore, the result of time-kill curves showed that compound **9** had a certain inhibitory effect against MRSA in vitro. Moreover, according to a surface plasmon resonance (SPR) study, compound **9** (K_D_ = 1.77 × 10^−8^ M) showed stronger affinity to the 50S ribosome than tiamulin (K_D_ = 2.50 × 10^−8^ M). The antibacterial activity of compound **9** was further evaluated in an MRSA-infected murine thigh model. Compared to the negative control group, tiamulin reduced MRSA load (~0.7 log_10_ CFU/mL), and compound **9** performed a treatment effect (~1.3 log_10_ CFU/mL). In addition, compound **9** was evaluated in CYP450 inhibition assay and showed only moderate in vitro CYP3A4 inhibition (IC_50_ = 2.92 μg/mL).

## 1. Introduction

*Staphylococcus aureus* caused a wide range of diseases and had capacities to adapt to diverse environmental forms [1]. Methicillin-resistant *S. aureus* (MRSA) was first reported by Jevons in 1961, and it was the major cause of variety of infections [2]. It has been reported by the Centers for Disease Control and Prevention (CDC) that MRSA induced nearly 120,000 cases of bloodstream infections, causing approximately 20,000 deaths in the year 2017 [3]. In clinical practice, severe infections caused by MRSA were usually treated with vancomycin and daptomycin. However, the permeability-mediated resistances to MRSA of vancomycin and daptomycin were also reported [4]. The prevalence of MRSA has posed a serious threat to global public health security, and there is an urgent need to develop new effective therapeutic agents for it [5].

**Figure 1:**
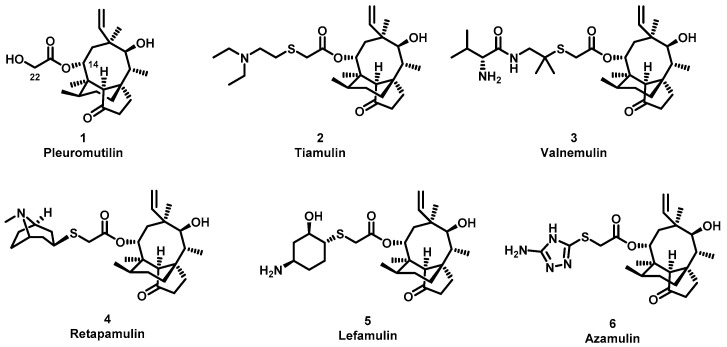
Structure of pleuromutilin (**1**), tiamulin (**2**), valnemulin (**3**), retapamulin (**4**), lefamulin (**5**) and azamulin (**6**).

The (+)-pleuromutilin (**1**, Figure 1), a tricyclic diterpenoid natural product, was first isolated in 1951 [6,7]. Pleuromutilin could inhibit bacterial protein synthesis by binding to the peptidyl transferase center located in the 50S ribosomal subunit [8]. For the unique mechanism, pleuromutilin rarely exhibits cross-resistance with other antibiotics. A large number of semisynthetic pleuromutilin derivatives have been synthesized [9]. Four of them have reached the market, including two veterinary agents, tiamulin [10,11] (**2**, **1975**) and valnemulin [8] (**3**, **1998**), and two agents for human use, retapamulin [12] (**4**, **2007**) and lefamulin [13] (**5**, **2019**). Retapamulin was approved by the US for short-term treatment of skin and soft tissue infections (SSTIs) [14]. Lefamulin was approved by the US for the treatment of community-acquired pneumonia by oral and intravenous administration [15]. In addition, several pleuromutilin derivatives have entered into clinical trials, such as azamulin (**6**, Figure 1). Unfortunately, azamulin was discontinued after Phase I trials due to significant inhibition of human CYP3A4 [16]. It was suggested that the azole-containing structure of azamulin might be one of the reasons for its strong inhibitory effect on CYP3A4 [17]. Thus, we focused our attention on the semisynthetic pleuromutilin derivatives without the azole group.

In our previous work, several series of pleuromutilin derivatives through an amine at the C-22 position showed good antibacterial activity [18,19]. Ultimately, 23 pleuromutilin derivatives, where the thioether was replaced by an amine group at the C22 position, were designed, synthesized and evaluated for their antibacterial activities against four strains, including MRSA, in vitro. The binding affinity and binding mode between the derivatives and 50S ribosome were studied by surface plasmon resonance (SPR) and molecular docking. Compound **9** was evaluated for in vivo activities against MRSA. Moreover, the degrees of inhibition from the derivatives on CYP3A4 were also evaluated.

## 2. Results and Discussion

### 2.1. Chemistry

**Scheme 1:**
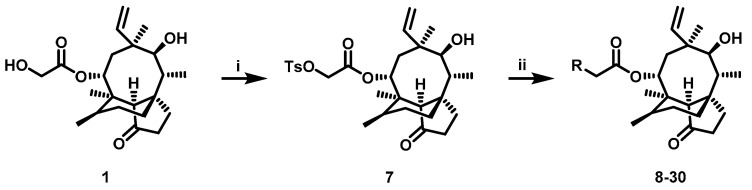
Reagent and conditions: (i) p-toluenesulfonyl chloride, ethyl acetate, NaOH, 0 °C, 3 h. (ii) a. R_1_R_2_HN, K_2_CO_3_, acetonitrile, 70 °C; b. TFA/DCM, room temperature.

The pleuromutilin derivatives were constructed by utilizing the same overall synthetic strategy as previously published (Scheme 1) [18]. All those pleuromutilin derivatives were purified by silica column chromatography to obtain the pure compounds. The synthesis of pleuromutilin derivatives **8**–**30** yield for the purified compounds of 41–86%. All derivatives were fully characterized by means of HR-MS, 1H-NMR and 13C-NMR spectral analysis. HPLC was conducted using a Waters e2695 liquid chromatography column (Phenomenex 4.6 × 250 mm, 5 μm, mobile phase A: 0.1% formic acid in water; mobile phase B: methanol; mobile phase C: acetonitrile). All compounds were assessed for purity by this reverse-column HPLC method (photodiode-array detection at wavelengths of 205 and 245 nm and evaporative light scattering detection at temperature of 60 °C) and shown to have purity >95%. The characterization spectrums of synthesized compounds were shown in the supplementary materials.

### 2.2. In Vitro Antibacterial Activity

The synthesized pleuromutilin derivatives **8**–**30** were tested against four Gram-positive bacteria (MRSA ATCC 43300, *S. aureus* ATCC 29213 and two clinical strains of *S. aureus* (AD3 and 144) isolated from Guangdong Province). Tiamulin was used as the positive control in this experiment. The minimum inhibitory concentration (MIC) values of the pleuromutilin derivatives **8**–**30** and tiamulin were determined using the broth dilution methods according to the Clinical and Laboratory Standards Institute (CLSI) [20]. The results of these studies are summarized in Table 1.

**Figure 2:**
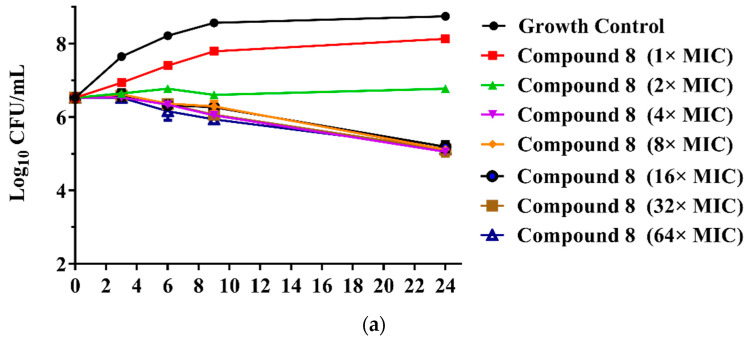

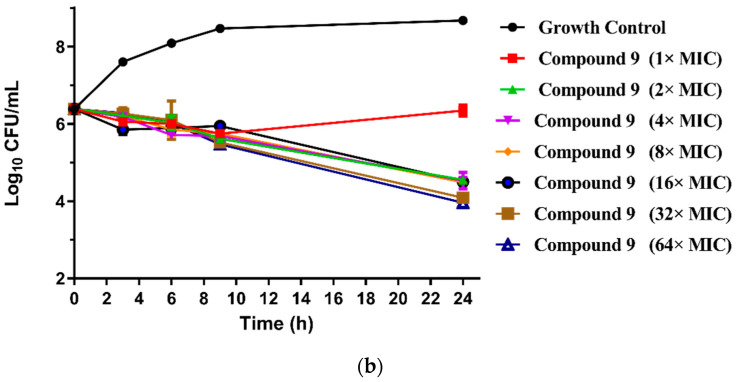
(**a**) Time-kill curves for MRSA ATCC 43300 with different concentrations of compounds **8**; (**b**) time-kill curves for MRSA ATCC 43300 with different concentrations of compounds **9**.

The MICs of the 23 new pleuromutilin derivatives against MRSA, *S. aureus*, 144 and AD3 ranged from 0.03 to 32 μg/mL, 0.125 to 32 μg/mL, 0.25 to ≥32 μg/mL and 0.125 to 32 μg/mL, respectively. Both compounds **8** and **9** displayed lower MIC values against MRSA compared with tiamulin. These results demonstrated that derivatives containing fluoro-benzene rings exhibited good antibacterial activity against MRSA compared with the methyl substituent group, which is consistent with previous research [21,22]. The different electron-attracting effect of halogen atoms might cause the different antibacterial activities of compounds **8**–**13**. In addition, it is easy to form hydrogen bonds with the target as the small radius of the fluorine atom. Therefore, the antibacterial activity of these two compounds was studied in depth through the time-kill curve experiment. The results are shown in Figure 2.

The results are presented in terms of the log_10_ CFU/mL change. Data are based on the conventional bactericidal activity standard, a 3 log_10_ CFU/mL or greater reduction in the viable colony count [23]. Compounds **8** and **9** showed a bactericidal effect on MRSA and killed 99.9% of MRSA at 4× MIC and 2× MIC concentrations in 9 h, respectively. On the contrary, there was a net growth of all the test isolates when subjected to 1× MIC of the compounds [24]. Growth inhibition and efficacy of both pleuromutilin derivatives were observed to be dose and time dependent, producing distinct time-kill profiles for the tested bacteria [23].

As an important, well-established pharmacodynamic parameter, post-antibiotic effect (PAE) refers to a period of time after the antibiotic has been completely removed from the culture medium with continual inhibition of bacterial growth [25]. A long PAE antibiotic might have a favorable outcome in clinical use, such as the ability to have to a less-frequent interval of administration, potentially reduced treatment cost and inhibition of the development of drug resistance [26]. Thus, the PAE was usually used to choose the antibiotic dosing regiments, optimize the treatment regimen as well as minimize the drug-induced side effects in clinical use [25].

**Figure 3:**
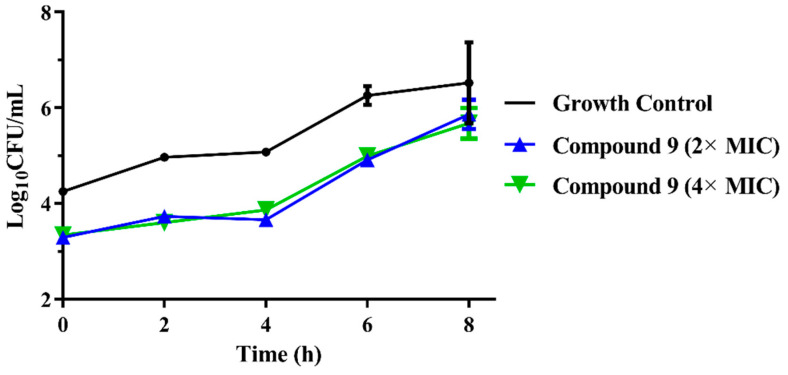
The bacterial growth kinetic curves for MRSA ATCC 43300 exposed to compound **9** with concentrations of 2× MIC and 4× MIC for 2 h.

To evaluate the in vitro antibacterial pharmacodynamic activity of compound **9**, we investigated the PAE of compound **9**. The results of the PAEs of compound **9** against MRSA are shown in Figure 3. Following exposure to 2× MIC and 4× MIC for 2 h, the PAE of compound **9** was 1.54 and 1.71 h. These results indicated that compound **9** displayed a concentration-independent PAE and possessed a similar PAE as tiamulin (2× MIC for 1.65 h and 4× MIC for 2.04 h) [27]. Thus, the results of the PAE might guide us in the rational use of compound **9** in future clinical practice and the evaluation of adverse reactions of antibiotics and combined use [25].

### 2.3. Binding Mode Characterization

#### 2.3.1. SPR Measurements

It was reported by Schluenzen et al. that the crystal structure of the 50S ribosomal subunit was in complex with the tiamulin [28]. Their results showed that tiamulin bound to the peptidyl transferase center (PTC) of the 50S ribosomal subunit. In order to investigate the interaction between the 50S ribosome and our pleuromutilin derivatives, an SPR-based assay for the 50S ribosome was established. The results are shown in Table 2.

The binding of each compound to the 50S ribosome during each cycle was represented by the response unit (RU) of surface resonance [29]. The association rate constants (K_a_) demonstrated the speed of the binding reaction. The dissociation rate constant (K_d_) demonstrated the dissociate rate. The equilibrium dissociation constant (K_D_) demonstrated the degree of dissociation of the compounds and the 50S ribosome in the equilibrium state [30]. A low K_D_ value indicated that the binding affinity between the compound and the 50S ribosome was strong [31].

The results revealed that the selected compounds bound reversibly to the 50S ribosome with clear association and dissociation phases. Among all these compounds, compound **9** (K_D_ = 1.77 × 10^−8^ M) showed higher intensity than tiamulin (K_D_ = 2.50 × 10^−8^ M). Based on the SPR results, the binding affinities of compound **9** were in agreement with the biological results, revealing that there might be a rational correlation between the antibacterial activity and binding free energy.

#### 2.3.2. Docking Studies

**Figure 4:**
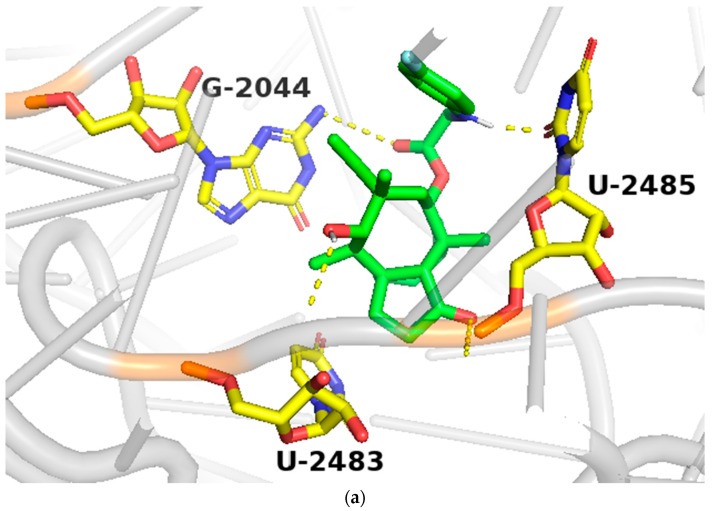

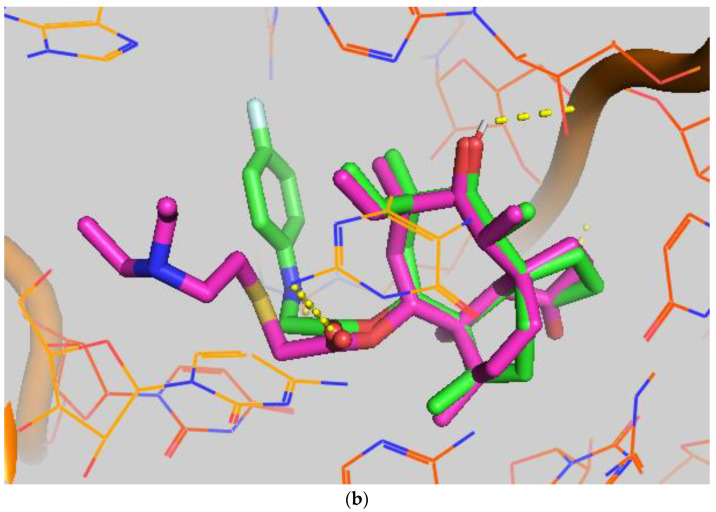
(**a**) The simulated docking modes of compounds **9** (green) to 1XBP; (**b**) the simulated docking modes of compounds **9** (green) and tiamulin (purple) to 1XBP. (Software: AutoDock Vina).

In order to study the binding of pleuromutilin derivatives with ribosomes, compound **9** was selected for molecular docking experiments [32]. The highest binding free energies (ΔGb, kcal/mol) and the lowest root-mean-square difference (RMSD) values were considered as the rational parameters to evaluate the docking results [33]. The binding free energy of compound **9** with the 50S ribosome was calculated to be −9.7 kcal/mol (RMSD = 0.596 Å). As shown in Figure 4, three strong hydrogen bonds were formed through the interaction of compound **9** with U-2485 (O/NH distance: 1.7 Å), U-2483 (OH/O distance: 1.9 Å) and G-2044 (NH/O distance: 2.2 Å). The binding free energy of tiamulin with the 50S ribosome was calculated to be −8.5 kcal/mol. According to the binding mode, the C-14 side chain showed a different degree of angle between compound **9** and tiamulin. Compound **9** showed stronger binding activity than tiamulin in this experiment. These results were consistent with the binding affinity in SPR and the MIC results of the in vitro experiment. In summary, compound **9** has strong binding activity with the 50S ribosome supported by binding mode characterization [19].

### 2.4. Neutropenic Murine Thigh Infection Model

**Figure 5:**
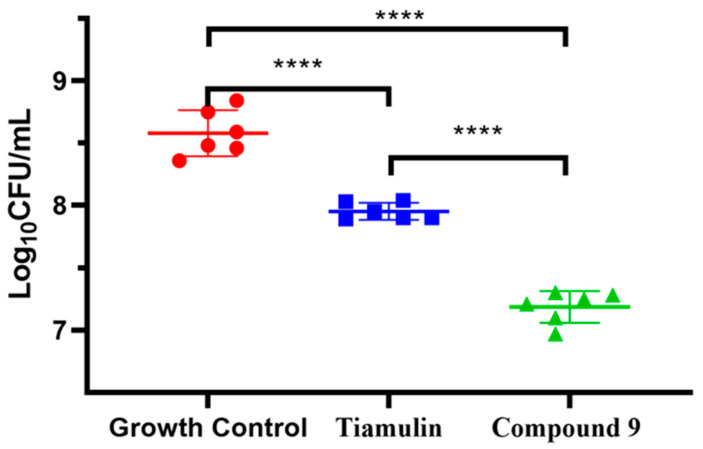
In vivo efficacy of Tiamulin (20 mg/kg) and compound **9** (20 mg/kg) in neutropenic mice thigh infection model. The mean log10 CFU is plotted of mice thigh after treatment with Tiamulin and compound **9**. The **** means significant difference.

Compound **9** was evaluated for in vivo antibacterial pharmacodynamic activity using a neutropenic murine thigh infection model. Mice groups treated with saline and tiamulin were chosen as the negative and positive control. As shown in Figure 5, the group treated with compound **9** at 20 mg/kg has a significantly reduced MRSA load (~1.3 log_10_ CFU/mL) in the thigh compared to the no-drug control group (*p* < 0.001, *n* = 6/group). Tiamulin at 20 mg/kg also showed a treatment effect (~0.6 log_10_ CFU/mL) against MRSA in the thigh compared to the no-drug control group (*p* < 0.001, *n* = 6/group). These results revealed that compound **9** was able to relieve MRSA infection in vivo and was more effective than tiamulin in reducing the MRSA load in thigh-infected mice (~0.7 log_10_ CFU/mL, *p* < 0.001, *n* = 6/group).

### 2.5. Effect on Liver Microsomal CYP3A4 Enzyme Activity

**Figure 6:**
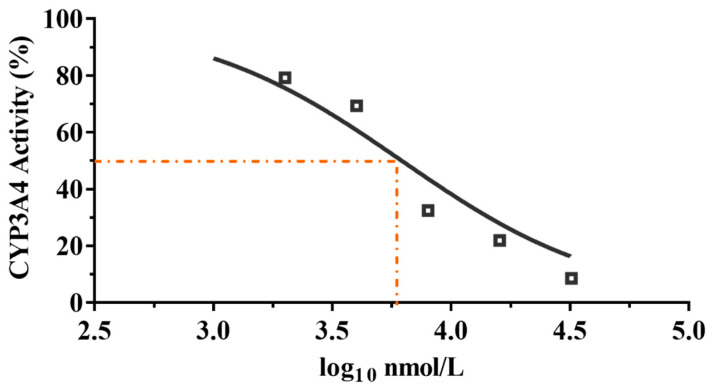
CYP3A4 inhibition by compound **9** with different concentrations.

Azamulin was reported to have a potency inhibitory effect against CYP3A4, causing the development to be discontinued after Phase I trials [34]. To determine whether compound **9** is used suitably as a candidate drug, we tested the inhibitory effect of compound **9** on liver microsomal CYP3A4. The results are shown in Figure 6.

It is commonly accepted that compounds with IC_50_ values higher than 10 μM against CYP3A4 enzyme are weak CYP inhibitors; compounds with 3 μM < IC_50_ < 10 μM are moderate CYP inhibitors, while compounds with IC_50_ < 3 μM are strong CYP inhibitors [27]. As shown in Figure 6, compound **9** displayed a moderate inhibitory effect on CYP3A4 enzyme (IC_50_ = 6.19 μM, MW= 471.28, IC_50_ = 2.92 μg/mL), whereas tiamulin (IC_50_ = 1.64 μM, MW = 493.74, IC_50_ = 0.81 μg/mL) and azamulin (IC_50_ = 0.03–0.24 μM, MW = 478.62, IC_50_ = 0.01–0.11 μg/mL) showed a strong inhibitory effect on CYP3A4 [27]. The results showed that the inhibitory rate of compound **9** on CYP3A4 was much lower than that of tiamulin and azamulin. The results indicated the safety of compound **9** compared with tiamulin and azamulin when combined with drugs mainly metabolized by the CYP3A4 enzyme [35,36]. However, to minimize the side effects of compound **9**, the inhibition of CYP3A4 in vivo is necessary to be evaluated in the future.

Since pleuromutilin was first isolated from two basidiomycete species in 1951, thousands of pleuromutilin derivatives have been synthesized [18]. Most studies have shown that modification of the C-14 position of pleuromutilin could led to compounds with improved antimicrobial activity [19]. All marketed drugs of pleuromutilin use thioether substitutions at the C-22 position [13]. Additionally, the de novo syntheses of analogues of pleuromutilin, which allow the core of the pleuromutilin to be varied, have been reported recently [37]. Similarly, there are other classes of antibiotics that also act on bacterial ribosomes, such as macrolides. Macrolides have more established drugs than pleuromutilins, including erythromycin, clarithromycin, azithromycin, telithromycin and cethromycin [38]. However, the extensive application of macrolide antibiotics culminated in a relentless advance of microbial resistance [39]. In addition, enzymes have also been used in antibacterial research in recent years, such as protease HtrA against *S.aureus* [40]. In order to develop anti-MRSA antibiotics and novel pleuromutilin derivatives, our group had many achievements in the amino-substituted pleuromutilin derivatives at C-22 position. We will continue to focus on such derivatives and hope to develop a marketable drug.

## 3. Conclusions

A series of 23 new pleuromutilin derivatives were designed and synthesized in the present work. Compound **9** (MIC = 0.06 μg/mL, against MRSA) showed better antibacterial activity than tiamulin. The resulting time-kill curve experiments indicated that compound **9** was a time-dependent antibacterial agent and had a certain inhibitory effect against MRSA in vitro. In the SPR study, compound **9** showed strong affinity to the 50S ribosome (K_D_ = 1.77 × 10^−8^ M). The molecular docking study also proved strong binding free energies between compound **9** and residues of the 50S ribosome (−9.7 kcal/mol, 3 hydrogen bonds). The results of the neutropenic murine thigh infection model indicated that compound **9** displayed a better in vivo antibacterial effect than tiamulin (~0.7 log_10_ CFU/mL, *p* < 0.001, *n* = 6/group). Meanwhile, the results of CYP3A4 inhibition experiments showed that the degree of inhibition from compound **9** (IC_50_ = 2.92 μg/mL) was lower than that of tiamulin (IC_50_ = 0.81 μg/mL). The in vitro, in vivo, SPR, docking, and CYP3A4 inhibition studies reported here indicated that compound **9** might serve as a possible lead compound for the treatment of infections in clinical use.

## 4. Experimental Section

### 4.1. Materials

The raw material pleuromutilin (>90% pure) was purchased from Great Enjoyhood Biochemical Co. Ltd., (Daying, China). The other reagents were analytical grade and obtained from Guangzhou Chemical Reagent Factory (Guangzhou, China). The target compounds were purified by column chromatography (silica gel, 100–200 mesh, Branch of Qingdao Haiyang Chemical Co. Ltd., Shandong, China). 1H-NMR and 13C-NMR spectra in CDCl_3_ were measured on Bruker AV-400 or Bruker AV-600 spectrometer (Bruker, Karlsruhe, Germany). The chemical shift values (δ) are reported in ppm relative to tetramethylsilane as an internal standard. High-resolution mass spectra were recorded by Thermo Fisher Scientific QE-Fouces (Thermo Fisher Scientific, Shanghai, China) with an electro spray ionization (ESI) source.

### 4.2. Synthesis

A general synthetic route based on the compound 22-O-tosylpleuromutilin (compound **7**) and nitrogen-containing derivatives were used to prepare a series of pleuromutilin derivatives.

#### 4.2.1. Synthesis of 22-O-Tosylpleuromutilin (**7**)

A solution of pleuromutilin **1** (12.0 g, 31.71 mmol) in ethyl acetate (30.0 mL) in a three-necked round-bottom flask was stirred at 0 °C, and p-toluenesulfonyl chloride (6.6 g, 34.87 mmol) was added. Sodium hydroxide (2.5 g, 63.41 mmol) was dissolved in 12 mL of water and dropped into the solution. The mixture was stirred at room temperature until the consumption of pleuromutilin was obtained completely. CHCl_3_ (60 mL) and ice water (60 mL) were added to the solution. The organic phase was washed with brine (20 mL) and water (20 mL). Then, the bottom layer (organic phase) was dried over anhydrous Na_2_SO_4_ and filtered. The phase was concentrated in vacuo to give a white solid (12.2 g, 88.41%).

#### 4.2.2. General Procedure for the Synthesis of Compounds **8**–**30**

Compound **7** (1 g, 1.87 mmol) was dissolved in acetonitrile (15 mL) and sodium iodide (0.32 g, 2.09 mmol) was added and stirred at 83 °C for 1 h. N-group (2.09 mmol) and potassium carbonate (0.51 g, 3.76 mmol) were added to the mixture and stirred again for 6 h at 83 °C. Chloroform (25 mL) was added, and then the mixture was washed with water (25 mL). The organic phase was dried over anhydrous Na_2_SO_4_, filtered and concentrated under reduced pressure to give the crude oil. The crude oil was purified by column chromatography (dichloromethane: methanol = 20:1) to give the target compound. The purity of compounds **8**–**20** was determined to be >95% using HPLC analyses (SHIMADZU, Kyoto, Japan). The characterization spectrum of synthesized compounds see the supplementary materials.

#### 4.2.3. 22-[(4-(Benzylideneamino)-5-methyl-4H-1,2,4-triazol-3-yl) Thio] Deoxy Pleuromutilin (**8**)

White powder; yield: 86%; mp: 153–154 °C; ^1^H NMR (400 MHz, Chloroform-*d*) δ7.11 (1 H, dd, *J =* 8.1, 6.6 Hz), 6.51–6.40 (2 H, m), 6.47 (1 H, dd, *J =* 17.4, 11.0 Hz, H19), 6.27 (1 H, dd, *J =* 11.3, 2.4 Hz), 5.82 (1 H, d, *J =* 8.5 Hz, H14), 5.34 (2 H, s, H20), 5.20 (1 H, d, *J =* 17.3 Hz, H20), 3.78 (2 H, m, H22, H11), 3.37 (1 H, dd, *J* = 10.1, 6.5 Hz), 2.23 (5 H, dd, *J =* 25.7, 15.5, 8.3 Hz, H2, H4, H10, H13), 1.97 (1 H, dd, *J =* 16.1, 8.6 Hz, 11-OH), 1.75 (3 H, d, *J =* 14.7 Hz, H6, H8), 1.66–1.57 (4 H, m, H1, H7, H13), 1.36 (3 H, s, H15), 1.27 (2 H, d, *J =* 13.0 Hz, H8), 1.10 (3 H, s, H18), 0.89 (3 H, d, *J =* 6.9 Hz, H17), 0.72 (3 H, d, *J =* 7.0 Hz, H16). ^13^C NMR (101 MHz, Chloroform-*d*) δ 216.89 (C3), 169.24 (C21), 139.06 (C19), 130.43, 130.36, 117.19 (C20), 115.81, 115.66, 113.98, 113.93, 74.54 (C11), 70.56 (C14), 58.00 (C4), 53.42 (C22), 45.39 (C9), 44.60 (C13), 43.99 (C12), 41.73 (C5), 36.55 (C6), 35.97 (C2), 30.31 (C8), 26.81 (C7), 26.37 (C18), 24.78 (C1),16.53 (C16), 14.77 (C15), 11.53 (C17). HR-MS (ESI): Calcd for C_28_H_38_FNO_4_ (M + H^+^): 472.2863; Found: 472.2846.

#### 4.2.4. 22-(4-Fluoroanilineyl)-22-deoxypleuromutilin (**9**)

White powder; yield: 88%; mp: 91–93 °C; ^1^H NMR (400 MHz, Chloroform-*d*) δ6.93–6.86 (2 H, m), 6.56–6.52 (2 H, m), 6.47 (1 H, dd, *J =* 17.4, 11.0 Hz, H19), 5.82 (1 H, d, *J =* 8.5 Hz, H14), 5.34 (2 H, s, H20), 5.20 (1 H, d, *J =* 17.3 Hz, H20), 3.78 (2 H, m, H22, H11), 3.37 (1 H, dd, *J* = 10.1, 6.5 Hz),2.23 (5 H, dd, *J* = 25.7, 15.5, 8.3 Hz, H2, H4, H10, H13), 1.97 (1 H, dd, *J* = 16.1, 8.6 Hz, 11-OH), 1.75 (3 H, d, *J =* 14.7 Hz, H6,H8), 1.66–1.57 (4 H, m, H1,H7, H13), 1.36 (3 H, s, H15), 1.27 (2 H, d, *J =* 13.0 Hz, H8), 1.10 (3 H, s, H18), 0.89 (3 H, d, *J =* 6.9 Hz, H17), 0.72 (3 H, d, *J =* 7.0 Hz, H16). ^13^C NMR (101 MHz, Chloroform-*d*) δ 216.89 (C3), 169.24 (C21), 139.06 (C19), 130.43, 130.36, 117.19 (C20), 115.81, 115.66, 113.98, 113.93, 74.54 (C11), 70.56 (C14), 58.00 (C4), 53.42 (C22), 45.39 (C9), 44.60 (C13), 43.99 (C12), 41.73 (C5), 36.55 (C6), 35.97 (C2), 30.31 (C8), 26.81 (C7), 26.37 (C18), 24.78 (C1),16.53 (C16), 14.77 (C15), 11.53 (C17). HR-MS (ESI): Calcd for C_28_H_38_FNO_4_ (M + H^+^): 472.2863; Found: 472.2846.

#### 4.2.5. 22-(3-Chloroanilineyl)-22-deoxypleuromutilin (**10**)

White powder; yield: 73%; mp: 158–160 °C; ^1^H NMR (400 MHz, Chloroform-*d*) δ7.08–6.86 (2 H, m), 6.56–6.49 (2 H, m), 6.47 (1 H, dd, *J =* 17.4, 11.0 Hz, H19), 5.83 (1 H, d, *J =* 8.5 Hz, H14), 5.34 (2 H, s, H20), 5.20 (1 H, d, *J =* 17.3 Hz, H20), 3.78 (2 H, m, H22,H11), 3.37 (1 H, dd, *J* = 10.1, 6.5 Hz), 2.23 (5 H, dd, *J =* 25.7, 15.5, 8.3 Hz, H2, H4, H10, H13), 1.97 (1 H, dd, *J =* 16.1, 8.6 Hz, 11-OH), 1.75 (3 H, d, *J =* 14.7 Hz, H6, H8), 1.66–1.57 (4 H, m, H1, H7,H13), 1.36 (3 H, s, H15), 1.27 (2 H, d, *J =* 13.0 Hz, H8), 1.10 (3 H, s, H18), 0.89 (3 H, d, *J =* 6.9 Hz, H17), 0.72 (3 H, d, *J =* 7.0 Hz, H16). ^13^C NMR (101 MHz, Chloroform-*d*) δ 216.89 (C3), 169.24 (C21), 139.06 (C19), 130.43, 130.36, 117.19 (C20), 115.81, 115.66, 113.98, 113.93, 74.54 (C11), 70.56 (C14), 58.00 (C4), 53.42 (C22), 45.39 (C9), 44.60 (C13), 43.99 (C12), 41.73 (C5), 36.55 (C6), 35.97 (C2), 30.31 (C8), 26.81 (C7), 26.37 (C18), 24.78 (C1),16.53 (C16), 14.77 (C15), 11.53 (C17). HR-MS (ESI): Calcd for C_28_H_38_ClNO_4_ (M + H^+^): 488.2567; Found: 488.2554.

#### 4.2.6. 22-(4-Chloroanilineyl)-22-deoxypleuromutilin (**11**)

White powder; yield: 78%; mp: 94–95 °C; ^1^H NMR (400 MHz, Chloroform-*d*) δ7.15–7.11 (2 H, m), 6.53–6.50 (2 H, m), 6.47 (1 H, dd, *J =* 17.4, 11.0 Hz, H19), 5.83 (1 H, d, *J =* 8.5 Hz, H14), 5.34 (2 H, s, H20), 5.20 (1 H, d, *J =* 17.3 Hz, H20), 3.78 (2 H, m, H22,H11), 3.37 (1 H, dd, *J* = 10.1, 6.5 Hz), 2.23 (5 H, ddd, *J =* 25.7, 15.5, 8.3 Hz, H2,H4,H10,H13), 1.97 (1 H, dd, *J =* 16.1, 8.6 Hz, 11-OH), 1.75 (3 H, d, *J =* 14.7 Hz, H6,H8), 1.66–1.57 (4 H, m, H1, H7, H13), 1.36 (3 H, s, H15), 1.27 (2 H, d, *J =* 13.0 Hz, H8), 1.10 (3 H, s, H18), 0.89 (3 H, d, *J =* 6.9 Hz, H17), 0.72 (3 H, d, *J =* 7.0 Hz, H16). ^13^C NMR (101 MHz, Chloroform-*d*) δ 216.89 (C3), 169.24 (C21), 139.06 (C19), 130.43, 130.36, 117.19 (C20), 115.81, 115.66, 113.98, 113.93, 74.54 (C11), 70.56 (C14), 58.00 (C4), 53.42 (C22), 45.39 (C9), 44.60 (C13), 43.99 (C12), 41.73 (C5), 36.55 (C6), 35.97 (C2), 30.31 (C8), 26.81 (C7), 26.37 (C18), 24.78 (C1),16.53 (C16), 14.77 (C15), 11.53 (C17). HR-MS (ESI): Calcd for C_28_H_38_ClNO_4_ (M + H^+^): 488.2567; Found: 488.2555.

#### 4.2.7. 22-(3-Bromoanilineyl)-22-deoxypleuromutilin (**12**)

White powder; yield: 62%; mp: 161–163 °C; ^1^H NMR (400 MHz, Chloroform-*d*) δ7.01 (1 H, t, *J =* 8.0, 10.5 Hz), 6.85(1 H, ddd, *J =* 7.8, 1.8 Hz), 6.56–6.52 (2 H, m), 6.47 (1 H, dd, *J =* 17.4, 11.0 Hz, H19), 5.82 (1 H, d, *J =* 8.5 Hz, H14), 5.34 (2 H, s, H20), 5.20 (1 H, d, *J =* 17.3 Hz, H20), 3.78 (2 H, m, H22,H11), 3.37 (1 H, dd, *J* = 10.1, 6.5 Hz), 2.23 (5 H, dd, *J =* 25.7, 15.5, 8.3 Hz, H2, H4, H10, H13), 1.97 (1 H, dd, *J =* 16.1, 8.6 Hz, 11-OH), 1.75 (3 H, d, *J =* 14.7 Hz, H6,H8), 1.66–1.57 (4 H, m, H1, H7, H13), 1.36 (3 H, s, H15), 1.27 (2 H, d, *J =* 13.0 Hz, H8), 1.10 (3 H, s, H18), 0.89 (3 H, d, *J =* 6.9 Hz, H17), 0.72 (3 H, d, *J =* 7.0 Hz, H16). ^13^C NMR (101 MHz, Chloroform-*d*) δ 216.89 (C3), 169.24 (C21), 139.06 (C19), 130.43, 130.36, 117.19 (C20), 115.81, 115.66, 113.98, 113.93, 74.54 (C11), 70.56 (C14), 58.00 (C4), 53.42 (C22), 45.39 (C9), 44.60 (C13), 43.99 (C12), 41.73 (C5), 36.55 (C6), 35.97 (C2), 30.31 (C8), 26.81 (C7), 26.37 (C18), 24.78 (C1),16.53 (C16), 14.77 (C15), 11.53 (C17). HR-MS (ESI): Calcd for C_28_H_38_BrNO_4_ (M + H^+^): 532.2062; Found: 532.2048.

#### 4.2.8. 22-(4-Bromoanilineyl)-22-deoxypleuromutilin (**13**)

White powder; yield: 72%; mp: 97–98 °C; ^1^H NMR (400 MHz, Chloroform-*d*) δ7.12–6.89 (2 H, m), 6.65–6.58 (2 H, m), 6.47 (1 H, dd, *J =* 17.4, 11.0 Hz, H19), 5.82 (1 H, d, *J =* 8.5 Hz, H14), 5.34 (2 H, s, H20), 5.20 (1 H, d, *J =* 17.3 Hz, H20), 3.78 (2 H, m, H22, H11), 3.37 (1 H, dd, *J* = 10.1, 6.5 Hz), 2.23 (5 H, ddd, *J =* 25.7, 15.5, 8.3 Hz, H2, H4, H10, H13), 1.97 (1 H, dd, *J =* 16.1, 8.6 Hz, 11-OH), 1.75 (3 H, d, *J =* 14.7 Hz, H6,H8), 1.66–1.57 (4 H, m, H1, H7, H13), 1.36 (3 H, s, H15), 1.27 (2 H, d, *J =* 13.0 Hz, H8), 1.10 (3 H, s, H18), 0.89 (3 H, d, *J =* 6.9 Hz, H17), 0.72 (3 H, d, *J =* 7.0 Hz, H16). ^13^C NMR (101 MHz, Chloroform-*d*) δ 216.89 (C3), 169.24 (C21), 139.06 (C19), 130.43, 130.36, 117.19 (C20), 115.81, 115.66, 113.98, 113.93, 74.54 (C11), 70.56 (C14), 58.00 (C4), 53.42 (C22), 45.39 (C9), 44.60 (C13), 43.99 (C12), 41.73 (C5), 36.55 (C6), 35.97 (C2), 30.31 (C8), 26.81 (C7), 26.37 (C18), 24.78 (C1),16.53 (C16), 14.77 (C15), 11.53 (C17). HR-MS (ESI): Calcd for C_28_H_38_BrNO_4_ (M + H^+^): 532.2062; Found: 532.2047.

#### 4.2.9. 22-(2-Fluorobenzylamineyl)-22-deoxypleuromutilin (**14**)

White powder; yield: 62%; mp: 163–164 °C; ^1^H NMR (400 MHz, Chloroform-*d*) δ7.34 (1 H, t, *J =* 7.5, 1.8 Hz), 7.26–7.19 (1 H, m), 7.14-6.99 (2 H, m), 6.53 (1 H, dd, *J =* 17.4, 11.0 Hz, H19), 5.82 (1 H, d, *J =* 8.5 Hz, H14), 5.34 (2 H, s, H20), 5.20 (1 H, d, *J =* 17.3 Hz, H20), 3.93–3.74 (2 H, m),3.78 (2 H, m, H22,H11), 3.37 (1 H, dd, *J* = 10.1, 6.5 Hz), 2.23 (5 H, ddd, *J =* 25.7, 15.5, 8.3 Hz, H2, H4, H10, H13), 1.97 (1 H, dd, *J =* 16.1, 8.6 Hz, 11-OH), 1.75 (3 H, d, *J =* 14.7 Hz, H6,H8), 1.66–1.57 (4 H, m, H1,H7,H13), 1.36 (3 H, s, H15), 1.27 (2 H, d, *J =* 13.0 Hz, H8), 1.18 (3 H, s, H18), 0.89 (3 H, d, *J =* 6.9 Hz, H17), 0.72 (3 H, d, *J =* 7.0 Hz, H16). ^13^C NMR (101 MHz, Chloroform-*d*) δ 216.89 (C3), 169.24 (C21), 139.06 (C19), 130.43, 130.36, 117.19 (C20), 115.81, 115.66, 113.98, 113.93, 74.54 (C11), 70.56 (C14), 58.00 (C4), 53.42 (C22), 50.71, 45.39 (C9), 44.60 (C13), 43.99 (C12), 41.73 (C5), 36.55 (C6), 35.97 (C2), 30.31 (C8), 26.81 (C7), 26.37 (C18), 24.78 (C1),16.53 (C16), 14.77 (C15), 11.53 (C17). HR-MS (ESI): Calcd for C_29_H_40_FNO_4_ (M + H^+^): 486.3019; Found: 486.3005.

#### 4.2.10. 22-(3-Fluorobenzylamineyl)-22-deoxypleuromutilin (**15**)

White powder; yield: 70%; mp: 143–144 °C; ^1^H NMR (400 MHz, Chloroform-*d*) δ7.–7.01 (2 H, m), 6.56–6.52 (2 H, m), 6.47 (1 H, dd, *J =* 17.4, 11.0 Hz, H19), 5.82 (1 H, d, *J =* 8.5 Hz, H14), 5.34 (2 H, s, H20), 5.20 (1 H, d, *J =* 17.3 Hz, H20), 3.89–3.80 (2 H, m), 3.78 (2 H, m, H22, H11), 3.37 (1 H, dd, *J* = 10.1, 6.5 Hz), 2.23 (5 H, ddd, *J =* 25.7, 15.5, 8.3 Hz, H2, H4, H10, H13), 1.97 (1 H, dd, *J =* 16.1, 8.6 Hz, 11-OH), 1.75 (3 H, d, *J =* 14.7 Hz, H6, H8), 1.66–1.57 (4 H, m, H1,H7,H13), 1.36 (3 H, s, H15), 1.27 (2 H, d, *J =* 13.0 Hz, H8), 1.10 (3 H, s, H18), 0.89 (3 H, d, *J =* 6.9 Hz, H17), 0.72 (3 H, d, *J =* 7.0 Hz, H16). ^13^C NMR (101 MHz, Chloroform-*d*) δ 216.89 (C3), 169.24 (C21), 139.06 (C19), 130.43, 130.36, 117.19 (C20), 115.81, 115.66, 113.98, 113.93, 74.54 (C11), 70.56 (C14), 58.00 (C4), 53.42 (C22), 50.69, 45.39 (C9), 44.60 (C13), 43.99 (C12), 41.73 (C5), 36.55 (C6), 35.97 (C2), 30.31 (C8), 26.81 (C7), 26.37 (C18), 24.78 (C1),16.53 (C16), 14.77 (C15), 11.53 (C17). HR-MS (ESI): Calcd for C_29_H_40_FNO_4_ (M + H^+^): 486.3019; Found: 486.3005.

#### 4.2.11. 22-(4-Fluorobenzylamineyl)-22-deoxypleuromutilin (**16**)

White powder; yield: 73%; mp: 76–77 °C; ^1^H NMR (400 MHz, Chloroform-*d*) δ7.30–7.27 (2 H, m), 7.07–6.94 (2 H, m), 6.47 (1 H, dd, *J =* 17.4, 11.0 Hz, H19), 5.82 (1 H, d, *J =* 8.5 Hz, H14), 5.34 (2 H, s, H20), 5.20 (1 H, d, *J =* 17.3 Hz, H20), 3.78 (2 H, m, H22, H11), 3.37 (1 H, dd, *J* = 10.1, 6.5 Hz), 3.41–3.23 (2 H, m), 2.23 (5 H, ddd, *J =* 25.7, 15.5, 8.3 Hz, H2, H4, H10, H13), 1.97 (1 H, dd, *J =* 16.1, 8.6 Hz, 11-OH), 1.75 (3 H, d, *J =* 14.7 Hz, H6,H8), 1.66–1.57 (4 H, m, H1, H7, H13), 1.36 (3 H, s, H15), 1.27 (2 H, d, *J =* 13.0 Hz, H8), 1.10 (3 H, s, H18), 0.89 (3 H, d, *J =* 6.9 Hz, H17), 0.72 (3 H, d, *J =* 7.0 Hz, H16). ^13^C NMR (101 MHz, Chloroform-*d*) δ 216.89 (C3), 169.24 (C21), 139.06 (C19), 130.43, 130.36, 117.19 (C20), 115.81, 115.66, 113.98, 113.93, 74.54 (C11), 70.56 (C14), 58.00 (C4), 53.42 (C22), 50.72, 45.39 (C9), 44.60 (C13), 43.99 (C12), 41.73 (C5), 36.55 (C6), 35.97 (C2), 30.31 (C8), 26.81 (C7), 26.37 (C18), 24.78 (C1),16.53 (C16), 14.77 (C15), 11.53 (C17). HR-MS (ESI): Calcd for C_29_H_40_FNO_4_ (M + H^+^): 486.3019; Found: 486.3004.

#### 4.2.12. 22-(2-PhenylethylaMineyl)-22-deoxypleuromutilin (**17**)

White powder; yield: 78%; mp: 73–75 °C; ^1^H NMR (400 MHz, Chloroform-*d*) δ7.31–7.21 (2 H, m), 7.03–6.94 (3 H, m), 6.47 (1 H, dd, *J =* 17.4, 11.0 Hz, H19), 5.82 (1 H, d, *J =* 8.5 Hz, H14), 5.34 (2 H, s, H20), 5.20 (1 H, d, *J =* 17.3 Hz, H20), 3.85–3.67 (2 H, m), 3.78 (2 H, m, H22, H11), 3.37 (1 H, dd, *J* = 10.1, 6.5 Hz), 3.44–3.30 (2 H, m), 2.23 (5 H, ddd, *J =* 25.7, 15.5, 8.3 Hz, H2, H4, H10, H13), 1.97 (1 H, dd, *J =* 16.1, 8.6 Hz, 11-OH), 1.75 (3 H, d, *J =* 14.7 Hz, H6, H8), 1.66–1.57 (4 H, m, H1, H7, H13), 1.36 (3 H, s, H15), 1.27 (2 H, d, *J =* 13.0 Hz, H8), 1.10 (3 H, s, H18), 0.89 (3 H, d, *J =* 6.9 Hz, H17), 0.72 (3 H, d, *J =* 7.0 Hz, H16). ^13^C NMR (101 MHz, Chloroform-*d*) δ 216.89 (C3), 169.24 (C21), 139.06 (C19), 130.43, 130.36, 117.19 (C20), 115.81, 115.66, 113.98, 113.93, 74.54 (C11), 70.56 (C14), 58.00 (C4), 53.42 (C22), 50.71, 45.39 (C9), 44.60 (C13), 43.99 (C12), 41.73 (C5), 38.26, 36.55 (C6), 35.97 (C2), 30.31 (C8), 26.81 (C7), 26.37 (C18), 24.78 (C1),16.53 (C16), 14.77 (C15), 11.53 (C17). HR-MS (ESI): Calcd for C_30_H_43_NO_4_ (M + H^+^): 482.3270; Found: 482.3253.

#### 4.2.13. 22-(2-Methylphenethylamineyl)-22-deoxypleuromutilin (**18**)

White powder; yield: 70%; mp: 91–93 °C; ^1^H NMR (400 MHz, Chloroform-*d*) δ7.29–7.27 (2 H, m), 7.22–7.15 (2 H, m), 6.53 (1 H, dd, *J =* 17.4, 11.0 Hz, H19), 5.82 (1 H, d, *J =* 8.5 Hz, H14), 5.34 (2 H, s, H20), 5.20 (1 H, d, *J =* 17.3 Hz, H20), 3.85–3.67 (2 H, m), 3.78 (2 H, m, H22, H11), 3.37 (1 H, dd, *J* = 10.1, 6.5 Hz), 3.44–3.30 (2 H, m), 2.70–2.62 (3 H, m), 2.23 (5 H, ddd, *J =* 25.7, 15.5, 8.3 Hz, H2, H4, H10, H13), 1.97 (1 H, dd, *J =* 16.1, 8.6 Hz, 11-OH), 1.75 (3 H, d, *J =* 14.7 Hz, H6, H8), 1.66–1.57 (4 H, m, H1, H7, H13), 1.36 (3 H, s, H15), 1.27 (2 H, d, *J =* 13.0 Hz, H8), 1.10 (3 H, s, H18), 0.89 (3 H, d, *J =* 6.9 Hz, H17), 0.72 (3 H, d, *J =* 7.0 Hz, H16). ^13^C NMR (101 MHz, Chloroform-*d*) δ 216.89 (C3), 169.24 (C21), 139.06 (C19), 130.43, 130.36, 117.19 (C20), 115.81, 115.66, 113.98, 113.93, 74.54 (C11), 70.56 (C14), 58.00 (C4), 53.42 (C22), 50.65, 45.39 (C9), 44.60 (C13), 43.99 (C12), 41.73 (C5), 39.62, 38.12, 36.55 (C6), 35.97 (C2), 30.31 (C8), 26.81 (C7), 26.37 (C18), 24.78 (C1),16.53 (C16), 14.77 (C15), 11.53 (C17). HR-MS (ESI): Calcd for C_31_H_45_NO_4_ (M + H^+^): 496.3427; Found: 496.3409.

#### 4.2.14. 22-(3-Phenylpropylamineyl)-22-deoxypleuromutilin (**19**)

White powder; yield: 76%; mp: 83–84 °C; ^1^H NMR (400 MHz, Chloroform-*d*) δ7.21–7.15 (2 H, m), 7.03–6.94 (3 H, m), 6.47 (1 H, dd, *J =* 17.4, 11.0 Hz, H19), 5.82 (1 H, d, *J =* 8.5 Hz, H14), 5.34 (2 H, s, H20), 5.20 (1 H, d, *J =* 17.3 Hz, H20), 3.85–3.67 (2 H, m), 3.78 (2 H, m, H22, H11), 3.37 (1 H, dd, *J* = 10.1, 6.5 Hz), 3.44–3.30 (2 H, m),2.87–2.74 (2 H, m) 2.23 (5 H, ddd, *J =* 25.7, 15.5, 8.3 Hz, H2, H4, H10, H13), 1.97 (1 H, dd, *J =* 16.1, 8.6 Hz, 11-OH), 1.75 (3 H, d, *J =* 14.7 Hz, H6, H8), 1.66–1.57 (4 H, m, H1, H7, H13), 1.36 (3 H, s, H15), 1.27 (2 H, d, *J =* 13.0 Hz, H8), 1.10 (3 H, s, H18), 0.89 (3 H, d, *J =* 6.9 Hz, H17), 0.72 (3 H, d, *J =* 7.0 Hz, H16). ^13^C NMR (101 MHz, Chloroform-*d*) δ 216.89 (C3), 169.24 (C21), 139.06 (C19), 130.43, 130.36, 117.19 (C20), 115.81, 115.66, 113.98, 113.93, 78.63, 74.54 (C11), 70.56 (C14), 58.00 (C4), 53.42 (C22), 50.14, 45.39 (C9), 44.60 (C13), 43.99 (C12), 41.73 (C5), 38.32, 36.55 (C6), 35.97 (C2), 30.31 (C8), 26.81 (C7), 26.37 (C18), 24.78 (C1),16.53 (C16), 14.77 (C15), 11.53 (C17). HR-MS (ESI): Calcd for C_31_H_45_NO_4_ (M + H^+^): 496.3427; Found: 496.3409.

#### 4.2.15. 22-(Thiophene-2-ethylamineyl)-22-deoxypleuromutilin (**20**)

White powder; yield: 77%; mp: 69–70 °C; ^1^H NMR (400 MHz, Chloroform-*d*) δ7.14 (1 H, m), 6.93 (1 H, m), 6.84 (1 H, m), 6.47 (1 H, dd, *J =* 17.4, 11.0 Hz, H19), 5.82 (1 H, d, *J =* 8.5 Hz, H14), 5.34 (2 H, s, H20), 5.20 (1 H, d, *J =* 17.3 Hz, H20), 3.78 (2 H, m, H22,H11), 3.37 (1 H, dd, *J* = 10.1, 6.5 Hz), 2.23 (5 H, ddd, *J =* 25.7, 15.5, 8.3 Hz, H2, H4, H10, H13), 1.97 (1 H, dd, *J =* 16.1, 8.6 Hz, 11-OH), 1.75 (3 H, d, *J =* 14.7 Hz, H6, H8), 1.66–1.57 (4 H, m, H1, H7, H13), 1.36 (3 H, s, H15), 1.27 (2 H, d, *J =* 13.0 Hz, H8), 1.10 (3 H, s, H18), 0.88 (3 H, d, *J =* 6.9 Hz, H17), 0.71 (3 H, d, *J =* 7.0 Hz, H16). ^13^C NMR (101 MHz, Chloroform-*d*) δ 216.89 (C3), 169.24 (C21), 143.24, 142.10, 139.06 (C19), 126.87, 125.03, 117.19 (C20), 77.23, 76.81, 74.54 (C11), 70.56 (C14), 58.00 (C4), 53.42 (C22), 45.39 (C9), 44.60 (C13), 43.99 (C12), 41.73 (C5), 36.55 (C6), 35.97 (C2), 30.31 (C8), 26.81 (C7), 26.37 (C18), 24.78 (C1),16.53 (C16), 14.77 (C15), 11.53 (C17). HR-MS (ESI): Calcd for C_28_H_41_NO_4_S (M + H^+^): 488.2834; Found: 488.2819.

#### 4.2.16. 22-Butylamineyl-22-deoxypleuromutilin (**21**)

White powder; yield: 70%; mp: 74–75 °C; ^1^H NMR (400 MHz, Chloroform-*d*) δ 6.53 (1 H, dd, *J =* 17.4, 11.0 Hz, H19), 5.80 (1 H, d, *J =* 8.5 Hz, H14), 5.36 (2 H, s, H20), 5.21 (1 H, d, *J =* 17.3, 6.5 Hz, H20), 3.78 (2 H, m, H22, H11), 3.37 (1 H, dd, *J* = 10.1, 6.5 Hz), 2.23 (7 H, ddd, *J =* 25.7, 15.5, 8.3 Hz, H2, H4, H10, H13), 1.97 (1 H, dd, *J =* 16.1, 8.6 Hz, 11-OH), 1.75 (3 H, d, *J =* 14.7 Hz, H6, H8), 1.66–1.57 (4 H, m, H1, H7, H13), 1.36 (3 H, s, H15), 1.32 (4 H, m), 1.27 (2 H, d, *J =* 13.0 Hz, H8), 1.10 (3 H, s, H18), 0.90 (3 H, m) 0.89 (3 H, d, *J =* 6.9 Hz, H17), 0.72 (3 H, d, *J =* 7.0 Hz, H16). ^13^C NMR (101 MHz, Chloroform-*d*) δ 216.89 (C3), 169.24 (C21), 139.06 (C19), 117.19 (C20), 74.54 (C11), 70.56 (C14), 58.00 (C4), 53.42 (C22), 51.72, 49.34, 45.39 (C9), 44.60 (C13), 43.99 (C12), 41.73 (C5), 36.55 (C6), 35.97 (C2), 34.48, 32.12, 30.31 (C8), 26.81 (C7), 26.37 (C18), 24.78 (C1),16.53 (C16), 14.77 (C15), 11.53 (C17). HR-MS (ESI): Calcd for C_26_H_43_NO_4_ (M + H^+^): 434.3270; Found: 434.3257.

#### 4.2.17. 22-Octylamineyl-22-deoxypleuromutilin (**22**)

White powder; yield: 62%; mp: 76–77 °C; ^1^H NMR (400 MHz, Chloroform-*d*) δ 6.53 (1 H, dd, *J =* 17.4, 11.0 Hz, H19), 5.80 (1 H, d, *J =* 8.5 Hz, H14), 5.36 (2 H, s, H20), 5.21 (1 H, d, *J =* 17.3, 6.5 Hz, H20), 3.78 (2 H, m, H22, H11), 3.37 (1 H, dd, *J* = 10.1, 6.5 Hz), 2.23 (7 H, ddd, *J =* 25.7, 15.5, 8.3 Hz, H2, H4, H10, H13), 1.97 (1 H, dd, *J =* 16.1, 8.6 Hz, 11-OH), 1.75 (3 H, d, *J =* 14.7 Hz, H6, H8), 1.66–1.57 (4 H, m, H1, H7, H13), 1.36 (3 H, s, H15), 1.32 (12 H, m), 1.27 (2 H, d, *J =* 13.0 Hz, H8), 1.10 (3 H, s, H18), 0.90 (3 H, m) 0.89 (3 H, d, *J =* 6.9 Hz, H17), 0.72 (3 H, d, *J =* 7.0 Hz, H16). ^13^C NMR (101 MHz, Chloroform-*d*) δ 216.89 (C3), 169.24 (C21), 139.06 (C19), 117.19 (C20), 74.54 (C11), 70.56 (C14), 58.00 (C4), 53.42 (C22), 51.72, 49.34, 45.39 (C9), 44.60 (C13), 43.99 (C12), 41.73 (C5), 36.55 (C6), 35.97 (C2), 34.48, 32.12, 30.31 (C8), 29.99, 29.48, 29.24, 27.23, 26.81 (C7), 26.37 (C18), 24.78 (C1),16.53 (C16), 14.77 (C15), 11.53 (C17). HR-MS (ESI): Calcd for C_30_H_51_NO_4_ (M + H^+^): 490.3896; Found: 490.3882.

#### 4.2.18. 22-Dodecylamineyl-22-deoxypleuromutilin (**23**)

White powder; yield: 46%; mp: 75–77 °C; ^1^H NMR (400 MHz, Chloroform-*d*) δ 6.53 (1 H, dd, *J =* 17.4, 11.0 Hz, H19), 5.80 (1 H, d, *J =* 8.5 Hz, H14), 5.36 (2 H, s, H20), 5.21 (1 H, d, *J =* 17.3, 6.5 Hz, H20), 3.78 (2 H, m, H22, H11), 3.37 (1 H, dd, *J* = 10.1, 6.5 Hz), 2.23 (7 H, ddd, *J =* 25.7, 15.5, 8.3 Hz, H2, H4, H10, H13), 1.97 (1 H, dd, *J =* 16.1, 8.6 Hz, 11-OH), 1.75 (3 H, d, *J =* 14.7 Hz, H6, H8), 1.66–1.57 (4 H, m, H1, H7, H13), 1.36 (3 H, s, H15), 1.32 (20 H, m), 1.27 (2 H, d, *J =* 13.0 Hz, H8), 1.10 (3 H, s, H18), 0.90 (3 H, m) 0.89 (3 H, d, *J =* 6.9 Hz, H17), 0.72 (3 H, d, *J =* 7.0 Hz, H16). ^13^C NMR (101 MHz, Chloroform-*d*) δ 216.89 (C3), 169.24 (C21), 139.06 (C19), 117.19 (C20), 74.54 (C11), 70.56 (C14), 58.00 (C4), 53.42 (C22), 51.72, 49.34, 45.39 (C9), 44.60 (C13), 43.99 (C12), 41.73 (C5), 36.55 (C6), 35.97 (C2), 34.48, 32.12, 30.31 (C8), 29.99, 29.95, 29.64, 29.52 29.48, 29.36, 29.24, 27.23, 26.81 (C7), 26.37 (C18), 24.78 (C1),16.53 (C16), 14.77 (C15), 11.53 (C17). HR-MS (ESI): Calcd for C_34_H_59_NO_4_ (M + H^+^): 546.4522; Found: 546.4506.

#### 4.2.19. 22-Hexadecylamineyl-22-deoxypleuromutilin (**24**)

White powder; yield: 41%; mp: 76–79 °C; ^1^H NMR (400 MHz, Chloroform-*d*) δ 6.53 (1 H, dd, *J =* 17.4, 11.0 Hz, H19), 5.80 (1 H, d, *J =* 8.5 Hz, H14), 5.36 (2 H, s, H20), 5.21 (1 H, d, *J =* 17.3, 6.5 Hz, H20), 3.78 (2 H, m, H22, H11), 3.37 (1 H, dd, *J* = 10.1, 6.5 Hz), 2.23 (7 H, ddd, *J =* 25.7, 15.5, 8.3 Hz, H2, H4, H10, H13), 1.97 (1 H, dd, *J =* 16.1, 8.6 Hz, 11-OH), 1.75 (3 H, d, *J =* 14.7 Hz, H6, H8), 1.66–1.57 (4 H, m, H1, H7, H13), 1.36 (3 H, s, H15), 1.32 (28 H, m), 1.27 (2 H, d, *J =* 13.0 Hz, H8), 1.10 (3 H, s, H18), 0.90 (3 H, m) 0.89 (3 H, d, *J =* 6.9 Hz, H17), 0.72 (3 H, d, *J =* 7.0 Hz, H16). ^13^C NMR (101 MHz, Chloroform-*d*) δ 216.89 (C3), 169.24 (C21), 139.06 (C19), 117.19 (C20), 74.54 (C11), 70.56 (C14), 58.00 (C4), 53.42 (C22), 51.72, 49.34, 45.39 (C9), 44.60 (C13), 43.99 (C12), 41.73 (C5), 36.55 (C6), 35.97 (C2), 34.48, 32.12, 30.31 (C8), 29.99, 29.95, 29.70, 29.64, 29.62, 29.58, 29.52 29.48, 29.37, 29.36, 29.24, 27.23, 26.81 (C7), 26.37 (C18), 24.78 (C1),16.53 (C16), 14.77 (C15), 11.53 (C17). HR-MS (ESI): Calcd for C_38_H_67_NO_4_ (M + H^+^): 602.5148; Found: 602.5128.

#### 4.2.20. 22-(2-(Methylsulfonyl)ethylamineyl)-22-deoxypleuromutilin (**25**)

White powder; yield: 80%; mp: 183–186 °C; ^1^H NMR (400 MHz, Chloroform-*d*) δ 6.50 (1 H, dd, *J =* 17.4, 11.0 Hz, H19), 5.82 (1 H, d, *J =* 8.5 Hz, H14), 5.34 (2 H, s, H20), 5.20 (1 H, d, *J =* 17.3 Hz, H20), 3.78 (2 H, m, H22, H11), 3.37 (1 H, dd, *J* = 10.1, 6.5 Hz), 3.22–3.07 (4 H, m), 3.01 (3 H, s), 2.23 (5 H, dd, *J =* 25.7, 15.5, 8.3 Hz, H2, H4, H10, H13), 1.97 (1 H, dd, *J =* 16.1, 8.6 Hz, 11-OH), 1.75 (3 H, d, *J =* 14.7 Hz, H6, H8), 1.66–1.57 (4 H, m, H1, H7, H13), 1.36 (3 H, s, H15), 1.27 (2 H, d, *J =* 13.0 Hz, H8), 1.10 (3 H, s, H18), 0.89 (3 H, d, *J =* 6.9 Hz, H17), 0.72 (3 H, d, *J =* 7.0 Hz, H16). ^13^C NMR (101 MHz, Chloroform-*d*) δ 216.89 (C3), 169.24 (C21), 139.06 (C19), 117.19 (C20), 77.23, 77.02, 76.81, 74.54 (C11), 70.56 (C14), 58.00 (C4), 53.42 (C22), 45.39 (C9), 44.60 (C13), 43.99 (C12), 41.73 (C5), 36.55 (C6), 35.97 (C2), 30.31 (C8), 26.81 (C7), 26.37 (C18), 24.78 (C1),16.53 (C16), 14.77 (C15), 11.53 (C17). HR-MS (ESI): Calcd for C_25_H_41_NO_6_S (M + H^+^): 484.2733; Found: 484.2718.

#### 4.2.21. 22-(2-(Methylthio)ethylamineyl)-22-deoxypleuromutilin (**26**)

White powder; yield: 82%; mp: 153–155 °C; ^1^H NMR (400 MHz, Chloroform-*d*) δ 6.53 (1 H, dd, *J =* 17.4, 11.0 Hz, H19), 5.80 (1 H, d, *J =* 8.5 Hz, H14), 5.35 (2 H, s, H20), 5.20 (1 H, d, *J =* 17.3 Hz, H20), 3.78 (2 H, m, H22, H11), 3.37 (1 H, dd, *J* = 10.1, 6.5 Hz), 3.22–3.07 (4 H, m), 3.01 (3 H, s), 2.23 (5 H, ddd, *J =* 25.7, 15.5, 8.3 Hz, H2, H4, H10, H13), 1.97 (1 H, dd, *J =* 16.1, 8.6 Hz, 11-OH), 1.75 (3 H, d, *J =* 14.7 Hz, H6, H8), 1.66–1.57 (4 H, m, H1, H7, H13), 1.36 (3 H, s, H15), 1.27 (2 H, d, *J =* 13.0 Hz, H8), 1.10 (3 H, s, H18), 0.89 (3 H, d, *J =* 6.9 Hz, H17), 0.72 (3 H, d, *J =* 7.0 Hz, H16). ^13^C NMR (101 MHz, Chloroform-*d*) δ 216.89 (C3), 169.24 (C21), 139.06 (C19), 117.19 (C20), 77.23, 77.02, 76.81, 74.54 (C11), 70.56 (C14), 58.00 (C4), 53.42 (C22), 45.39 (C9), 44.60 (C13), 43.99 (C12), 41.73 (C5), 36.55 (C6), 35.97 (C2), 30.31 (C8), 26.81 (C7), 26.37 (C18), 24.78 (C1),16.53 (C16), 14.77 (C15), 11.53 (C17). HR-MS (ESI): Calcd for C_25_H_41_NO_4_S (M + H^+^): 452.2834; Found: 452.2820.

#### 4.2.22. 22-Amantadineyl-22-deoxypleuromutilin (**27**)

White powder; yield: 62%; mp: 105–109 °C; ^1^H NMR (400 MHz, Chloroform-*d*) δ 6.53 (1 H, dd, *J =* 17.4, 11.0 Hz, H19), 5.80 (1 H, d, *J =* 8.5 Hz, H14), 5.36 (2 H, s, H20), 5.21 (1 H, d, *J =* 17.3, 6.5 Hz, H20), 3.78 (2 H, m, H22, H11), 3.37 (1 H, dd, *J* = 10.1, 6.5 Hz), 2.23 (7 H, ddd, *J =* 25.7, 15.5, 8.3 Hz, H2, H4, H10, H13), 1.97 (1 H, dd, *J =* 16.1, 8.6 Hz, 11-OH), 1.75 (3 H, d, *J =* 14.7 Hz, H6, H8), 1.66–1.57 (4 H, m, H1, H7, H13), 1.36 (3 H, s, H15), 1.32 (12 H, m), 1.27 (2 H, d, *J =* 13.0 Hz, H8), 1.10 (3 H, s, H18), 0.90 (3 H, m) 0.89 (3 H, d, *J =* 6.9 Hz, H17), 0.72 (3 H, d, *J =* 7.0 Hz, H16). ^13^C NMR (101 MHz, Chloroform-*d*) δ 216.89 (C3), 169.24 (C21), 139.06 (C19), 117.19 (C20), 74.54 (C11), 70.56 (C14), 58.00 (C4), 53.42 (C22), 45.39 (C9), 44.60 (C13), 43.99 (C12), 42.50, 41.73 (C5), 36.74, 36.55 (C6), 36.27, 35.97 (C2), 34.49, 30.31 (C8), 26.81 (C7), 26.37 (C18), 24.78 (C1),16.53 (C16), 14.77 (C15), 11.53 (C17). HR-MS (ESI): Calcd for C_32_H_49_NO_4_ (M + H^+^): 512.3740; Found: 512.3728.

#### 4.2.23. 22-Ethanolamine-22-deoxypleuromutilin (**28**)

White powder; yield: 84%; mp: 91–94 °C; ^1^H NMR (400 MHz, Chloroform-*d*) δ 6.54 (1 H, dd, *J =* 17.4, 11.0 Hz, H19), 5.80 (1 H, d, *J =* 8.5 Hz, H14), 5.35 (2 H, s, H20), 5.20 (1 H, d, *J =* 17.3 Hz, H20), 3.78 (2 H, m, H22, H11), 3.37 (1 H, dd, *J* = 10.1, 6.5 Hz), 3.22–3.07 (2 H, m), 2.80 (2 H, m), 2.23 (5 H, ddd, *J =* 25.7, 15.5, 8.3 Hz, H2, H4, H10, H13), 1.97 (1 H, dd, *J =* 16.1, 8.6 Hz, 11-OH), 1.75 (3 H, d, *J =* 14.7 Hz, H6, H8), 1.66–1.57 (4 H, m, H1, H7, H13), 1.36 (3 H, s, H15), 1.27 (2 H, d, *J =* 13.0 Hz, H8), 1.10 (3 H, s, H18), 0.89 (3 H, d, *J =* 6.9 Hz, H17), 0.72 (3 H, d, *J =* 7.0 Hz, H16). ^13^C NMR (101 MHz, Chloroform-*d*) δ 216.89 (C3), 169.24 (C21), 139.06 (C19), 117.19 (C20), 74.54 (C11), 70.56 (C14), 68.79, 60.92, 58.00 (C4), 53.42 (C22), 45.39 (C9), 44.60 (C13), 43.99 (C12), 41.73 (C5), 36.55 (C6), 35.97 (C2), 30.31 (C8), 26.81 (C7), 26.37 (C18), 24.78 (C1),16.53 (C16), 14.77 (C15), 11.53 (C17). HR-MS (ESI): Calcd for C_24_H_39_NO_5_ (M + H^+^): 422.2906; Found: 422.2892.

#### 4.2.24. 22-Morpholine-22-deoxypleuromutilin (**29**)

White powder; yield: 67%; mp: 157–159 °C; ^1^H NMR (400 MHz, Chloroform-*d*) δ 6.52 (1 H, dd, *J =* 17.4, 11.0 Hz, H19), 5.80 (1 H, d, *J =* 8.5 Hz, H14), 5.34 (2 H, s, H20), 5.20 (1 H, d, *J =* 17.3 Hz, H20), 3.78 (2 H, m, H22, H11), 2.23 (5 H, ddd, *J =* 25.7, 15.5, 8.3 Hz, H2, H4, H10, H13), 2.11 (2 H, m), 1.97 (1 H, dd, *J =* 16.1, 8.6 Hz, 11-OH), 1.75 (3 H, d, *J =* 14.7 Hz, H6, H8), 1.73 (2 H, m), 1.66–1.57 (4 H, m, H1, H7, H13), 1.36 (3 H, s, H15), 1.27 (2 H, d, *J =* 13.0 Hz, H8), 1.10 (3 H, s, H18), 0.89 (3 H, d, *J =* 6.9 Hz, H17), 0.72 (3 H, d, *J =* 7.0 Hz, H16). ^13^C NMR (101 MHz, Chloroform-*d*) δ 216.89 (C3), 169.24 (C21), 139.06 (C19), 117.19 (C20), 74.54 (C11), 70.56 (C14), 68.29, 60.74, 58.00 (C4), 53.42 (C22), 45.39 (C9), 44.60 (C13), 43.99 (C12), 41.73 (C5), 36.55 (C6), 35.97 (C2), 30.31 (C8), 26.81 (C7), 26.37 (C18), 24.78 (C1),16.53 (C16), 14.77 (C15), 11.53 (C17). HR-MS (ESI): Calcd for C_26_H_41_NO_5_ (M + H^+^): 448.3063; Found: 448.3049.

#### 4.2.25. 22-Thiomorpholine-22-deoxypleuromutilin (**30**)

White powder; yield: 59%; mp: 82–83 °C; ^1^H NMR (400 MHz, Chloroform-*d*) δ 6.52 (1 H, dd, *J =* 17.4, 11.0 Hz, H19), 5.80 (1 H, d, *J =* 8.5 Hz, H14), 5.34 (2 H, s, H20), 5.20 (1 H, d, *J =* 17.3 Hz, H20), 3.78 (2 H, m, H22, H11), 2.23 (5 H, ddd, *J =* 25.7, 15.5, 8.3 Hz, H2, H4, H10, H13), 2.12 (2 H, m), 1.97 (1 H, dd, *J =* 16.1, 8.6 Hz, 11-OH), 1.75 (3 H, d, *J =* 14.7 Hz, H6, H8), 1.67 (2 H, m), 1.66–1.57 (4 H, m, H1, H7, H13), 1.36 (3 H, s, H15), 1.27 (2 H, d, *J =* 13.0 Hz, H8), 1.10 (3 H, s, H18), 0.89 (3 H, d, *J =* 6.9 Hz, H17), 0.72 (3 H, d, *J =* 7.0 Hz, H16). ^13^C NMR (101 MHz, Chloroform-*d*) δ 216.89 (C3), 169.24 (C21), 139.06 (C19), 117.19 (C20), 74.54 (C11), 70.56 (C14), 68.29, 60.80, 58.00 (C4), 53.42 (C22), 45.39 (C9), 44.60 (C13), 43.99 (C12), 41.73 (C5), 36.55 (C6), 35.97 (C2), 30.31 (C8), 26.81 (C7), 26.37 (C18), 24.78 (C1),16.53 (C16), 14.77 (C15), 11.53 (C17). HR-MS (ESI): Calcd for C_26_H_41_NO_4_S (M + H^+^): 464.2834; Found: 464.2821.

### 4.3. In Vitro Efficacy of Pleuromutilin Derivatives

#### 4.3.1. Minimal Inhibitory Concentration (MIC) and Minimum Bactericidal Concentration (MBC) Testing

The minimal inhibitory concentration (MIC) of the target pleuromutilin derivatives against methicillin-resistant *S*. *aureus* (ATCC 43300), *S*. *aureus* (ATCC 29213), *S*. *aureus* (AD3) and *S*. *aureus* (144) were determined by using tiamulin as the positive control. MIC values were tested following our previous work [20]. The derivatives were dissolved in an aqueous solution of 2.5% tween-80 and 2.5% DMSO to make a solution with a concentration of 1000 µg/mL. The solution was diluted 12 times with MH broth to give a concentration range of 32–0.01 µg/mL. The bacteria (5 × 10^5^ CFU/mL) were added in each well. For each compound concentration, three parallel experiments were set. The 96-well plate was incubated for 22–26 h at 37 °C. The MIC value was recorded as the minimal concentration that inhibits the visible growth of tested bacteria completely.

The MBC of these pleuromutilin derivatives were tested according to a previously reference [20]. After recording the MIC value, the 96-well plate was placed for 22–26 h at 37 °C. Then, the values of MBC were determined by laying 100 μL aliquots from the wells onto the MH agar plates without visible growth. The MH agar plate was cultured for a further 22–26 h at 37 °C. The minimal concentration of the derivative was MBC, required to decrease the bacterial count on the plate by more than 99.9%.

#### 4.3.2. Constant Concentration Time-Kill Curves

The time-kill curve experiments of compound **8** and compound **9** were performed according to a previous report in triplicate. The logarithmic phase of MRSA 43300 was diluted to about 10^6^ CFU/mL with MH broth. Compound **8** and compound **9** with a final concentration of 1× MIC, 2× MIC, 4× MIC, 8× MIC, 16× MIC, and 32× MIC were added into the bacteria solution. All samples were shaken at 37 °C. Samples (100 μL) were taken at 0, 3, 6, 9 and 24 h from the mixtures. Each sample was diluted decuple continuously with sterile saline. Then, 20 μL of the diluted sample was plated on the sterile MH agar plate and incubated for 22–26 h at 37 °C. The total bacteria on the plates were counted as CFU/mL to calculate the bacterial colonies. The time-kill curve was constructed by scheming log_10_ CFU/mL of bacteria counts versus time.

#### 4.3.3. Determination of the Post Antibiotic Effect (PAE)

The post antibiotic effect (PAE) against MRSA of compound **9** were determined using time-kill methods according to previous reports [41]. In this experiment, MRSA 43300 in logarithmic phase was diluted with MH broth to about 10^6^ CFU/mL. Compound **9** was added to the tubes at the concentrations of 2× MIC and 4× MIC containing the inoculum. Then, the samples were shaken at 37 °C for 2 h after the incubation was completed. By diluting with the preheated MH broth 1000 times, the compound was removed from the sample. The tubes were placed in 37 °C and the samples (100 μL) was extracted at time 0, 1, 2, 4, 6 and 8 h. The samples were diluted decuple with sterile saline and plated onto the preheated MH agar plates. The plates were counted after 22–26 h of incubation in 37 °C. The PAE value was calculated by the equation (PAE = TA – TC) and presented after an hour. (TA is the time required for the bacteria in the test to increase by 1 log_10_ CFU/mL, and TC is the time required for control groups to increase by 1 log_10_ CFU/mL).

### 4.4. S. aureus Growth and Ribosome Purification

*S. aureus* ATCC 43300 was used to prepare the 50S ribosomal subunit the same way as described previously [41]. Cells were grown overnight in Brain Heart Infusion broth at 37 °C and harvested for 1.5 h at an OD600. Cells were washed with 10 mM Tris-HCl twice at pH = 7.5, and resuspended in 5 mL buffer A (20 mM HEPES-HCl pH = 7.5, 21 mM Mg(OAc)_2_, 100 mM NH_4_Cl, 1 mM DTT, 1 mM EDTA). Cells were broken by an ultrasound crusher. The lysate was centrifuged for 90 min at 20,000× *g* to clear cell debris.

The solution was layered on a sucrose cushion of 15 mL (10 mM Hepes-KOH pH 7.5, 25 mM Mg(OAc)_2_, 500 mM KCl, 1.1 mM Sucrose, 1 mM DTT, 0.5 mM EDTA). Centrifugation was carried out for 15 h at 45,000 rpm using an ultracentrifuge Beckman (Beckman Coulter, Shanghai, China), Type 70Ti rotor. The crude ribosome pellet was resuspended in buffer E (10 mM Hepes-KOH pH 7.5, 10 mM Mg(OAc)_2_, 100 mM KCl, 1 mM DTT, 0.5 mM EDTA), then layered on 9 mL of 7–30% sucrose gradient and centrifuged in an ultracentrifuge Beckman, type SW40Ti rotor at 17,100 rpm for 15 h. The gradient was analyzed on the AKTA explorer system (Amersham, UK). The fractions corresponding to the 50S ribosomal subunit were collected and further subjected to precipitation by PEG 20,000. The 50S ribosomal subunit pellet was gently dissolved in 200 µL of storage buffer (10 mM HEPES-HCl pH = 7.5, 10 mM Mg(OAc)_2_, 15 mM KCl, 60 mM NH_4_Cl, 1 mM DTT) and stored at −80 °C.

### 4.5. SPR Interaction and Affinity Analysis

SPR affinity analysis was performed on the bScreen LB 991 Label-free Microarray System (Berthold Technologies, Black Forest, Germany) at 4 °C and Biodot AD-1520 Array Printer (BIODOT Inc., Irvine, CA, USA) with a Photo-cross-linker SensorCHIP™. Concentrations of each compound were diluted to 100 μM with DMSO as the printing working solution for immobilization. A Biodot AD-1520 Array Printer (BIODOT Inc., CA, USA) was used to control the photo-cross-linker sensor chip and print samples. Each sample was printed four times. In order to check the chip quality and check whether the detection system works, DMSO was used as a system-negative control and rapamycin as a positive control. All positive control dots were divided into four groups and printed on the four corners of the sensor chip. The sensor chip was dried in vacuum after the array print and quickly transferred to a spectra radiator for a photo cross-linking reaction. The sensor chip was washed with C_2_H_5_OH, DMF and H_2_O in turn and evaporated in a N_2_ atmosphere for 15 min. Then, the Flowcell Cover were assembled.

The 50S ribosome was diluted separately to 200 nM, 400 nM, 800 nM, 1600 nM and 3200 nM with PBST (pH = 7.4, 0.1% Tween 20). Different concentrations of ribosome solutions were injected at a flow rate of 0.5 μL⋅s^−1^ for 600 s at the associating stage at 4 °C, followed by PBST at a flow rate of 0.5⋅μL·s^−1^ for 360 s at each dissociating stage. Then, the surface was regenerated with Glycine-HCl (pH = 2.0) at a flow rate of 2⋅μL·s^−1^ for 300 s. To validate detection of the compound–protein interactions, we arranged tiamulin as the positive control and DMSO and penicillin as the negative controls and designed kinetic constant tests after the sample tests with FKBP12.

The raw data of the binding process of compounds and the 50S ribosome were recorded in real time. We compared the response unit (RU) of surface resonance to determine the different binding affinity between each sample dot. The process and analysis of association rate constants (K_a_) and dissociation rate constants (K_d_) and the equilibrium dissociation constant (K_D_, K_d_/K_a_) were performed using the data analysis software of the bScreen LB 991 Label-free Microarray System according to a single-site binding model (1:1 Langmuir binding) with mass transfer limitations for binding kinetics determination.

### 4.6. Molecular Modeling

In order to reveal the binding modes of synthesized pleuromutilin analogues, docking was performed based on the crystal structure of *S. aureus* 50S ribosomal in complex with tiamulin (PDB ID code: 1XBP). The peptidyl transferase center (PTC) model was built that consists of all residues within 40 Å around the tiamulin in 1XBP. The binding site of tiamulin in 1XBP was set to the docking position. All compounds were prepared with Avogadro 1.1.1. Docking experiments were performed using the AutoDock Vina (V.1.2.0) (Scripps Research Institute, San Diego, CA, USA)and Pymol (V.2.3.2) (Schrodinger, New York, NY, USA) [42].

### 4.7. Neutropenic Murine Thigh Infection Model

The neutropenic murine thigh infection model experiment was performed as described in the literature. Six-week-old SPF-ICR female mice weighing 24–28 g were used for this study. The mice were injected with cyclophosphamide (Mead Johnson Pharmaceuticals, Evansville, Indiana—IN, USA) at a dose of 150 mg/kg 4 days and at a dose of 100 mg/kg 1 day before the experiment to reduce neutrophils and achieve immunosuppression (<0.1 × 10^9^/L). A total of 0.1 mL MH broth (MRSA concentration approximately 10^7^ CFU/mL) was injected into the posterior thigh of mice to establish the infection model. After the thighs of the mice were infected for 2 h, these mice were randomly divided into 3 groups (3 per group). The mice were intravenously injected with 0.9% saline, compound **9** (20 mg/kg) and tiamulin (20 mg/kg). A total of 24 h after the completion of the drug, the mice were euthanized and their thigh tissues were collected. Then, their thigh tissues were weighed and homogenized, respectively, in 3 mL of iced sterile saline. Tissue homogenate was serially diluted 10-fold and plated on MH agar. The resulting bacterial colonies were determined after incubation for 24 h.

The protocol for this study was reviewed and approved by the Institutional Animal Care and Use Committee of the South China Agricultural University.

### 4.8. Effect of Derivatives on Human Liver Microsomal CYP3A4 Enzyme Activity

The inhibitory effect of compound **9** on CYP3A4 was evaluated according to previous work [27]. The experiments were performed in 96-well plates with a final incubation volume of 100 µL per well. A total of 20 µL of compound **9** was added to 96-well plates, with final concentrations of 0.25, 0.5, 1, 5, 10, and 50 µM. Then, 40 µL of human liver microsomes (final concentration of human liver microsomes was 0.2 mg/mL) and 20 µL of probe substrates (final concentration of testosterone was 20 μmol/L) in 0.1 M Tris (pH = 7.4) were added. After preincubation at 37 °C for 5 min, the reaction started with the addition of 20 µL of NADPH (final concentration is 1 mmol/L). Then, the plate was incubated at 37 °C for 5 min, and then 100 μL acetonitrile (with loratadine used as an internal standard) was added to terminate the reaction. After the reactions were completed, the plates were centrifuged and the supernatants were analyzed for the loratadine and probe substrates testosterone by LC-MS/MS.

## Figures and Tables

**Table 1:** MIC and MBC (μg/mL) values of compounds **8**–**30**, tiamulin against *S. aureus* (ATCC 43300), *S. aureus* (ATCC 29213), *S. aureus* (AD3) and *S. aureus* (144).

Compound No.	R	MIC/MBC (μg/mL)			
		MRSA	*S.aureus*	*S.aureus*	*S.aureus*
		ATCC	ATCC 29213	AD3	144
		43300			
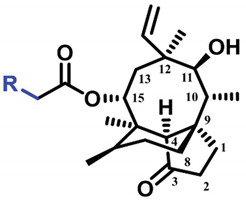					
**8**	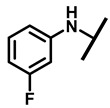	0.03/0.12	0.12/0.25	0.12/0.5	0.25/0.5
**9**	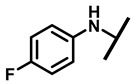	0.06/0.25	0.25/0.5	0.12/0.5	0.5/1
**10**	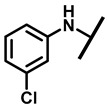	0.12/0.12	0.12/0.25	0.125/0.5	0.25/0.5
**11**	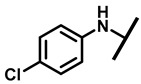	0.12/0.25	0.5/1	0.25/1	1/2
**12**	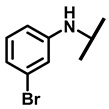	0.12/0.25	0.25/0.5	0.25/1	1/2
**13**	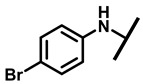	0.12/0.12	0.25/0.25	0.25/1	0.5/1
**14**	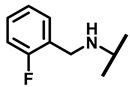	1/2	4/8	2/8	4/8
**15**	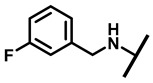	1/2	4/8	2/8	8/16
**16**	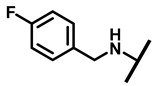	4/8	16/32	8/16	16/32
**17**	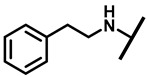	1/1	2/8	2/8	4/4
**18**	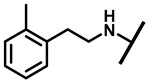	1/2	2/8	4/16	4/4
**19**	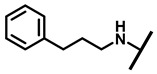	1/2	2/4	1/4	4/8
**20**	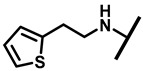	0.5/1	2/2	4/16	2/4
**21**	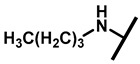	2/4	4/4	4/8	8/8
**22**	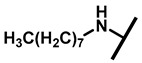	16/32	16/>32	32/>32	32/>32
**23**	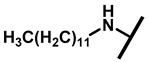	32/32	32/>32	32/>32	32/>32
**24**	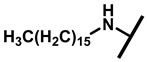	32/>32	32/>32	>32/>32	32/>32
**25**	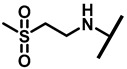	1/2	2/2	2/4	4/8
**26**	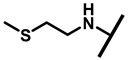	1/1	2/2	1/4	2/4
**27**	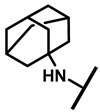	0.25/0.25	0.5/1	2/4	4/8
**28**	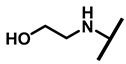	1/2	2/8	2/8	4/4
**29**	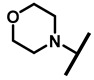	1/1	2/2	2/8	2/4
**30**	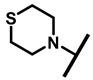	1/2	1/2	1/4	2/4
Tiamulin		0.25/0.25	0.5/1	1/1	1/8

**Table 2:** Binding affinities of compounds and controls between the 50S ribosome.

Compound No	Avg k_a_ (1/Ms)	Avg k_d_ (1/s)	Avg K_D_ (M)
Compound **8**	1.01 × 10^3^	3.56 × 10^−3^	3.52 × 10^−6^
Compound **9**	4.17 × 10^2^	7.39 × 10^−6^	1.77 × 10^−8^
Compound **10**	8.60 × 10	2.24 × 10^−3^	2.60 × 10^−5^
Compound **11**	3.67 × 10	1.90 × 10^−3^	5.18 × 10^−5^
Compound **12**	4.08 × 10	6.25 × 10^−5^	1.53 × 10^−6^
Compound **13**	3.10 × 10	1.97 × 10^−1^	6.35 × 10^−3^
Compound **14**	1.53 × 10^2^	6.50 × 10^−2^	4.24 × 10^−4^
Compound **15**	1.96 × 10^3^	4.32 × 10^−3^	2.21 × 10^−6^
Compound **16**	2.04	9.29 × 10^−3^	4.56 × 10^−3^
Compound **17**	2.05	9.63 × 10^−3^	4.69 × 10^−3^
Compound **18**	1.34 × 10^2^	4.45 × 10^−3^	3.32 × 10^−5^
Compound **19**	3.11 × 10^3^	4.69 × 10^−4^	1.51 × 10^−7^
Compound **20**	6.24 × 10^2^	1.97 × 10^−2^	3.16 × 10^−5^
Compound **21**	4.24	2.31 × 10^−2^	5.44 × 10^−3^
Compound **22**	1.14 × 10^2^	7.30 × 10^−4^	6.41 × 10^−6^
Compound **23**	2.41 × 10^4^	1.10 × 10^−3^	4.56 × 10^−8^
Compound **24**	3.77 × 10	3.75 × 10^−2^	9.93 × 10^−4^
Compound **25**	2.70	8.33 × 10^−3^	3.08 × 10^−3^
Compound **26**	1.26	7.79 × 10^−1^	6.19 × 10^−1^
Compound **27**	7.36	2.15 × 10^−2^	2.92 × 10^−3^
Compound **28**	2.73 × 10^3^	2.72 × 10^−2^	9.96 × 10^−6^
Compound **29**	1.57	8.72 × 10^−1^	5.55 × 10^−1^
Compound **30**	2.70 × 10	2.10 × 10^−4^	7.77 × 10^−6^
Tiamulin	1.21 × 10^2^	3.03 × 10^−6^	2.50 × 10^−8^
Penicillin	2.10	5.15 × 10^−1^	2.45 × 10^−1^
DMSO	2.43	5.80 × 10^−1^	2.39 × 10^−1^

## Supplementary Materials

The following are available online. The characterization spectrum of synthesized compounds.

## Sample Availability

Samples of the compounds **8**–**30** are available from the authors.
