# Supplementary material for: Discovery of Novel Pleuromutilin Derivatives as Potent Antibacterial Agents for the Treatment of MRSA Infection
Source: Molecules. 2022 Jan 29;27(3):931. doi: 10.3390/molecules27030931 (PMC8838415; doi:10.3390/molecules27030931)
Supplement: Supplementary file 1 [file molecules-27-00931-s001.zip › molecules-1554912-supplementary.pdf]

Discovery of Novel pleuromutilin derivatives as potent antibacterial agents for the treatment of MRSA infection

Han-Qing Fang[1], Jie Zeng[1], Shou-Kai Wang[1], Xiao Wang[1], Fang Chen[1], Bo Li[1], Jie Liu[1], Zhen Jin[1,2], Ya-Hong Liu[1,2], You-Zhi Tang[1,2]*

[1]*Guangdong Provincial Key Laboratory of Veterinary Pharmaceutics Development and Safety Evaluation, College of Veterinary Medicine, South China Agricultural University, Guangzhou 510642, China*

[2]*Guangdong Laboratory for Lingnan Modern Agriculture, Guangzhou, 510642, China*

Correspondence should be addressed:

College of Veterinary Medicine, South China Agricultural University, No. 483 Wushan Road, Tianhe District, Guangzhou 510642, China

You-Zhi Tang,

Fax: +86 20 85280665; Email: youzhitang@scau.edu.cn

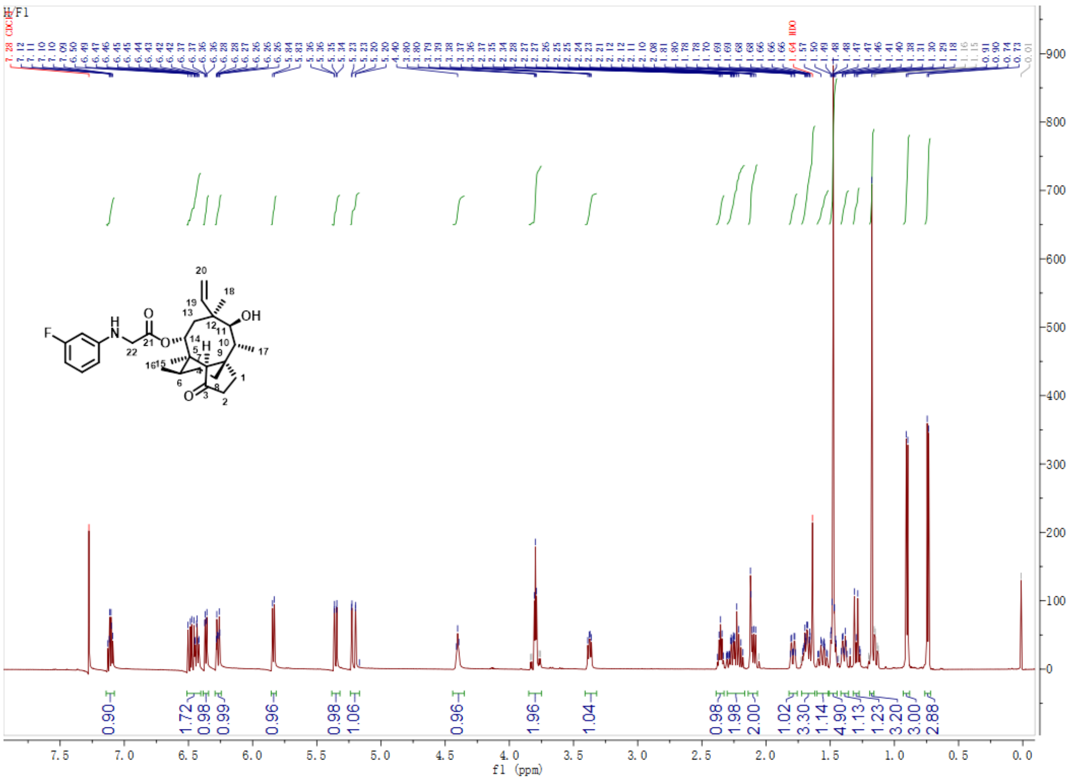

Figure SI 1-1. ^{1}H-NMR spectrum ($CDCl_3$, 400MHz) of compound **8**.

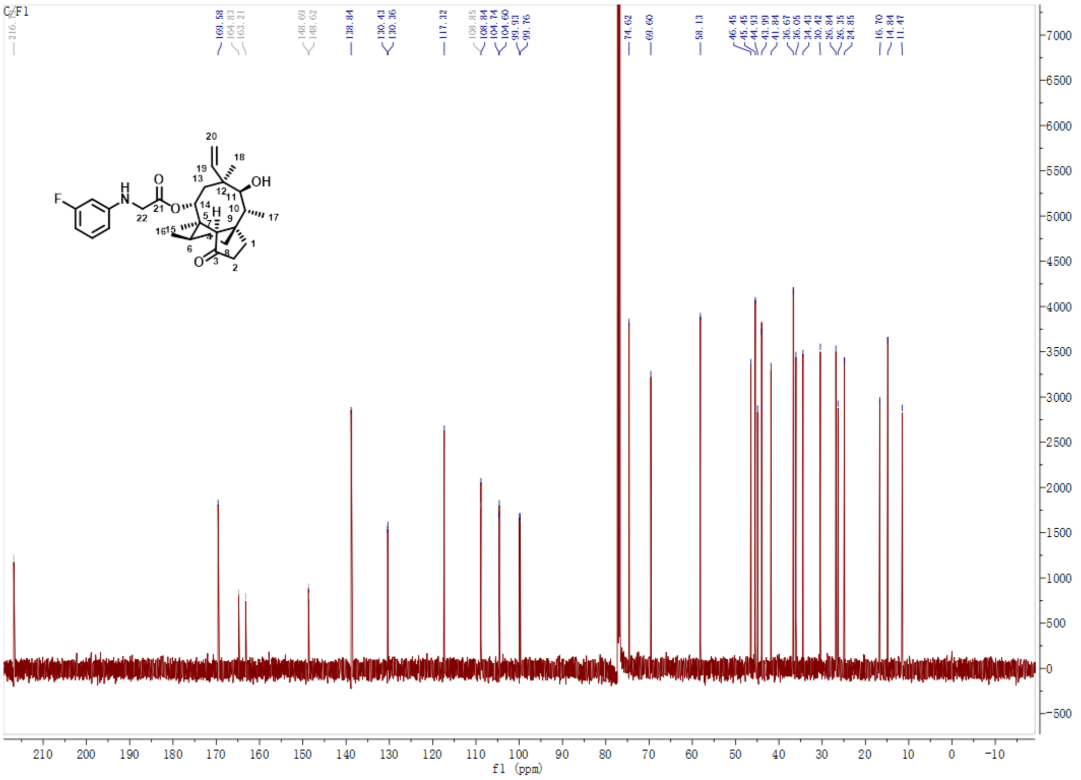

Figure SI 1-2. ^{13}C-NMR spectrum ($CDCl_3$, 101MHz) of compound **8**.

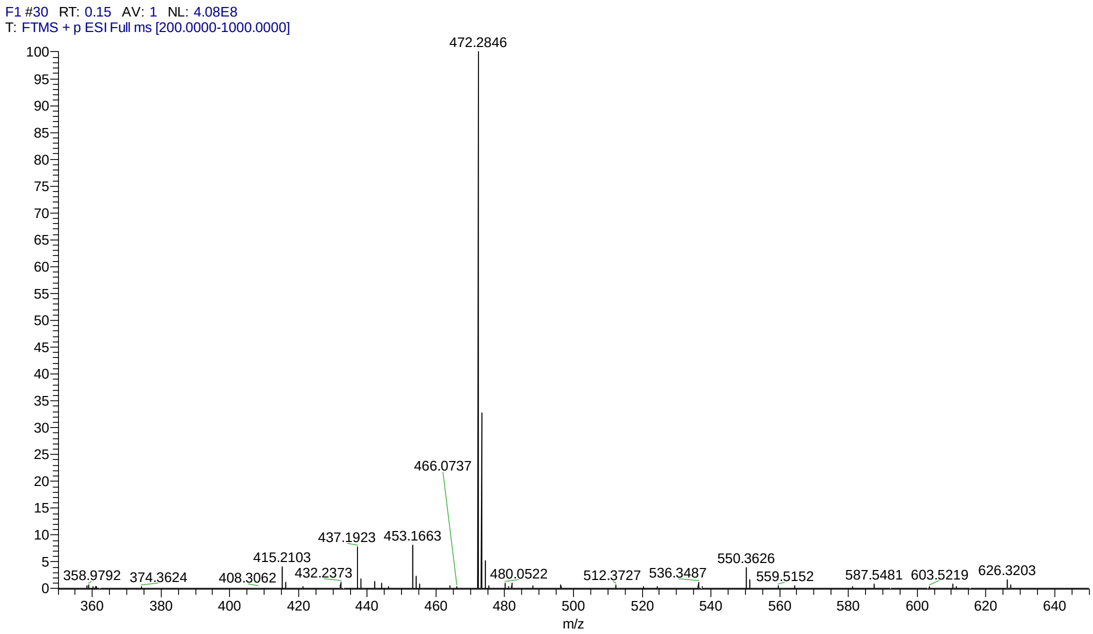

Figure SI 1-3. HR Mass spectrum (ESI) of compound **8**.

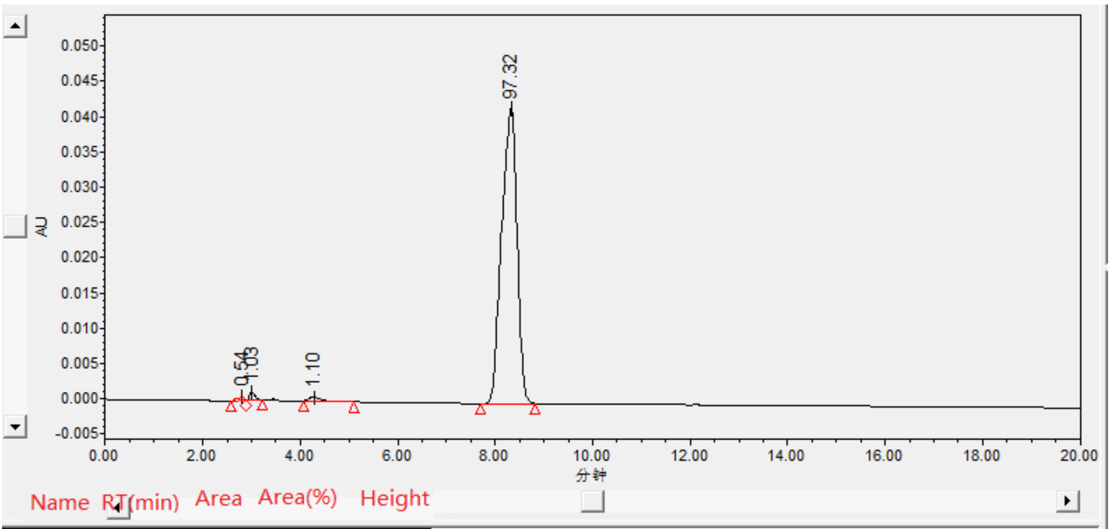

	名称	保留时间 (分钟)	面积 (微伏*秒)	% 面积	高度 (微伏)
1		2.803	5044	0.54	589
2		3.011	9679	1.03	1141
3		4.276	10344	1.10	570
4		8.323	911833	97.32	41865

Figure SI 1-4. HPLC of compound **8**.

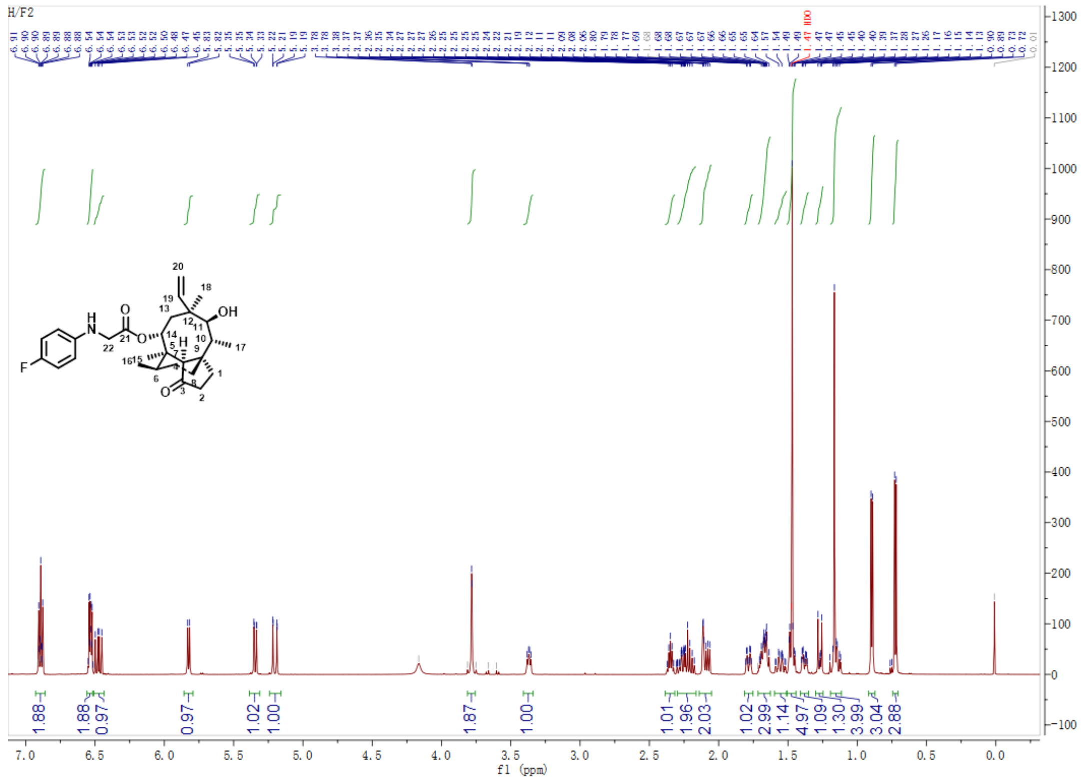

Figure SI 2-1. ^{1}H-NMR spectrum ($CDCl_3$,400MHz) of compound **9**.

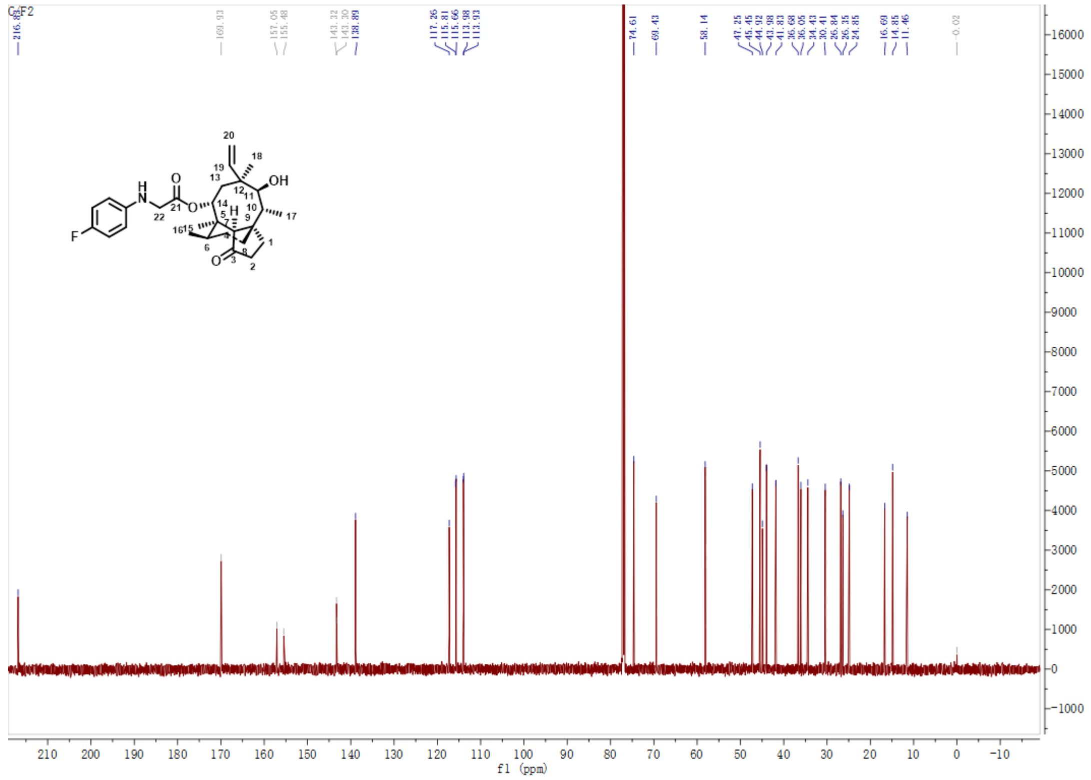

Figure SI 2-2. ^{13}C-NMR spectrum ($CDCl_3$, 101MHz) of compound **9**.

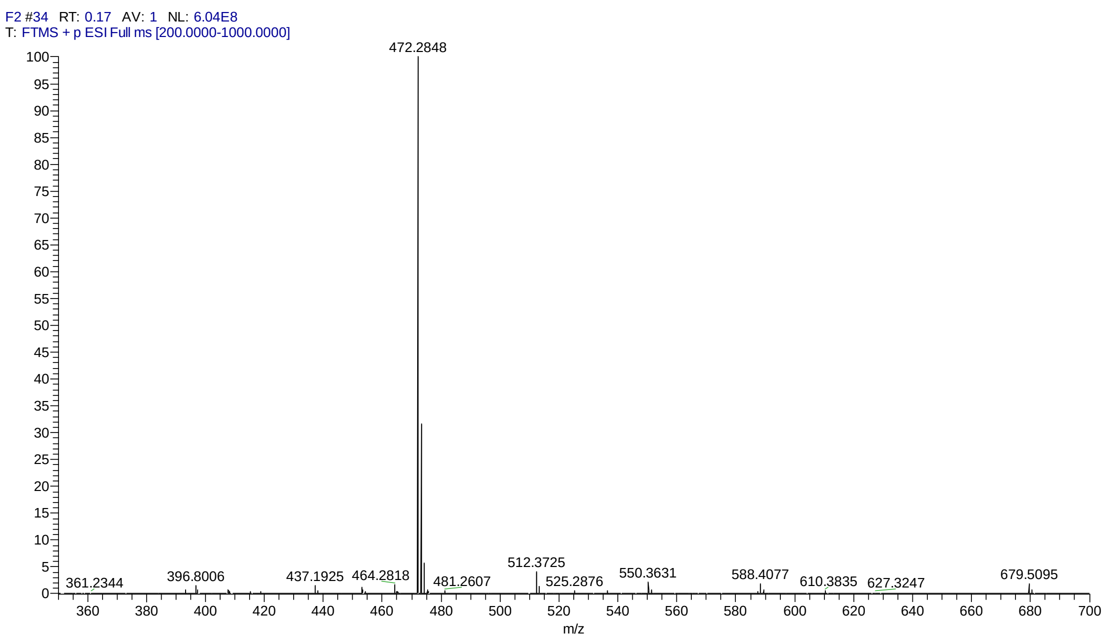

Figure SI 2-3. HR Mass spectrum (ESI) of compound **9**.

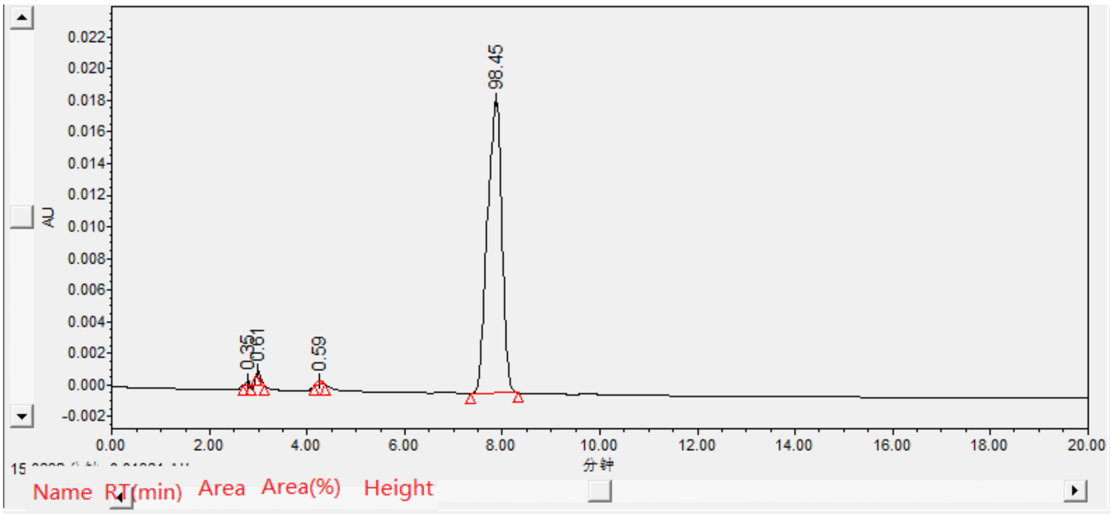

	名称	保留时间 (分钟)	面积 (微伏*秒)	% 面积	高度 (微伏)
1		2.784	1345	0.35	331
2		2.991	2370	0.61	441
3		4.252	2289	0.59	266
4		7.872	382254	98.45	18600

Figure SI 2-4. HPLC of compound **9**.

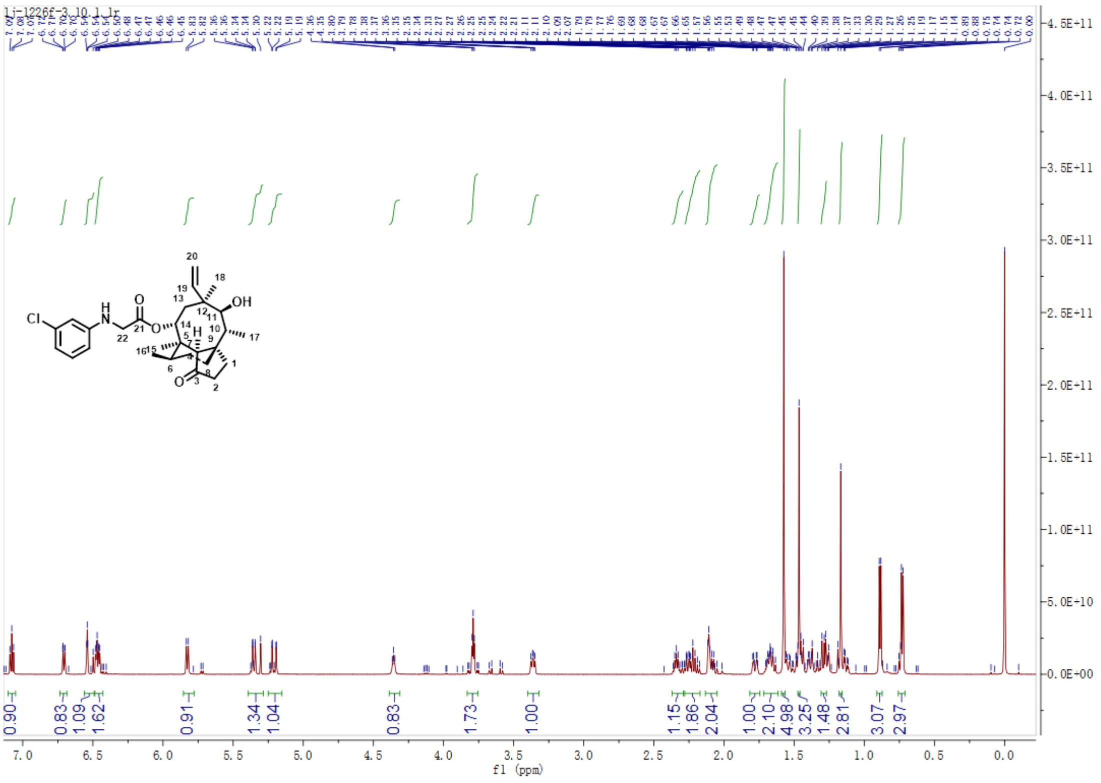

Figure SI 3-1. ^{1}H-NMR spectrum ($CDCl_3$, 400MHz) of compound **10**.

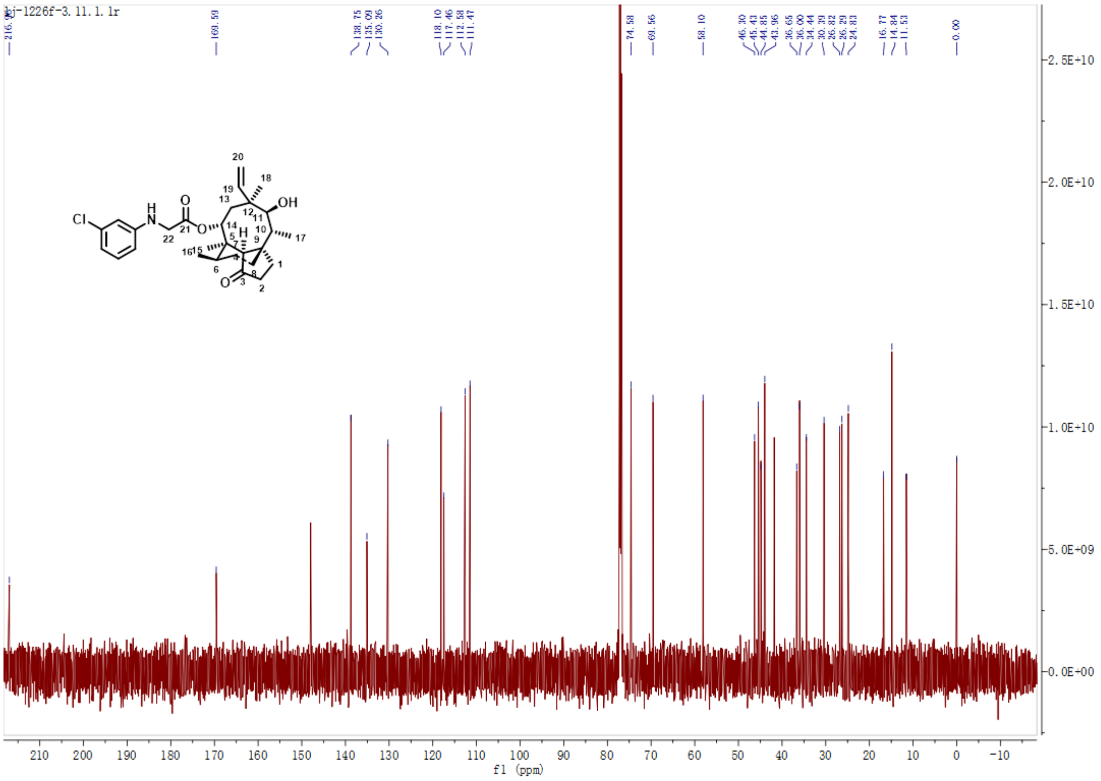

Figure SI 3-2. ^{13}C-NMR spectrum ($CDCl_3$, 101MHz) of compound **10**.

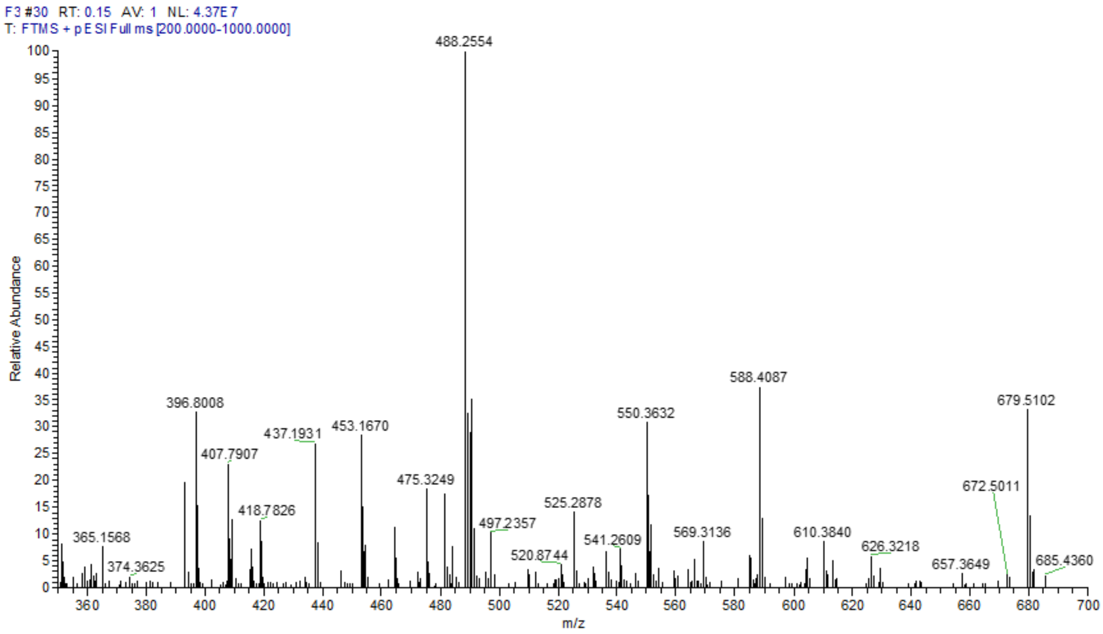

Figure SI 3-3. HR Mass spectrum (ESI) of compound **10**.

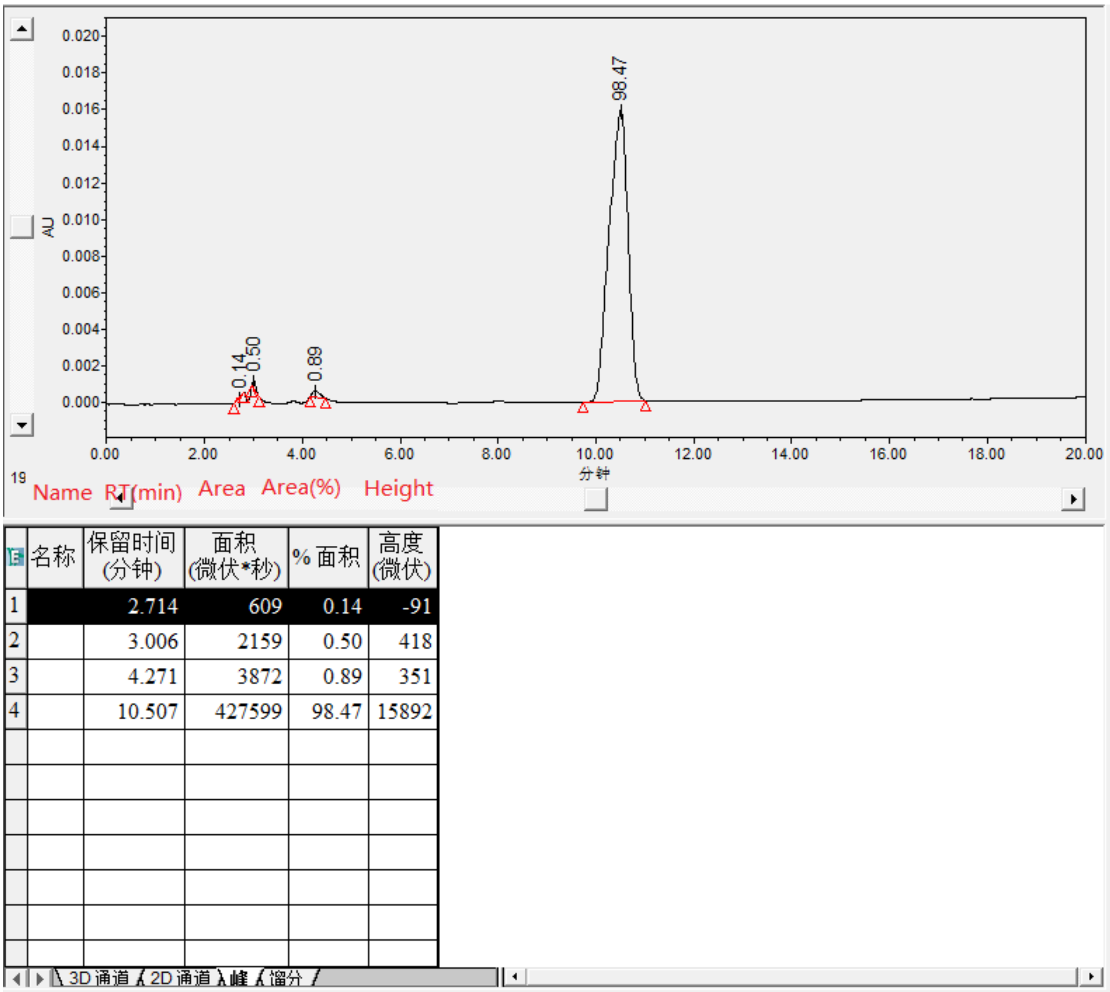

	名称	保留时间 (分钟)	面积 (微伏*秒)	% 面积	高度 (微伏)
1		2.714	609	0.14	-91
2		3.006	2159	0.50	418
3		4.271	3872	0.89	351
4		10.507	427599	98.47	15892

Figure SI 3-4. HPLC of compound **10.**

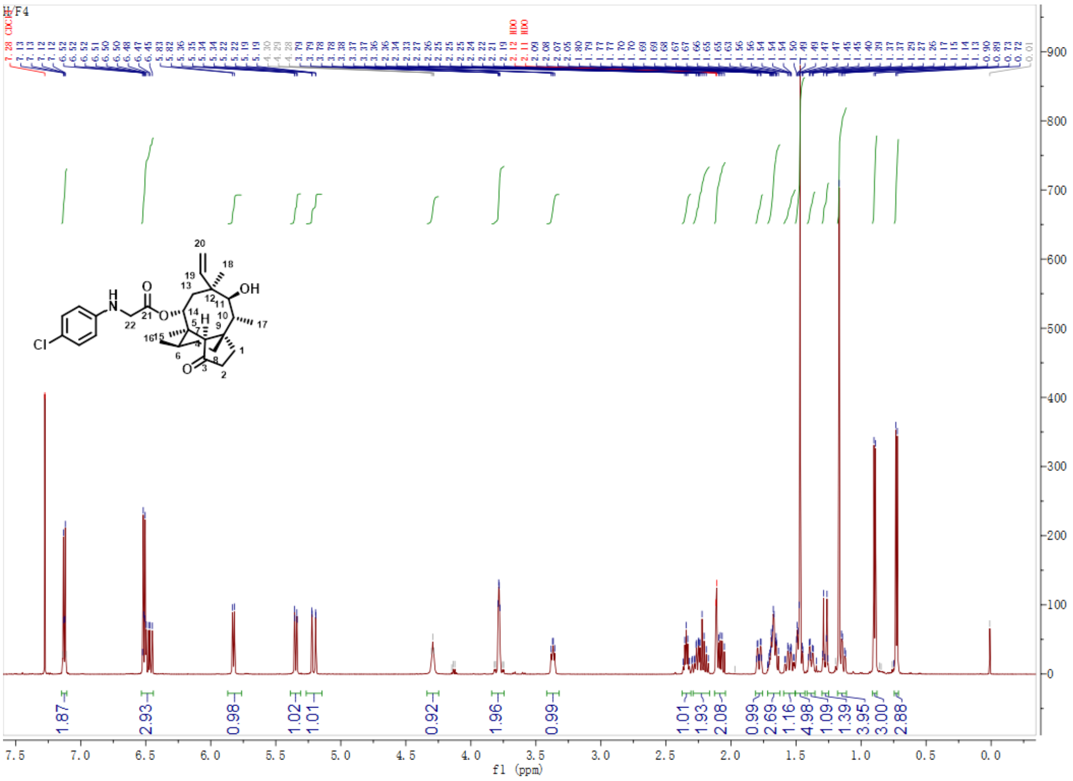

Figure SI 4-1. ^{1}H-NMR spectrum ($CDCl_3$, 400MHz) of compound **11**.

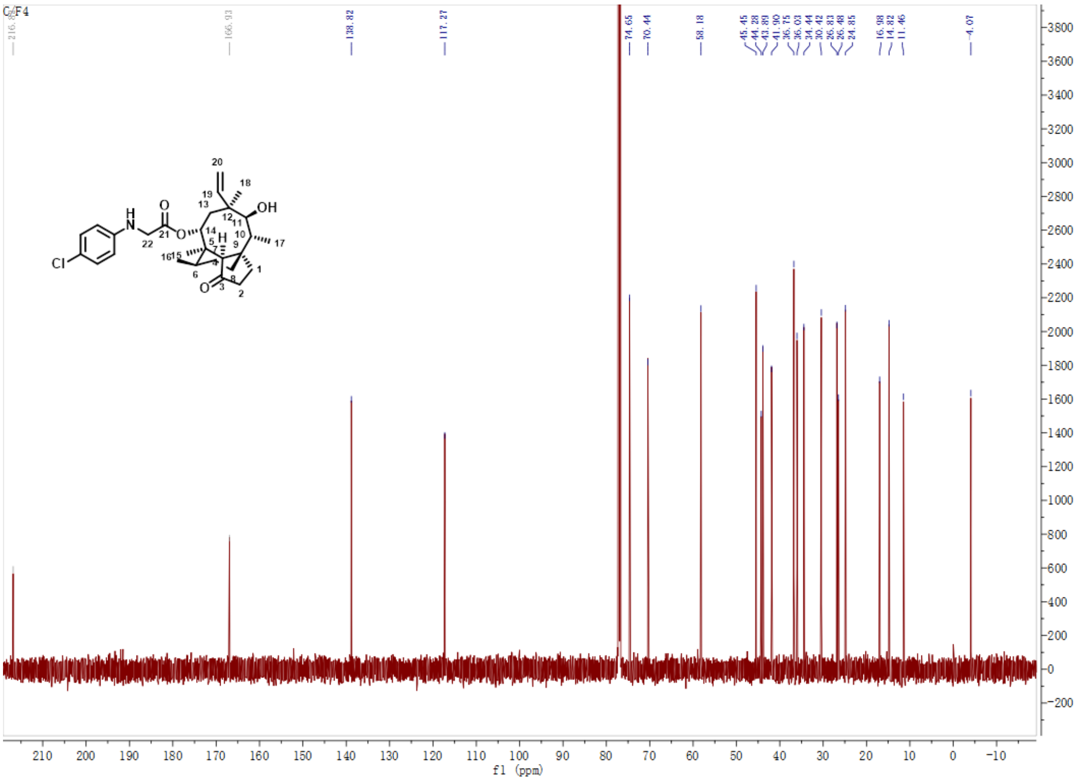

Figure SI 4-2. ^{13}C-NMR spectrum ($CDCl_3$, 101MHz) of compound **11**.

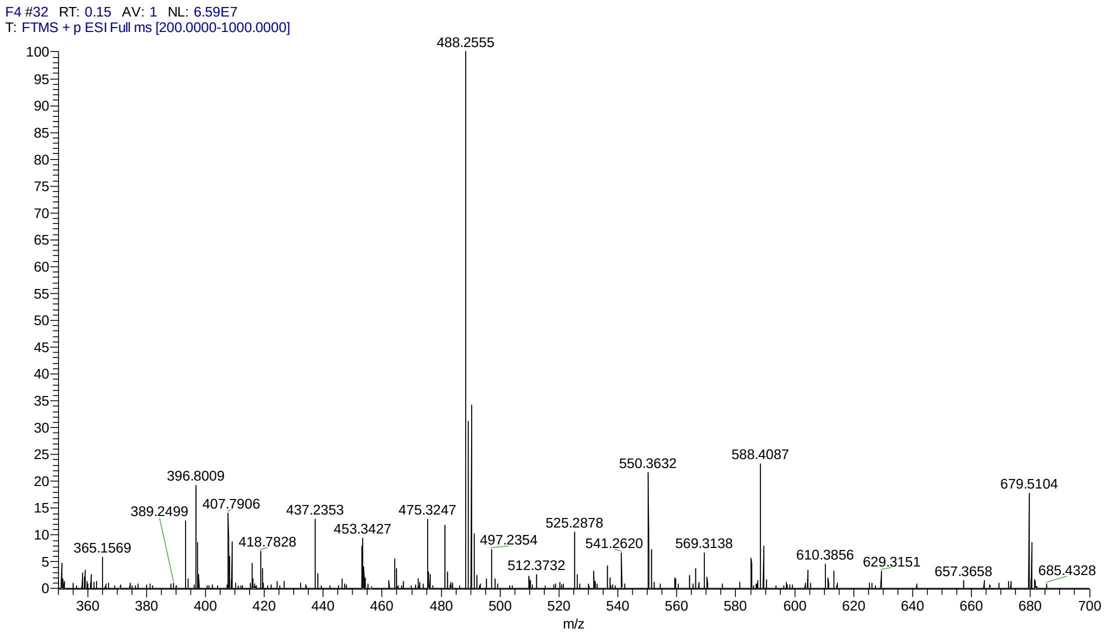

Figure SI 4-3. HR Mass spectrum (ESI) of compound **11**.

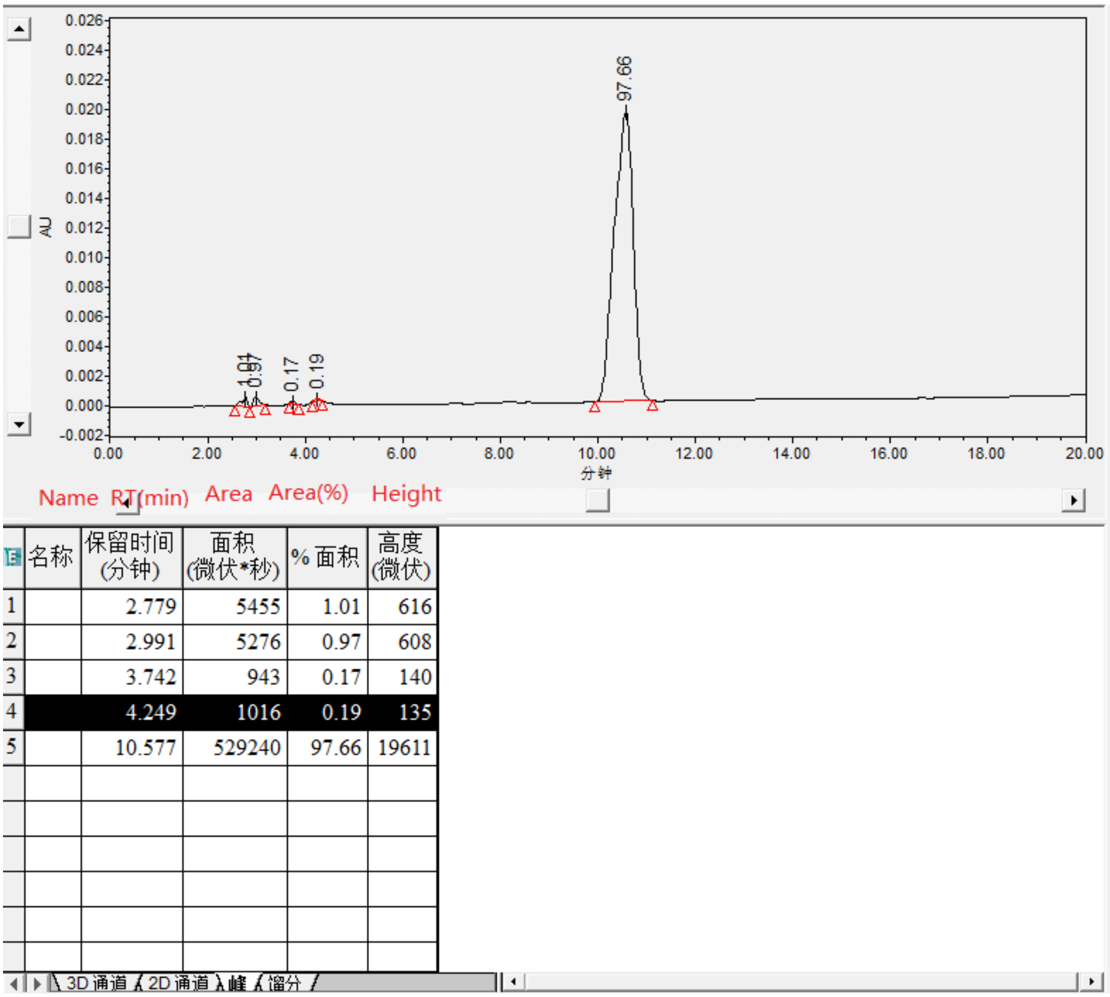

名称	保留时间 (分钟)	面积 (微伏*秒)	% 面积	高度 (微伏)
1	2.779	5455	1.01	616
2	2.991	5276	0.97	608
3	3.742	943	0.17	140
4	4.249	1016	0.19	135
5	10.577	529240	97.66	19611

Figure SI 4-4. HPLC of compound **11**.

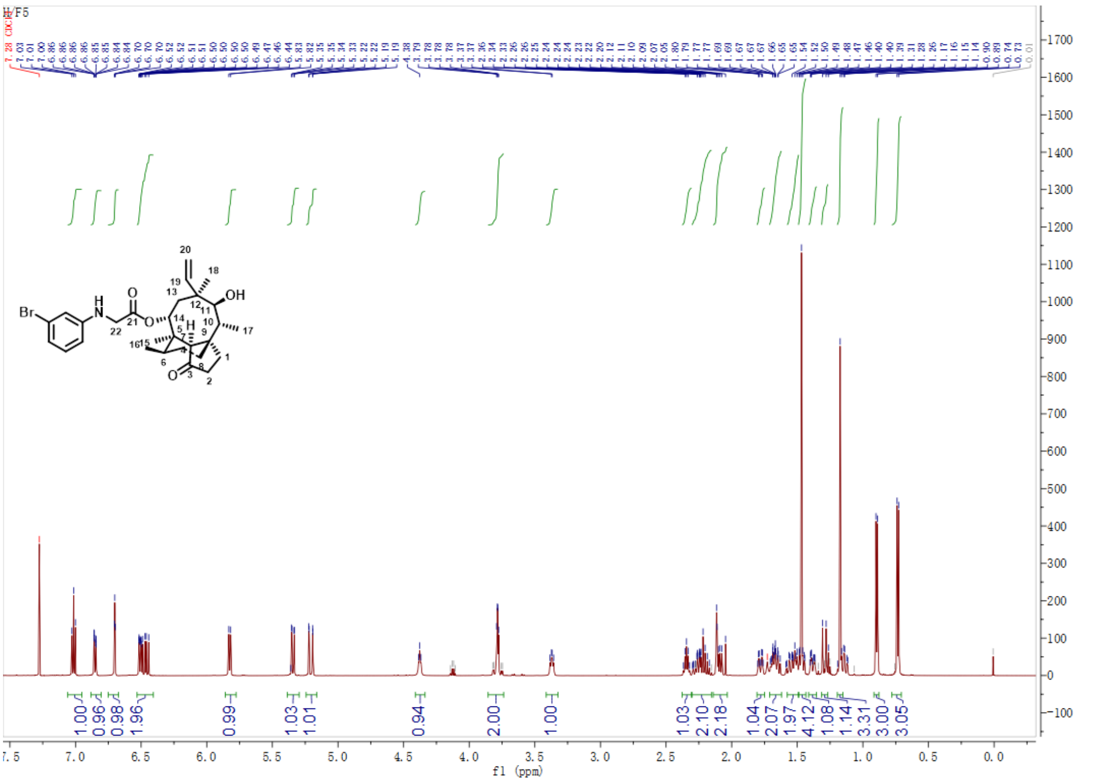

Figure SI 5-1. ^{1}H-NMR spectrum ($CDCl_3$, 400MHz) of compound **12**.

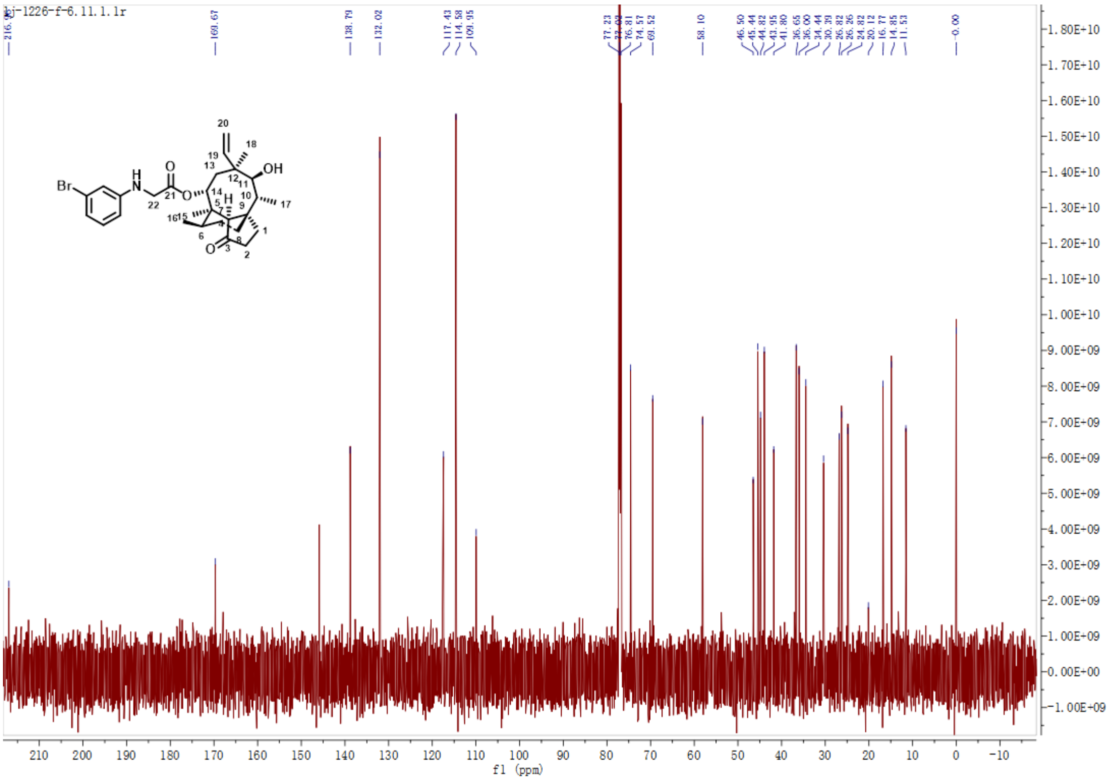

Figure SI 5-2. ^{13}C-NMR spectrum ($CDCl_3$, 101MHz) of compound **12**.

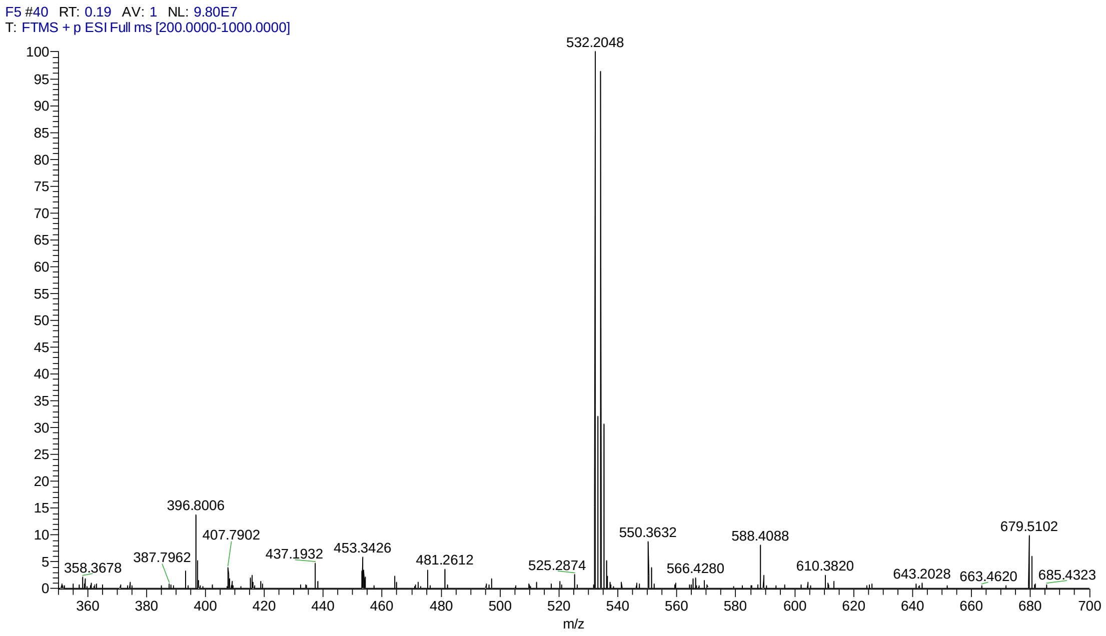

Figure SI 5-3. HR Mass spectrum (ESI) of compound **12**.

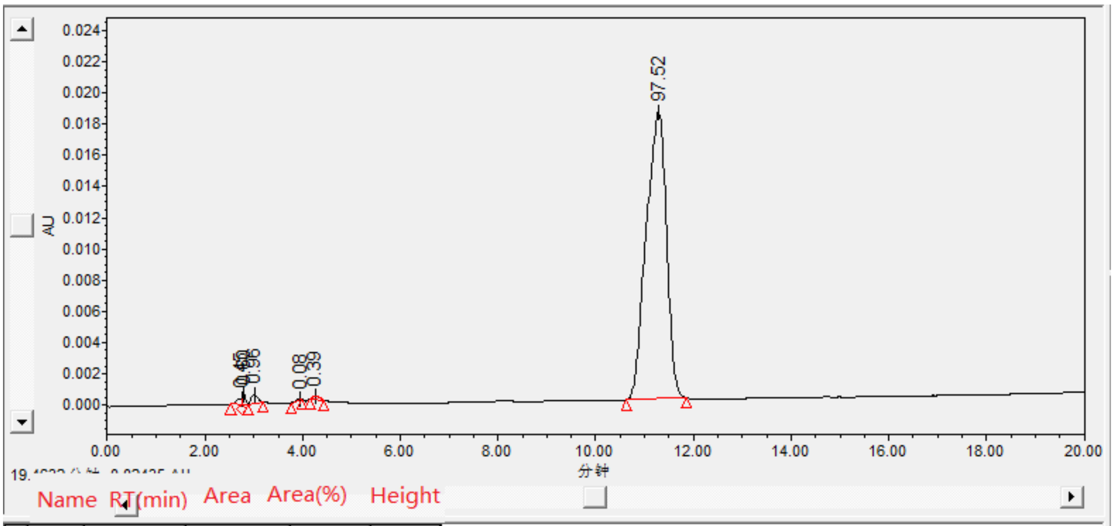

	名称	保留时间 (分钟)	面积 (微伏*秒)	% 面积	高度 (微伏)
1		2.750	2465	0.45	406
2		2.796	3270	0.60	641
3		3.009	5187	0.96	605
4		3.953	442	0.08	58
5		4.261	2092	0.39	208
6		11.288	529609	97.52	18414

Figure SI 5-4. HPLC of compound **12.**

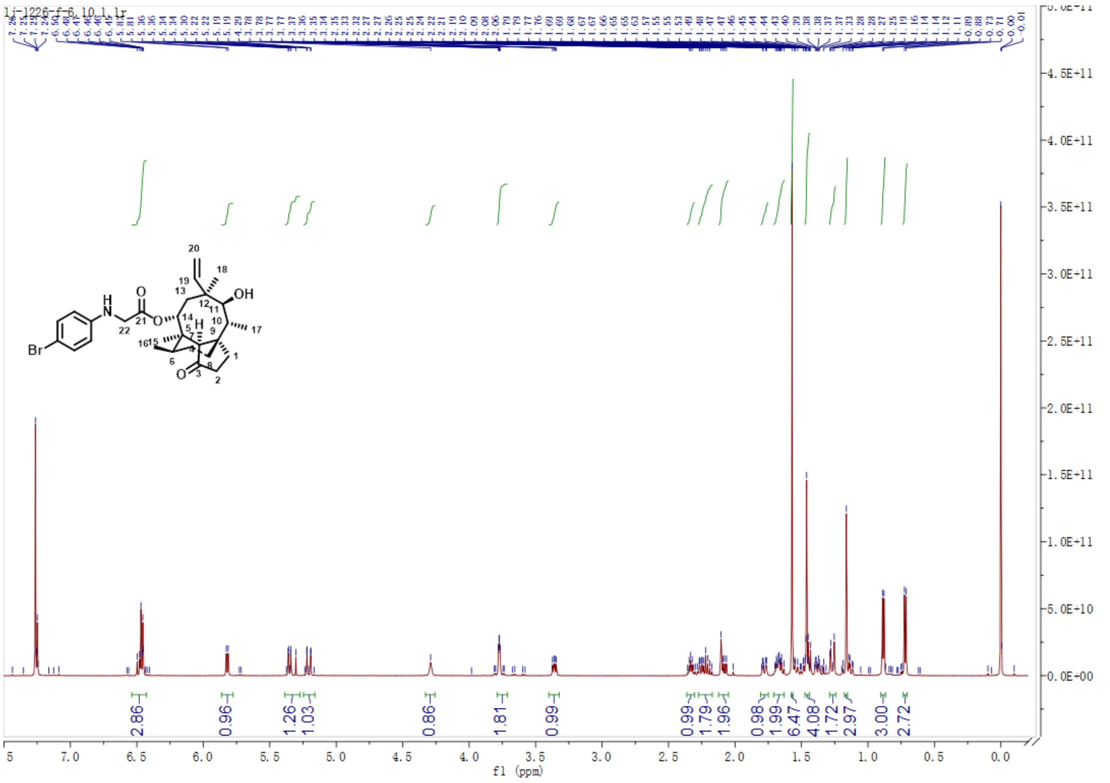

Figure SI 6-1. ^{1}H-NMR spectrum ($CDCl_3$, 400MHz) of compound **13**.

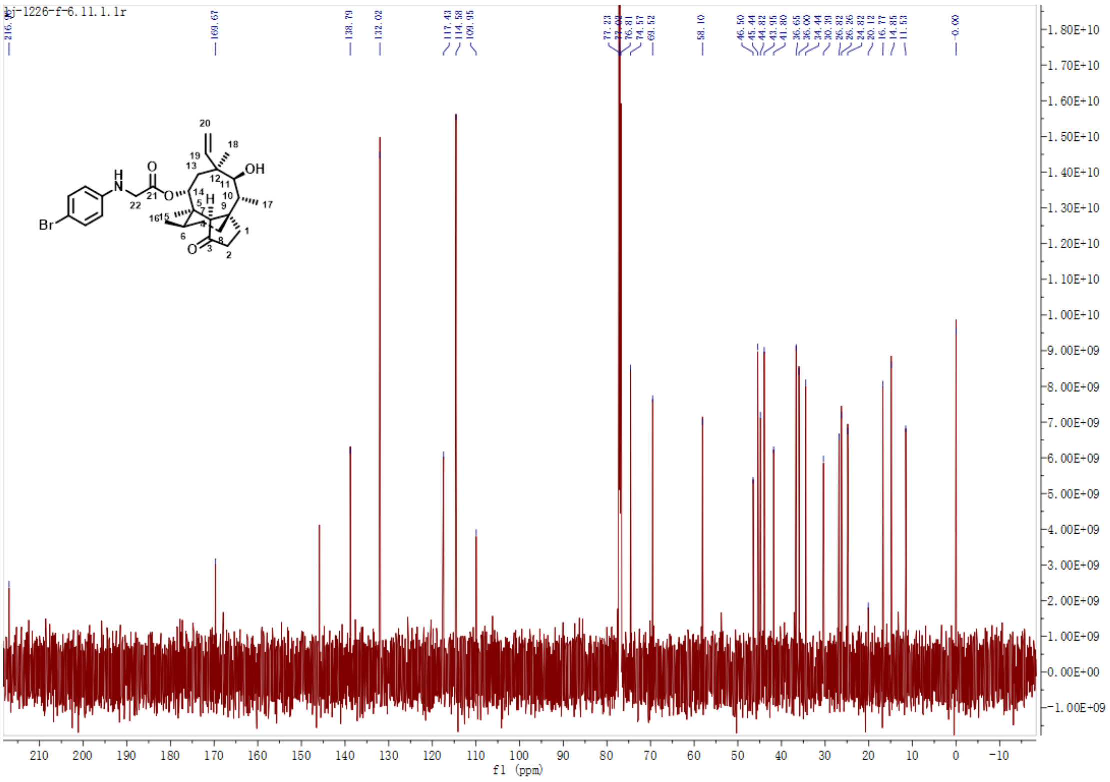

Figure SI 6-2. ^{13}C-NMR spectrum ($CDCl_3$, 101MHz) of compound **13**.

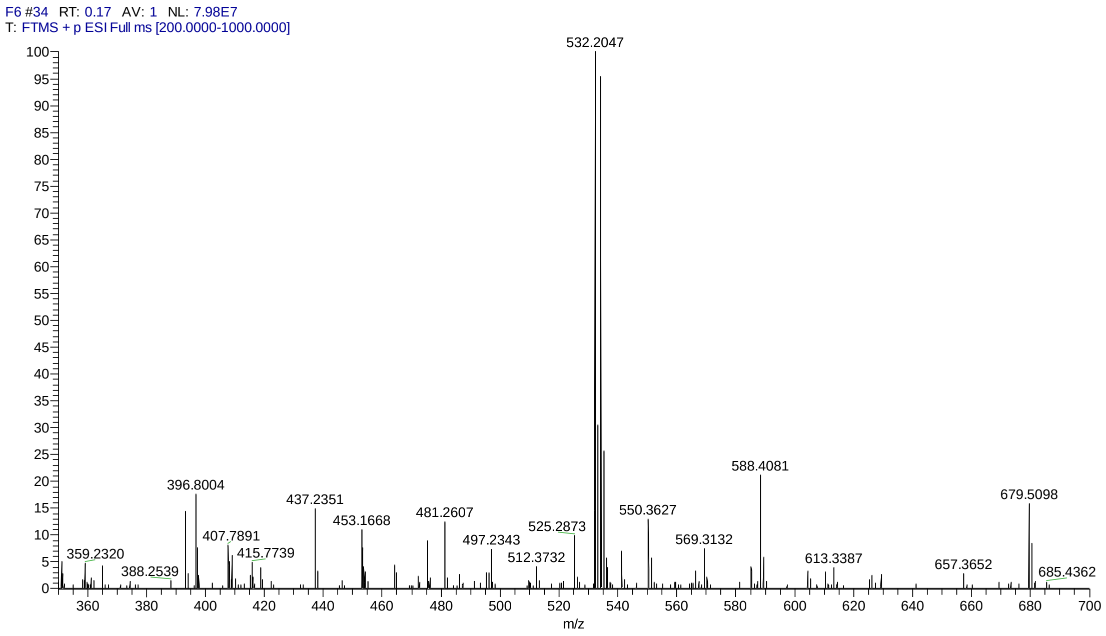

Figure SI 6-3. HR Mass spectrum (ESI) of compound **13**.

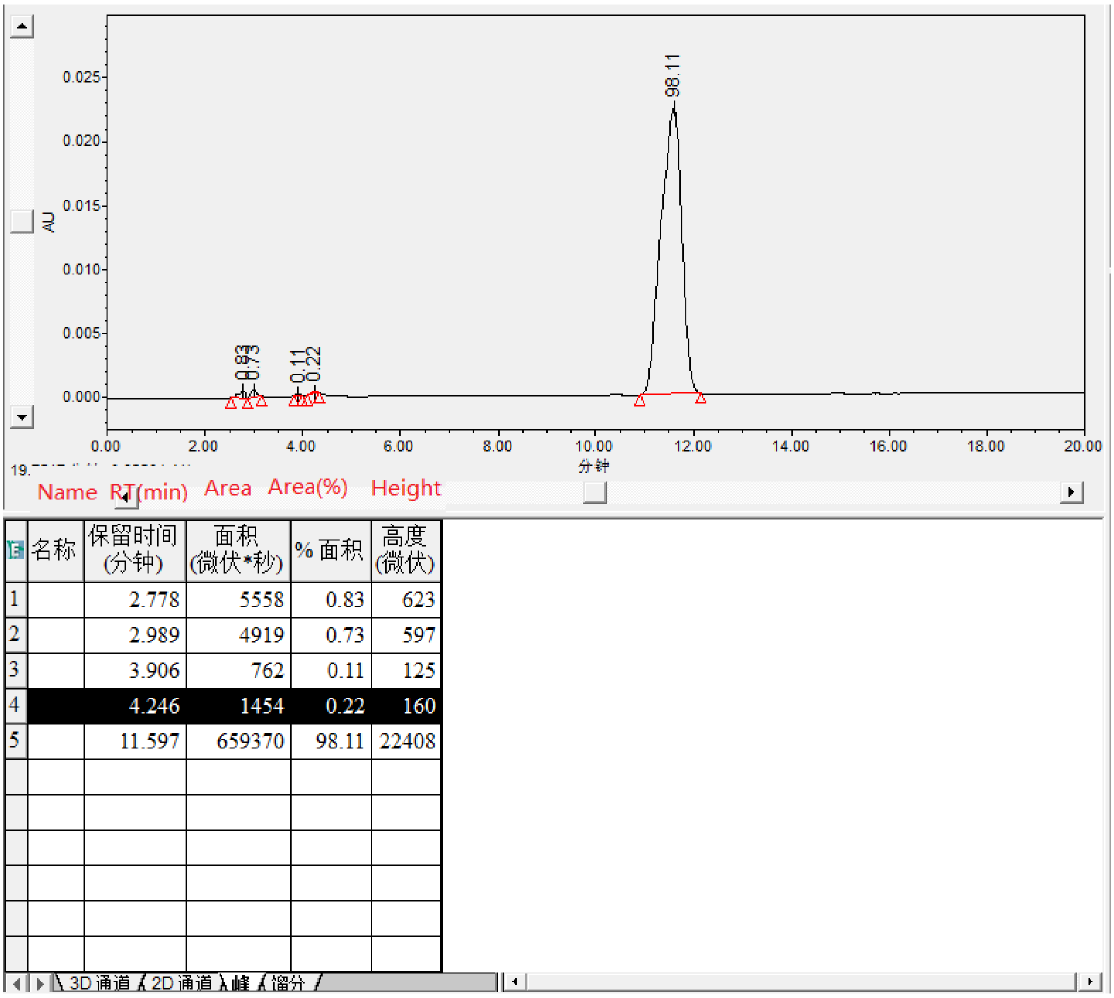

	名称	保留时间 (分钟)	面积 (微伏*秒)	% 面积	高度 (微伏)
1		2.778	5558	0.83	623
2		2.989	4919	0.73	597
3		3.906	762	0.11	125
4		4.246	1454	0.22	160
5		11.597	659370	98.11	22408

Figure SI 6-4. HPLC of compound **13.**

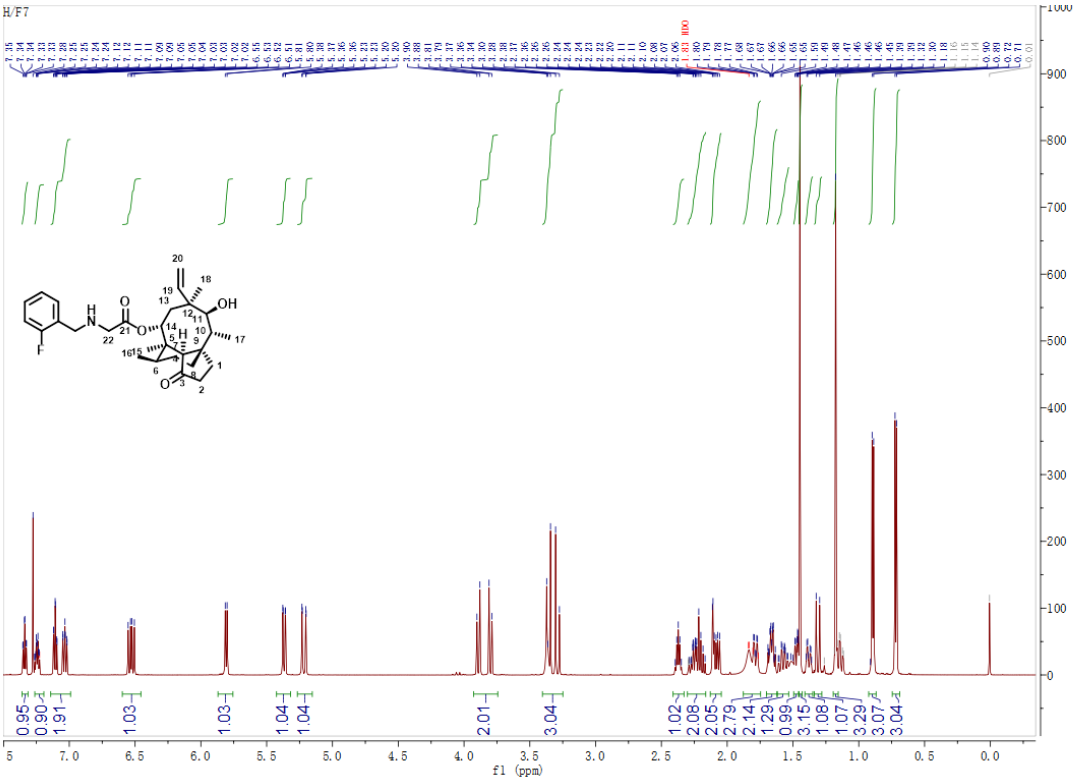

Figure SI 7-1. ^{1}H-NMR spectrum ($CDCl_3$, 400MHz) of compound **14**.

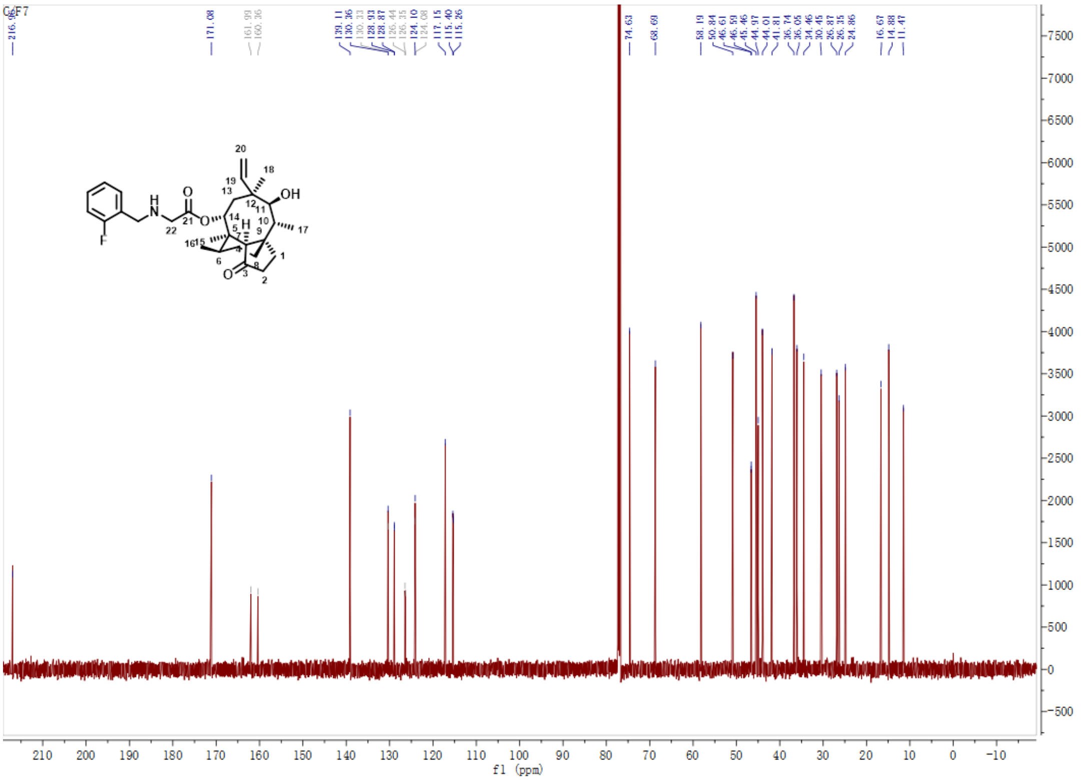

Figure SI 7-2. ^{13}C-NMR spectrum ($CDCl_3$, 101MHz) of compound **14**.

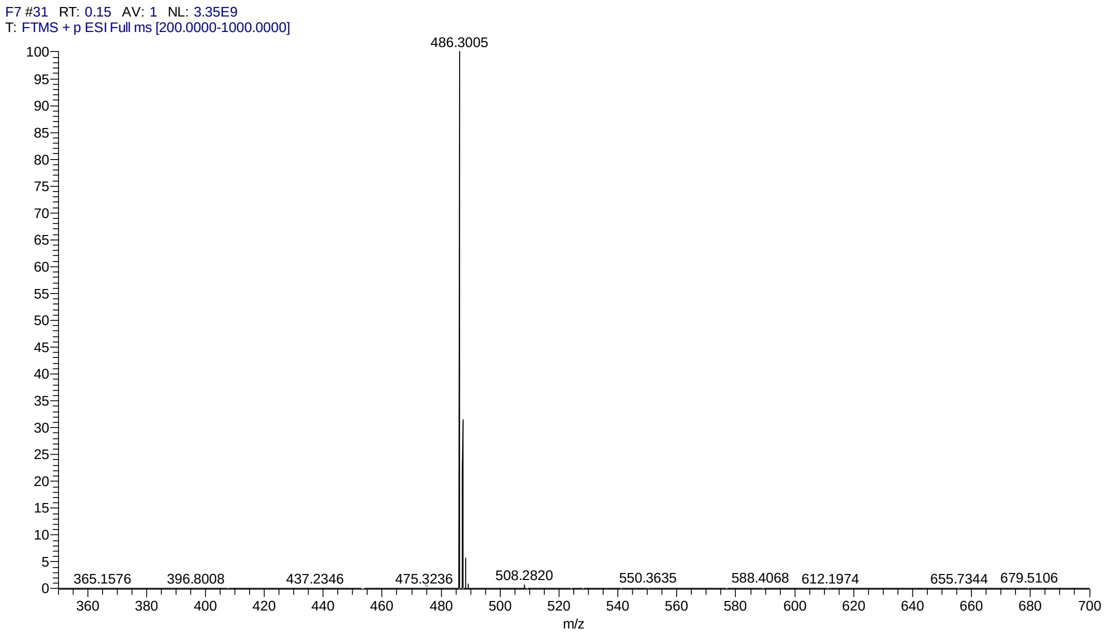

Figure SI 7-3. HR Mass spectrum (ESI) of compound **14**.

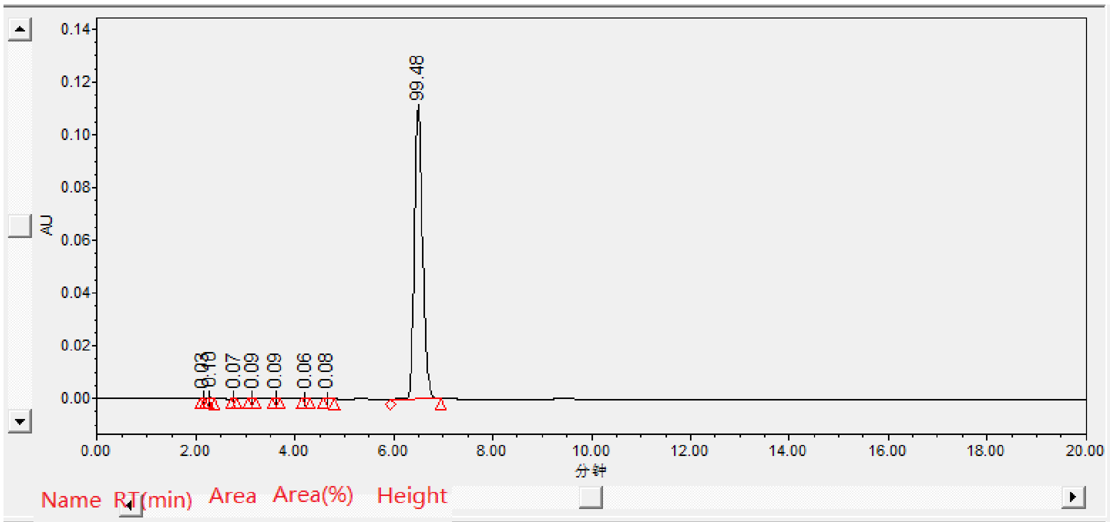

	名称	保留时间 (分钟)	面积 (微伏*秒)	% 面积	高度 (微伏)
1		2.139	334	0.03	100
2		2.274	1243	0.10	271
3		2.753	917	0.07	217
4		3.128	1204	0.09	233
5		3.607	1161	0.09	197
6		4.193	721	0.06	127
7		4.648	1018	0.08	136
8		6.489	1265596	99.48	109613

Figure SI 7-4. HPLC of compound **14**.

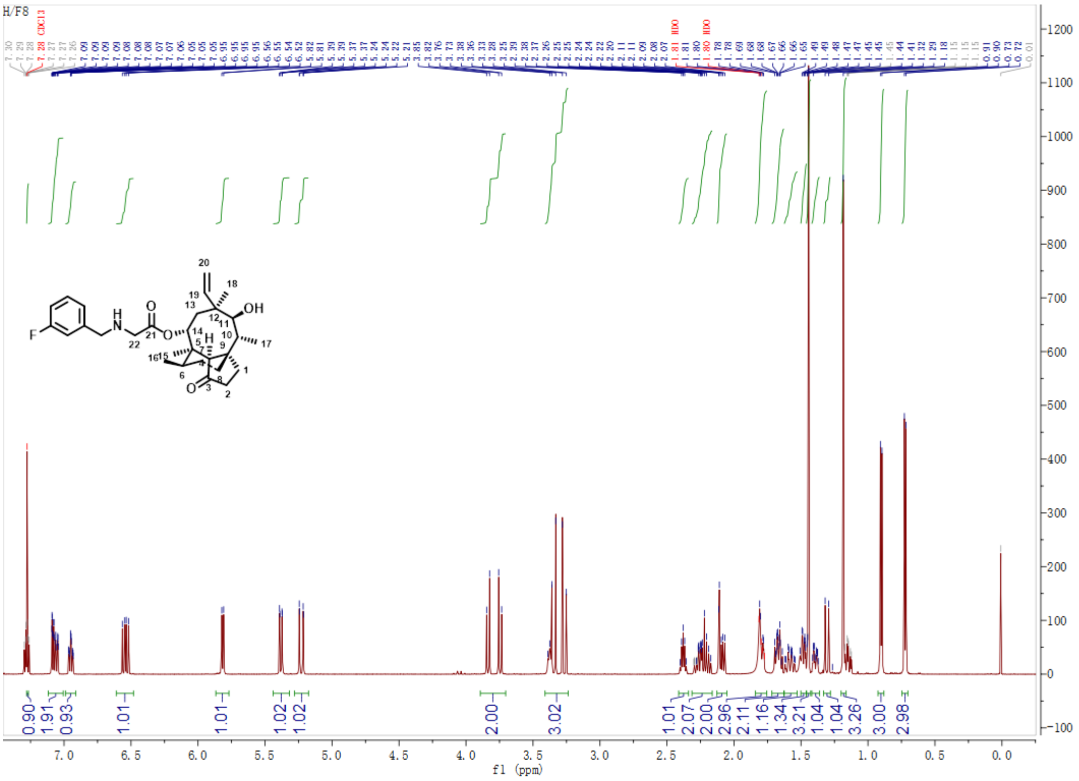

Figure SI 8-1. ^{1}H-NMR spectrum ($CDCl_3$, 400MHz) of compound **15**.

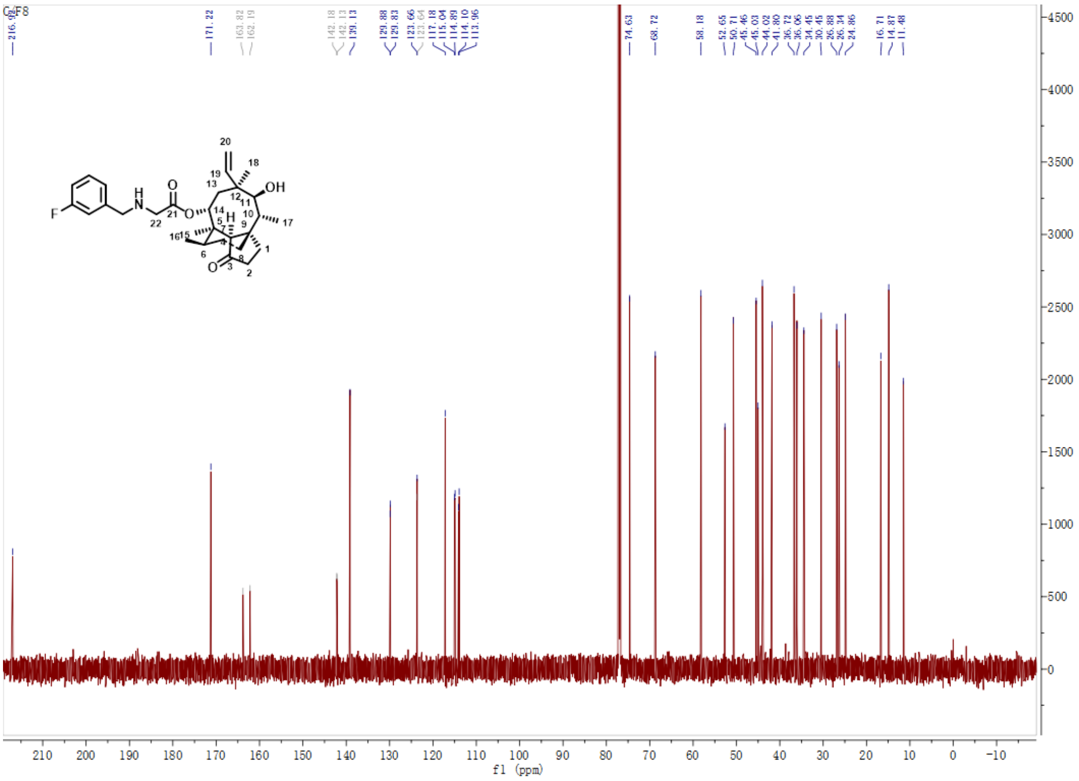

Figure SI 8-2. ^{13}C-NMR spectrum ($CDCl_3$, 101MHz) of compound **15**.

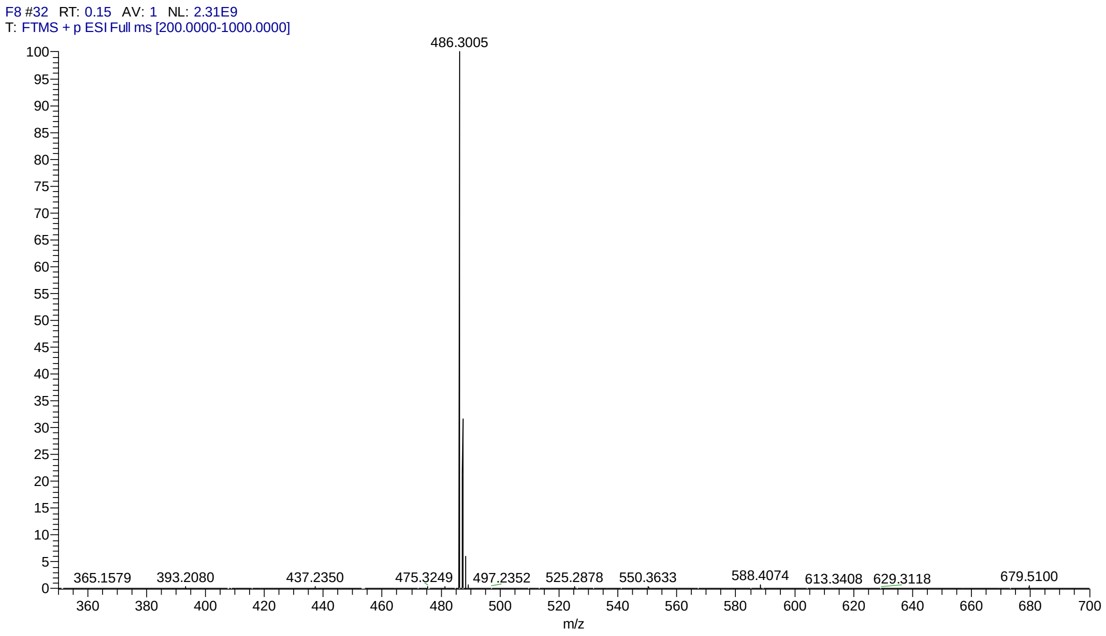

Figure SI 8-3. HR Mass spectrum (ESI) of compound **15**.

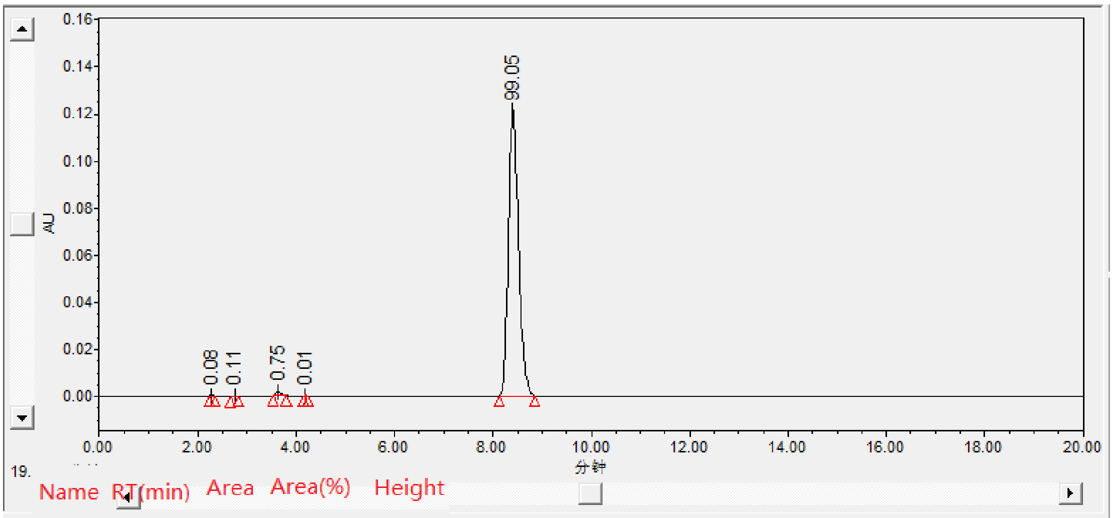

	名称	保留时间 (分钟)	面积 (微伏*秒)	% 面积	高度 (微伏)
1		2.281	1375	0.08	332
2		2.756	1954	0.11	346
3		3.632	13112	0.75	1773
4		4.190	168	0.01	53
5		8.408	1729172	99.05	121916

Figure SI 8-4. HPLC of compound **15**.

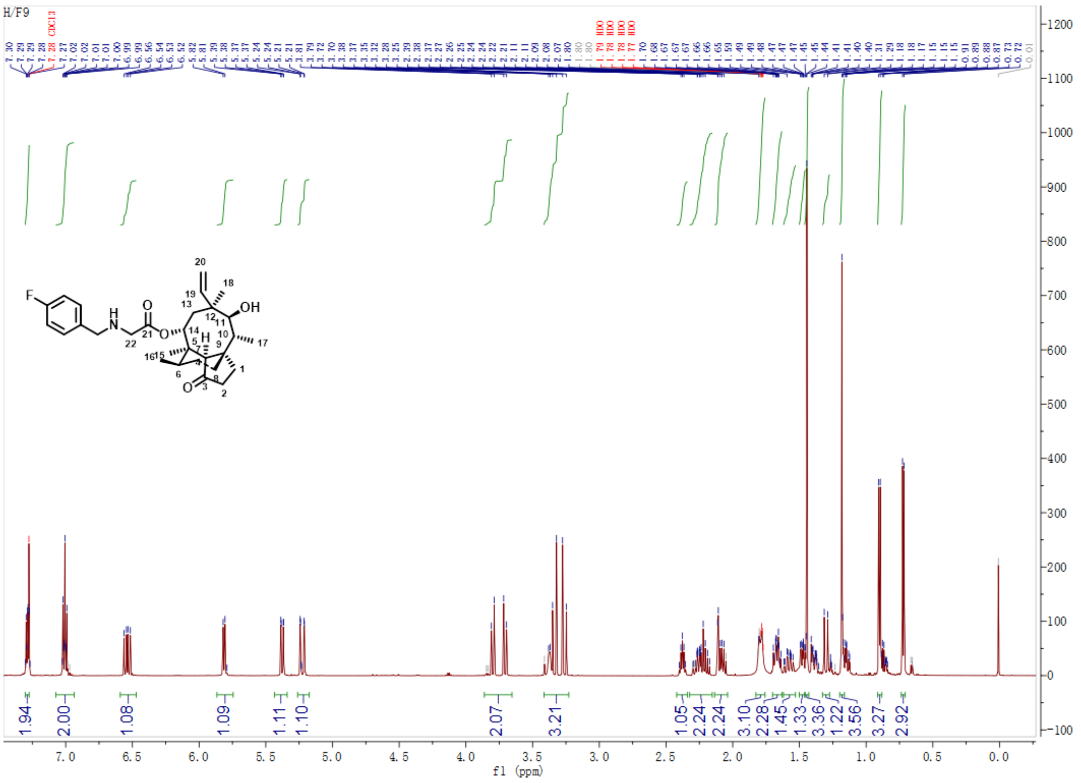

Figure SI 9-1. ^{1}H-NMR spectrum ($CDCl_3$, 400MHz) of compound **16**.

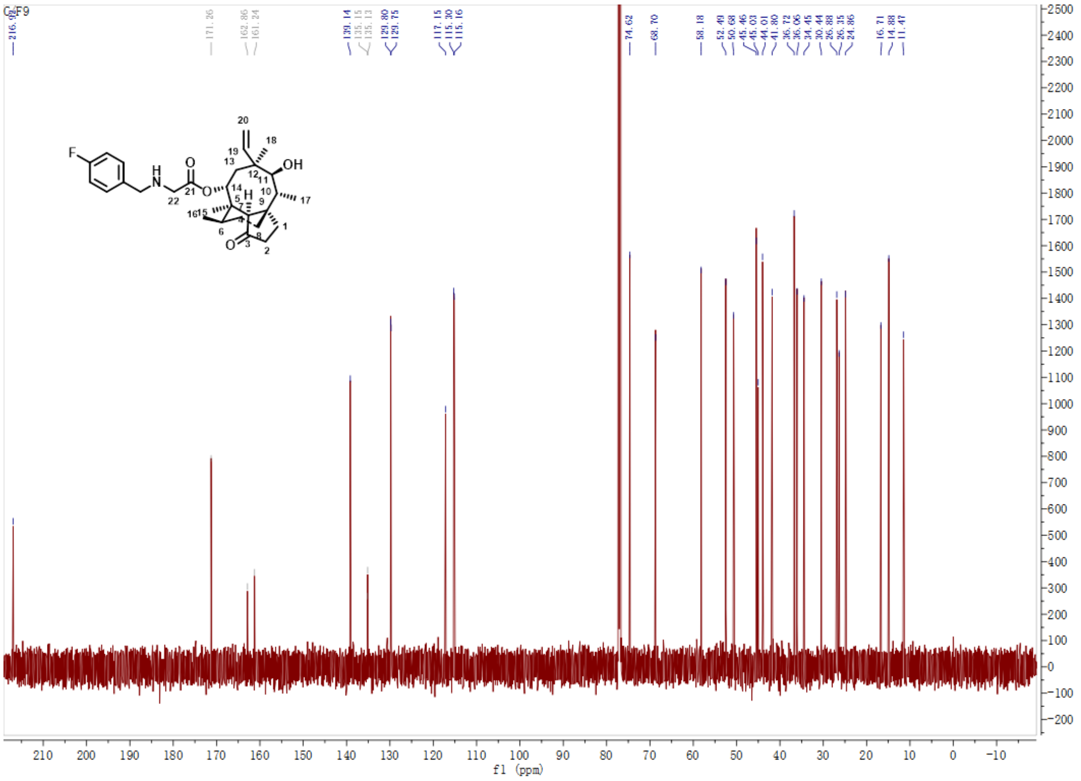

Figure SI 9-2. ^{13}C-NMR spectrum ($CDCl_3$, 101MHz) of compound **16**.

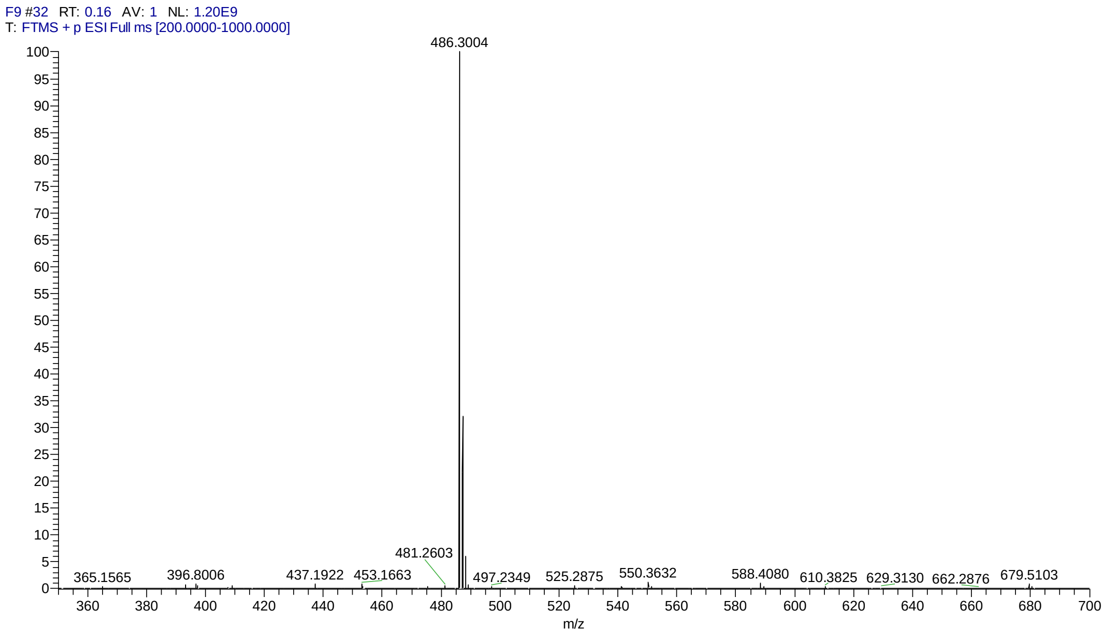

Figure SI 9-3. HR Mass spectrum (ESI) of compound **16**.

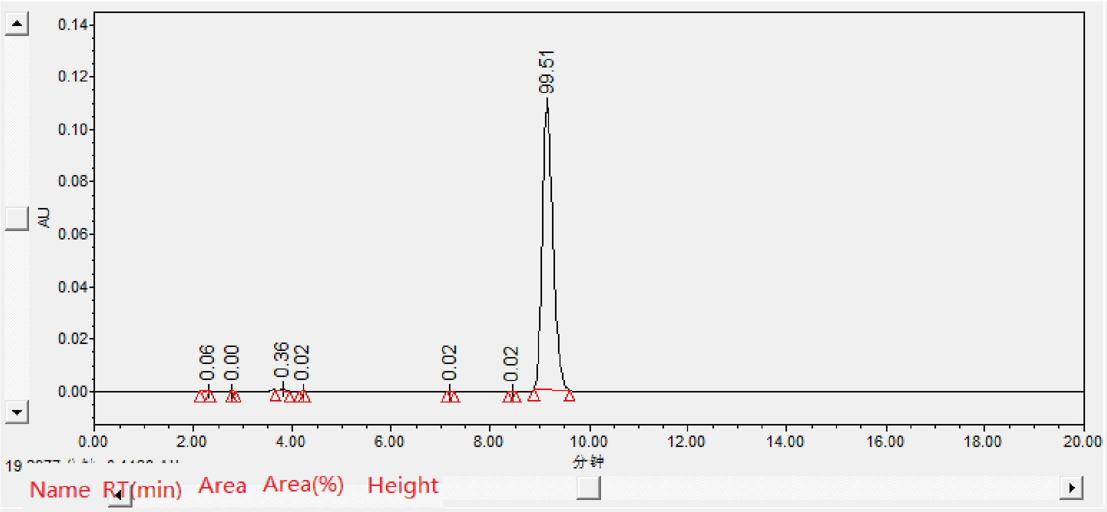

	名称	保留时间 (分钟)	面积 (微伏*秒)	% 面积	高度 (微伏)
1		2.294	1064	0.06	204
2		2.783	72	0.00	31
3		3.803	5942	0.36	894
4		4.208	384	0.02	87
5		7.186	387	0.02	70
6		8.446	319	0.02	49
7		9.148	1649080	99.51	108989

Figure SI 9-4. HPLC of compound **16**.

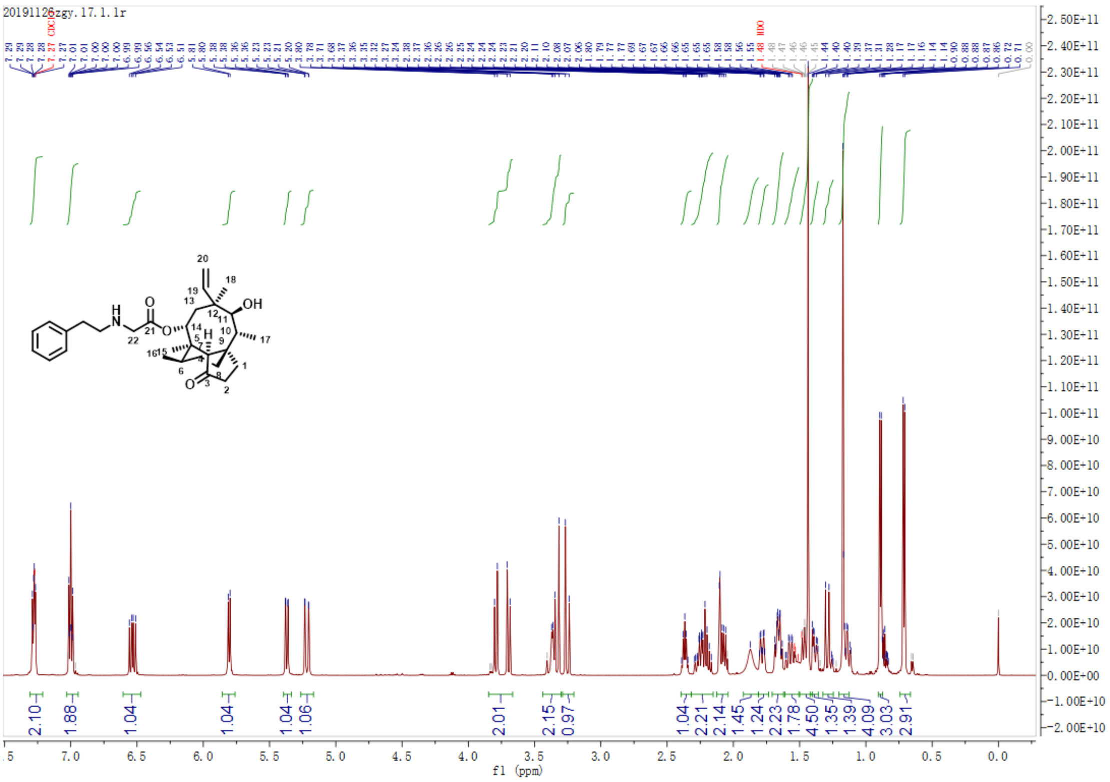

Figure SI 10-1. ^{1}H-NMR spectrum ($CDCl_3$, 400MHz) of compound **17**.

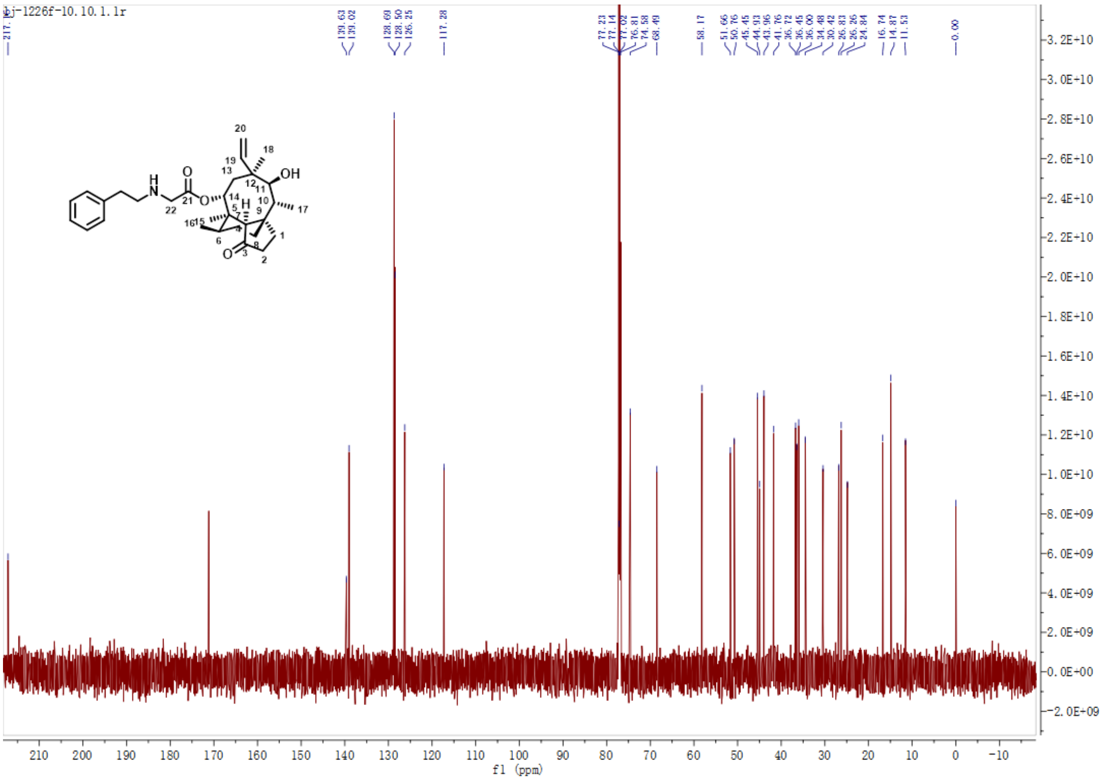

Figure SI 10-2. ^{13}C-NMR spectrum ($CDCl_3$, 101MHz) of compound **17**.

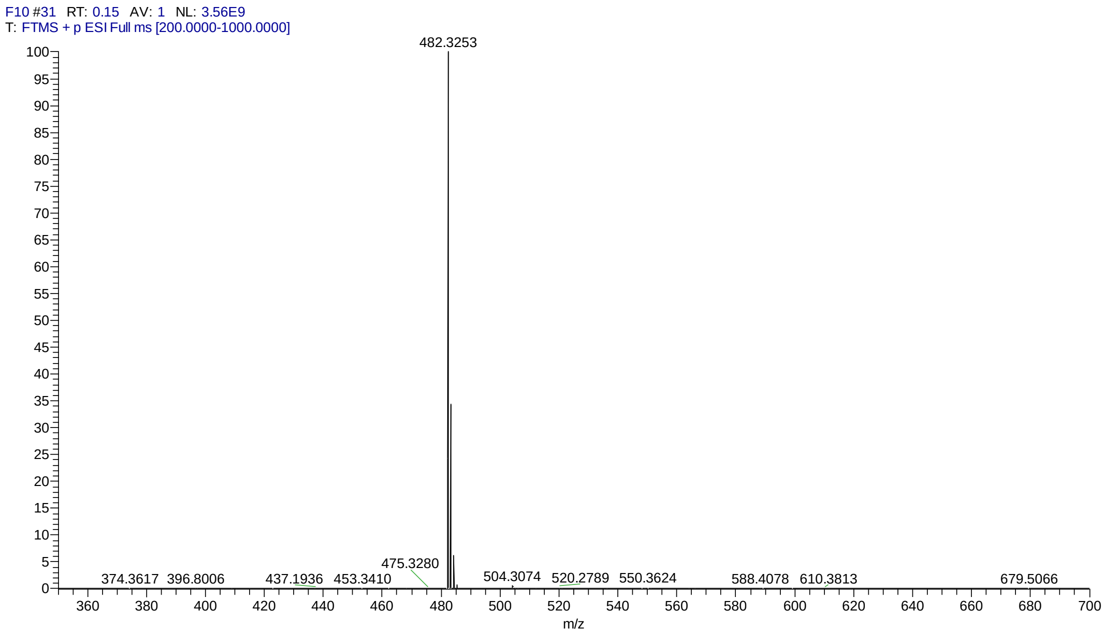

Figure SI 10-3. HR Mass spectrum (ESI) of compound **17**.

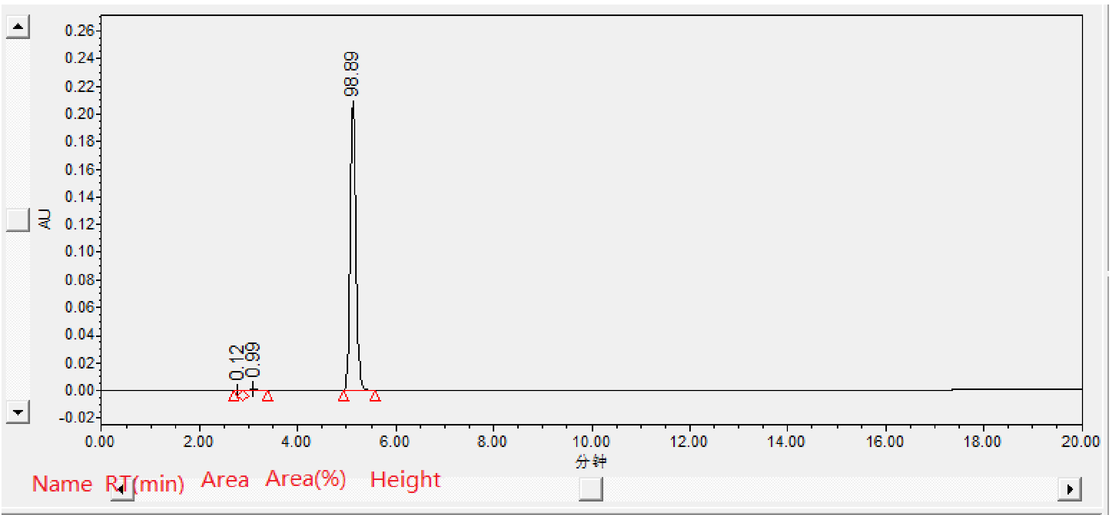

	名称	保留时间 (分钟)	面积 (微伏*秒)	% 面积	高度 (微伏)
1		2.779	2010	0.12	276
2		3.100	17130	0.99	1741
3		5.127	1703915	98.89	205985

Figure SI 10-4. HPLC of compound **17**.

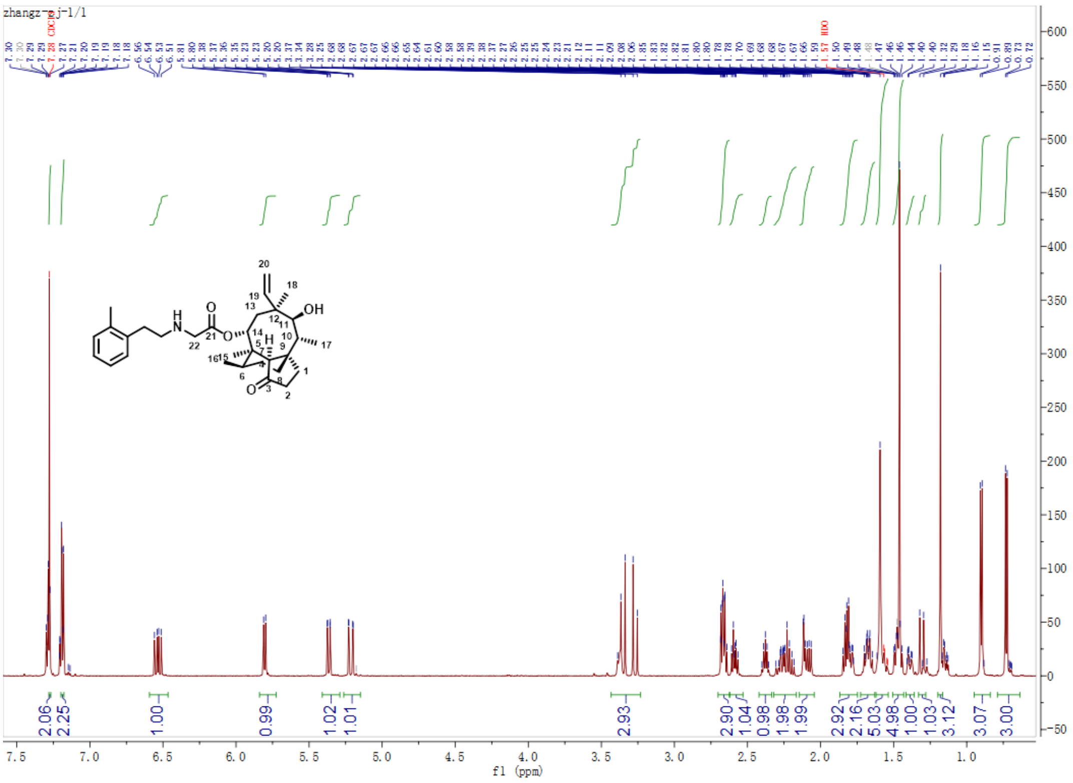

Figure SI 11-1. ^{1}H-NMR spectrum ($CDCl_3$, 400MHz) of compound **18**.

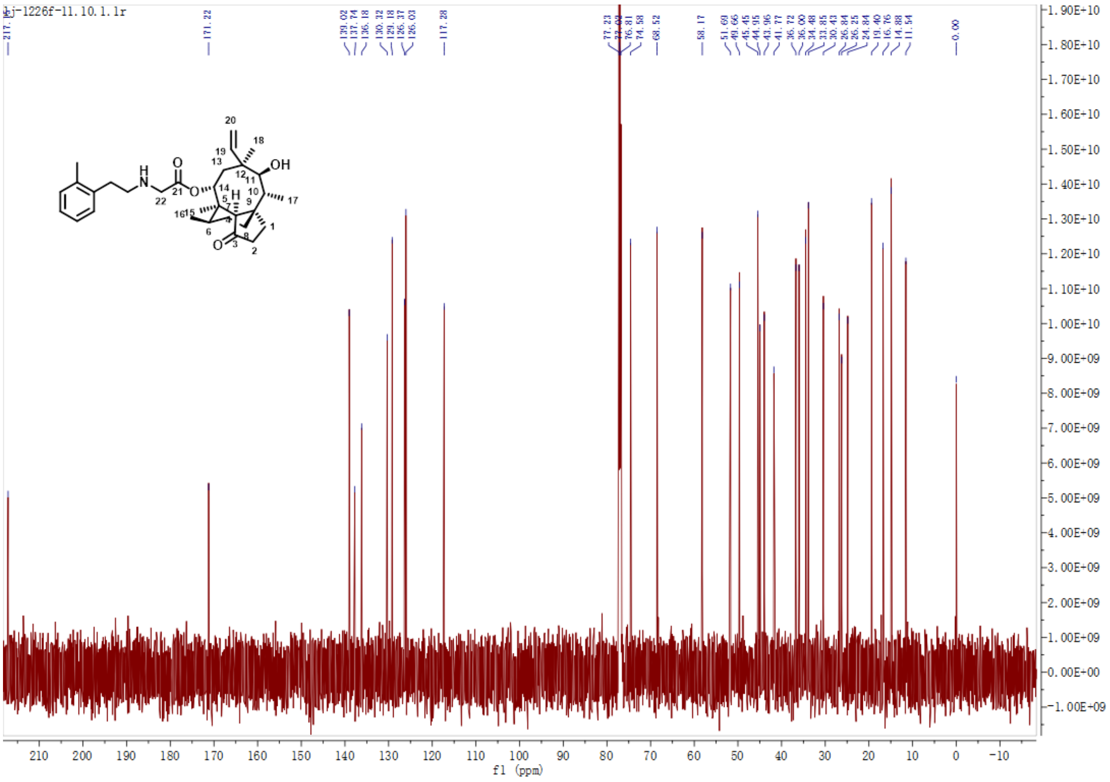

Figure SI 11-2. ^{13}C-NMR spectrum ($CDCl_3$, 101MHz) of compound **18**.

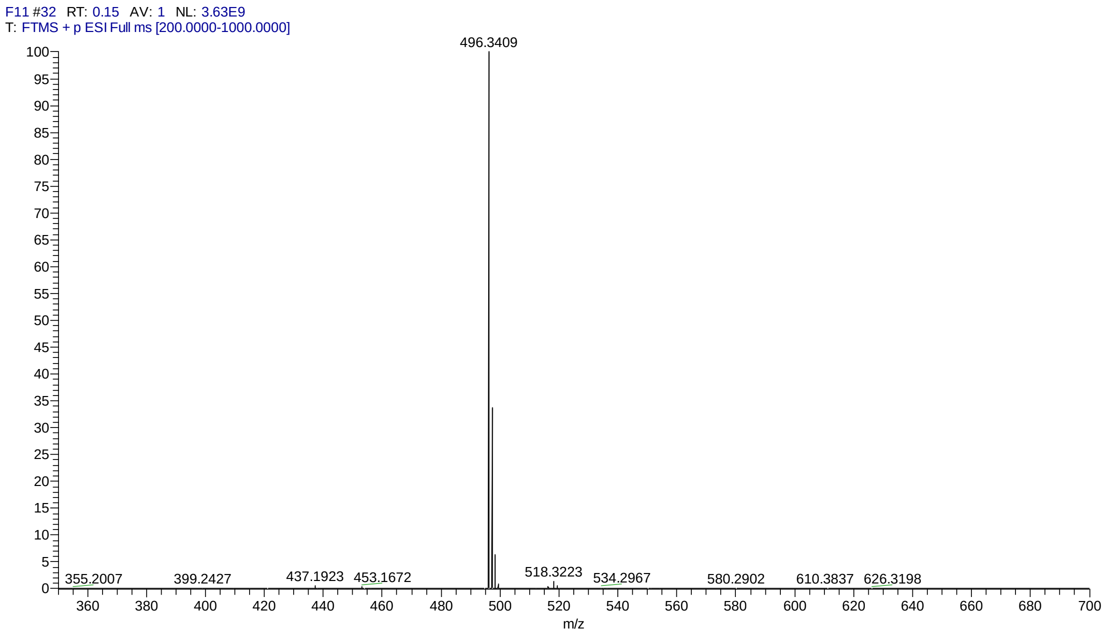

Figure SI 11-3. HR Mass spectrum (ESI) of compound **18**.

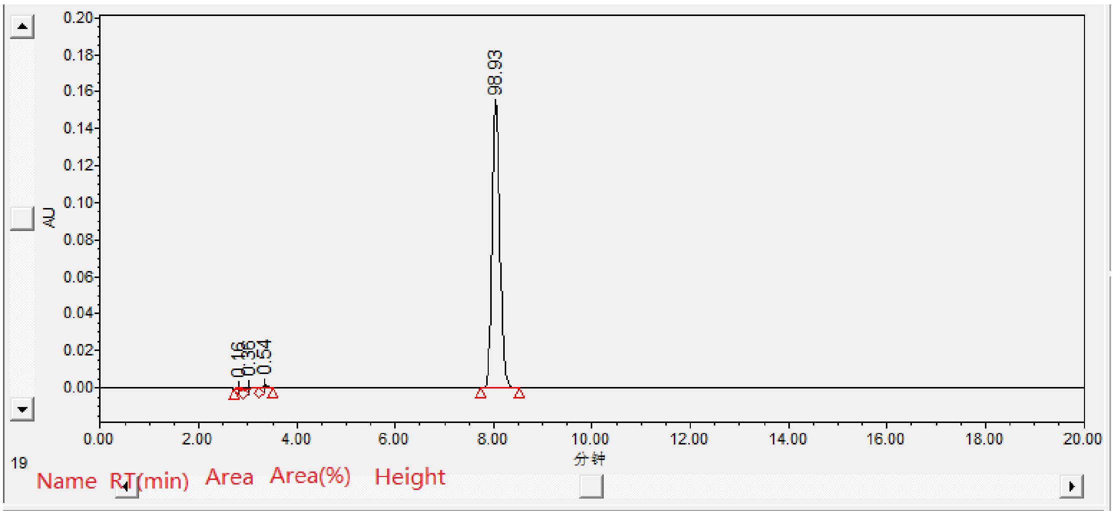

	名称	保留时间 (分钟)	面积 (微伏*秒)	% 面积	高度 (微伏)
1		2.815	2770	0.16	356
2		3.026	6276	0.36	539
3		3.352	9367	0.54	1444
4		8.043	1702941	98.93	153036

Figure SI 11-4. HPLC of compound 1**8.**

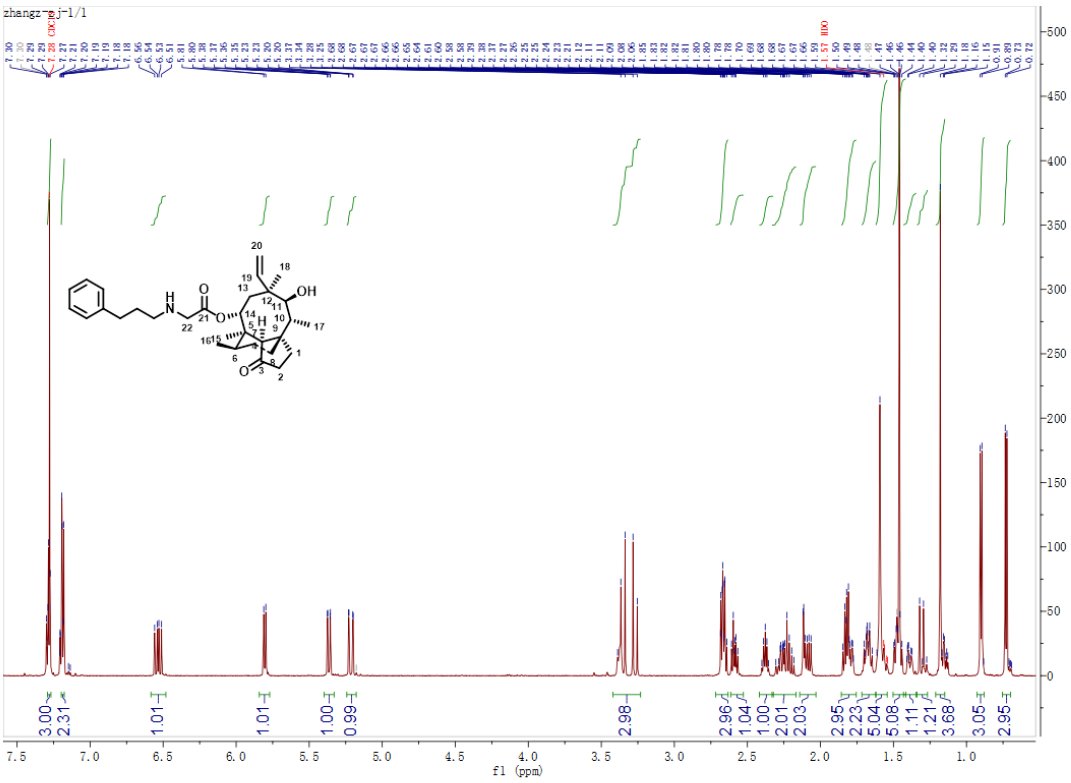

Figure SI 12-1. ^{1}H-NMR spectrum ($CDCl_3$, 400MHz) of compound **19**.

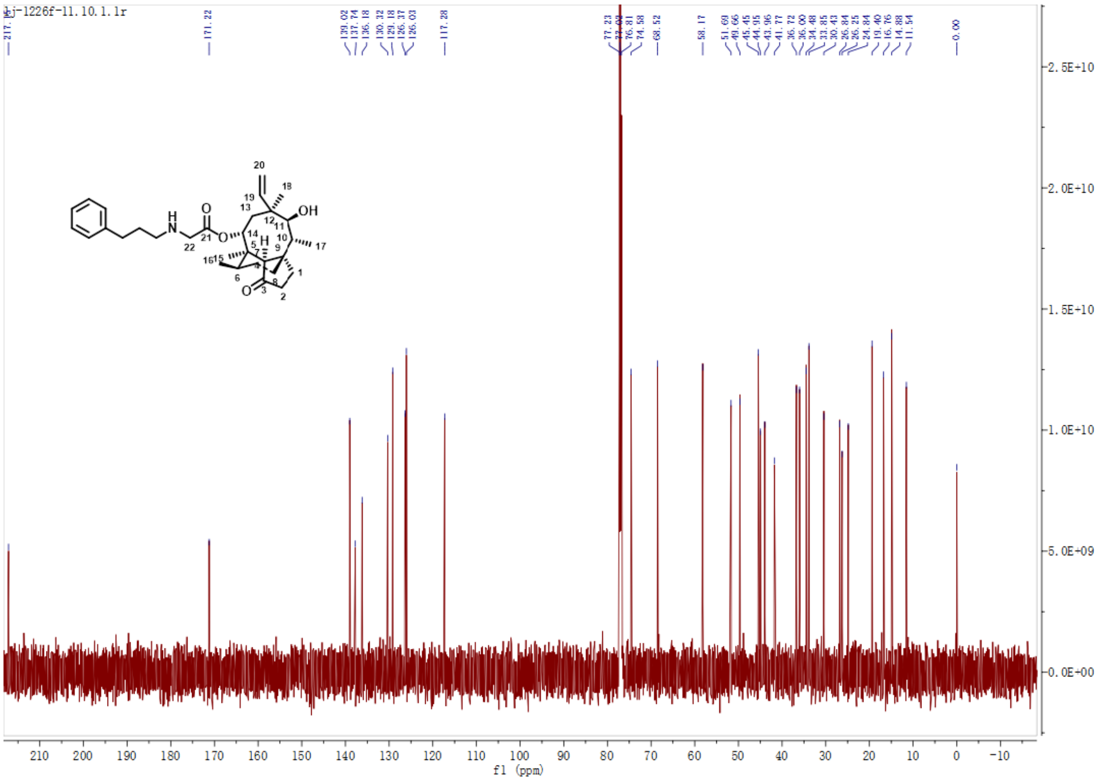

Figure SI 12-2. ^{13}C-NMR spectrum ($CDCl_3$, 101MHz) of compound **19**.

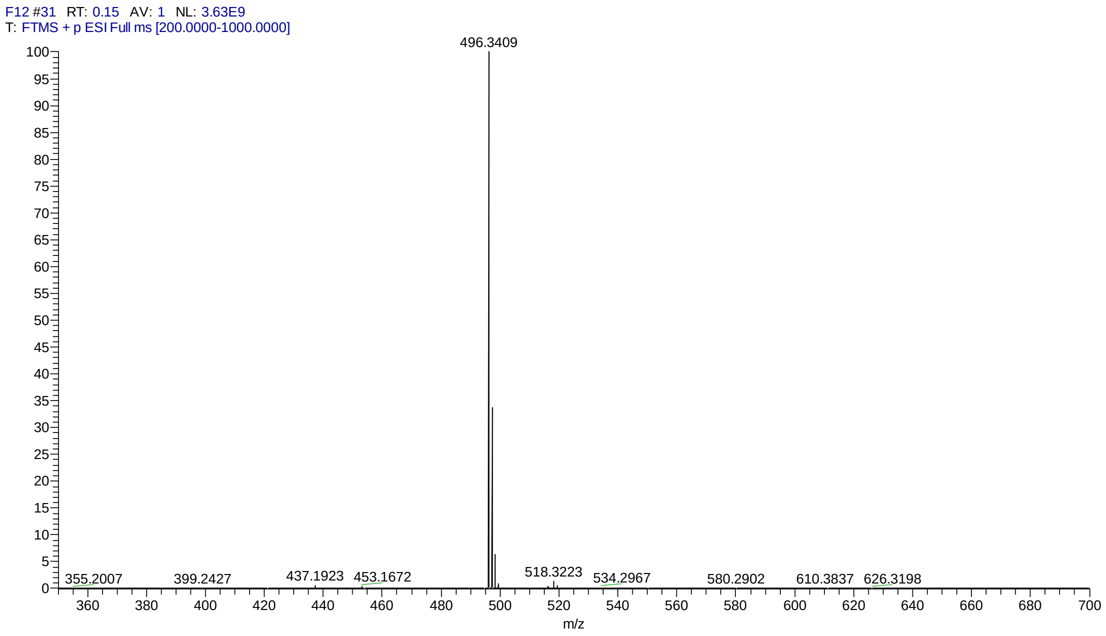

Figure SI 12-3. HR Mass spectrum (ESI) of compound **19**.

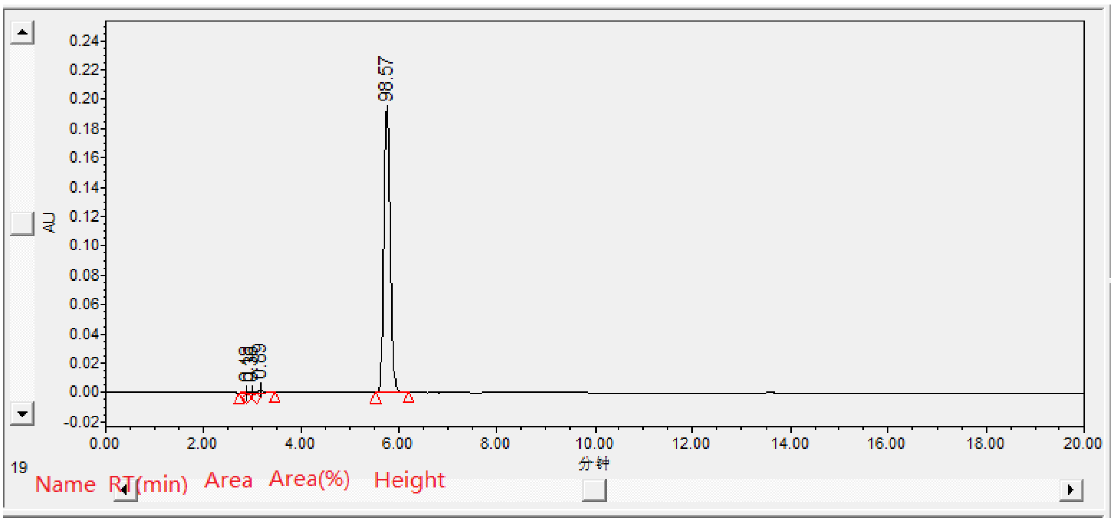

	名称	保留时间 (分钟)	面积 (微伏*秒)	% 面积	高度 (微伏)
1		2.883	3127	0.18	409
2		2.974	6173	0.36	677
3		3.161	15410	0.89	1790
4		5.739	1707769	98.57	192505

Figure SI 12-4. HPLC of compound **19.**

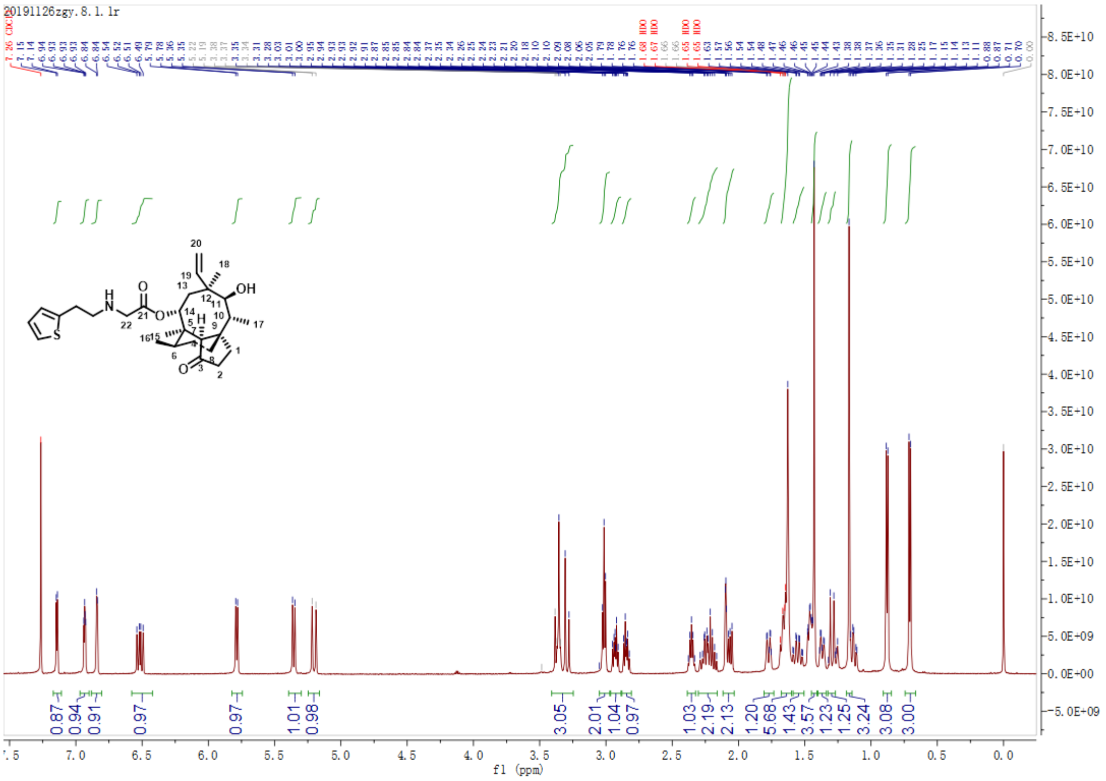

Figure SI 13-1. ^{1}H-NMR spectrum ($CDCl_3$, 400MHz) of compound **20**.

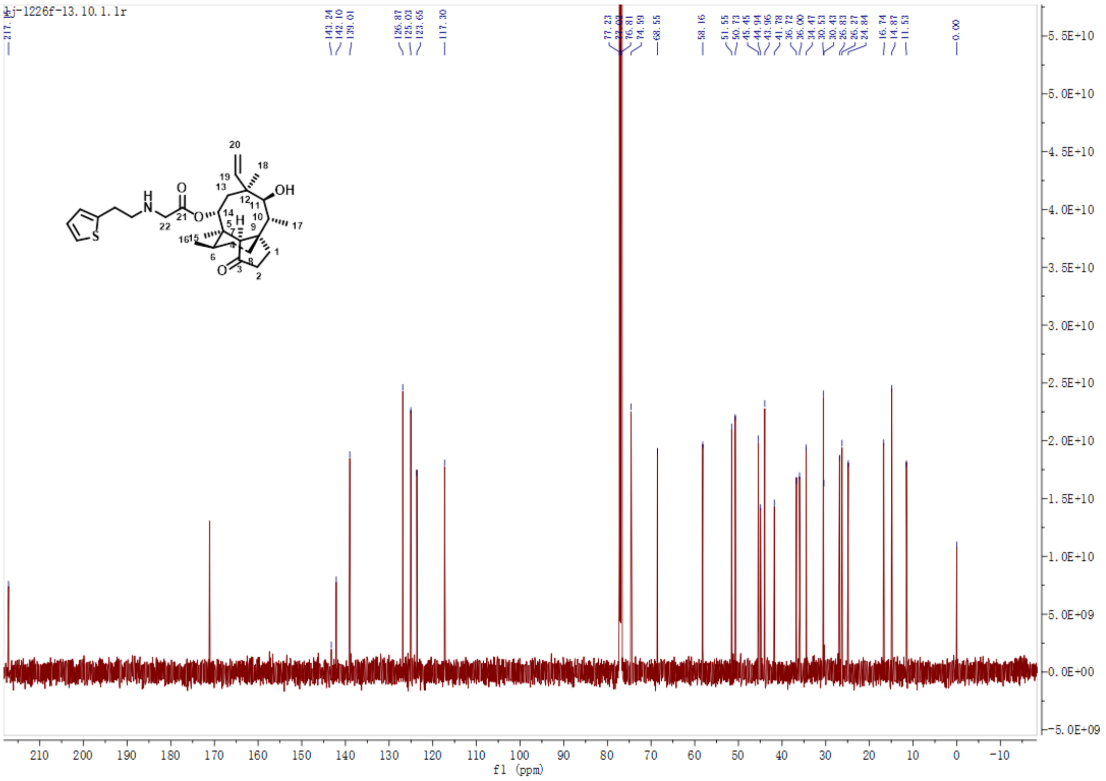

Figure SI 13-2. ^{13}C-NMR spectrum ($CDCl_3$, 101MHz) of compound **20**.

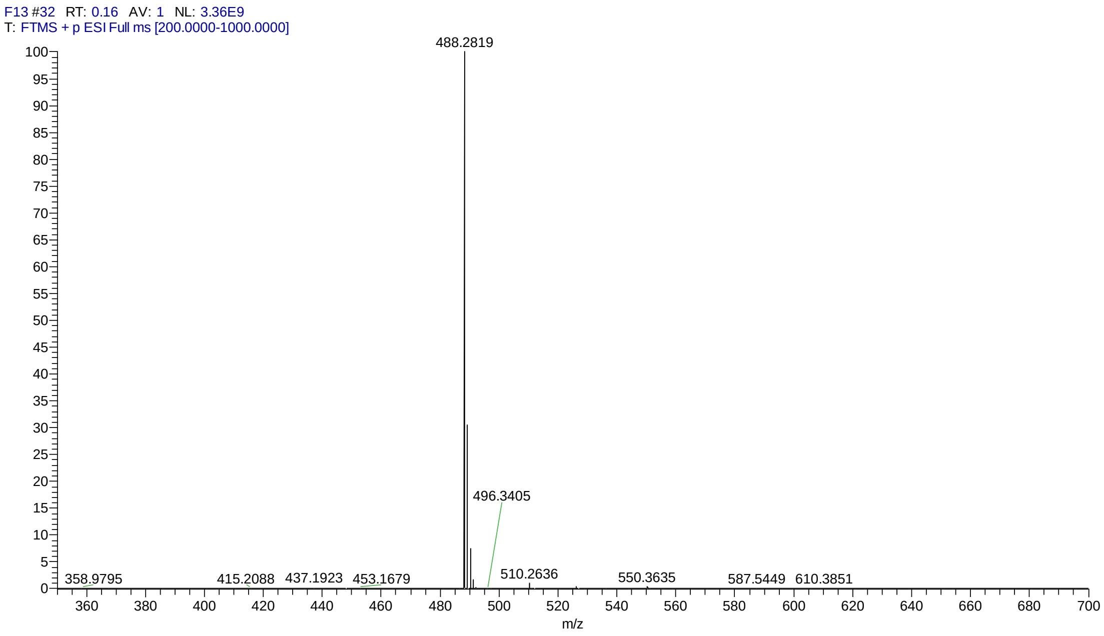

Figure SI 13-3. HR Mass spectrum (ESI) of compound **20**.

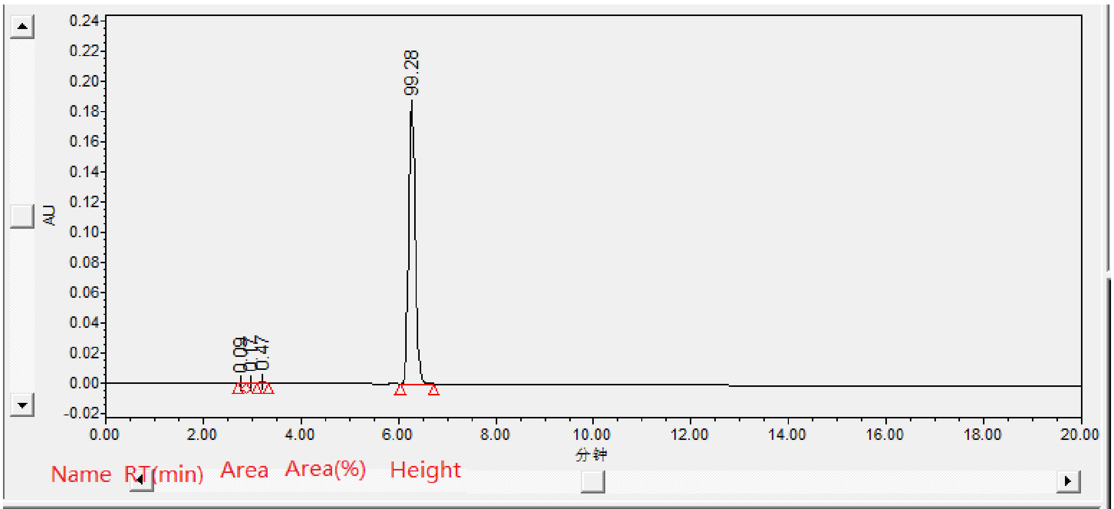

	名称	保留时间 (分钟)	面积 (微伏*秒)	% 面积	高度 (微伏)
1		2.770	1483	0.09	261
2		2.977	2857	0.17	353
3		3.206	8157	0.47	1432
4		6.269	1712113	99.28	185109

Figure SI 13-4. HPLC of compound **20.**

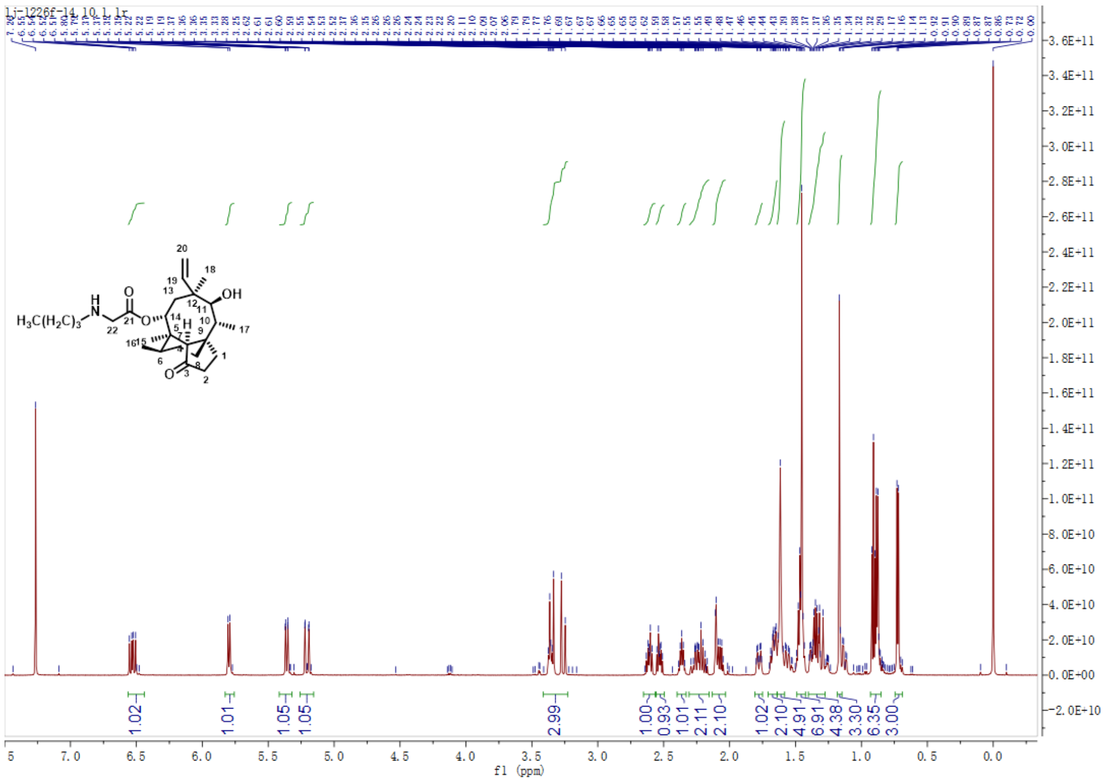

Figure SI 14-1. ^{1}H-NMR spectrum ($CDCl_3$, 400MHz) of compound **21**.

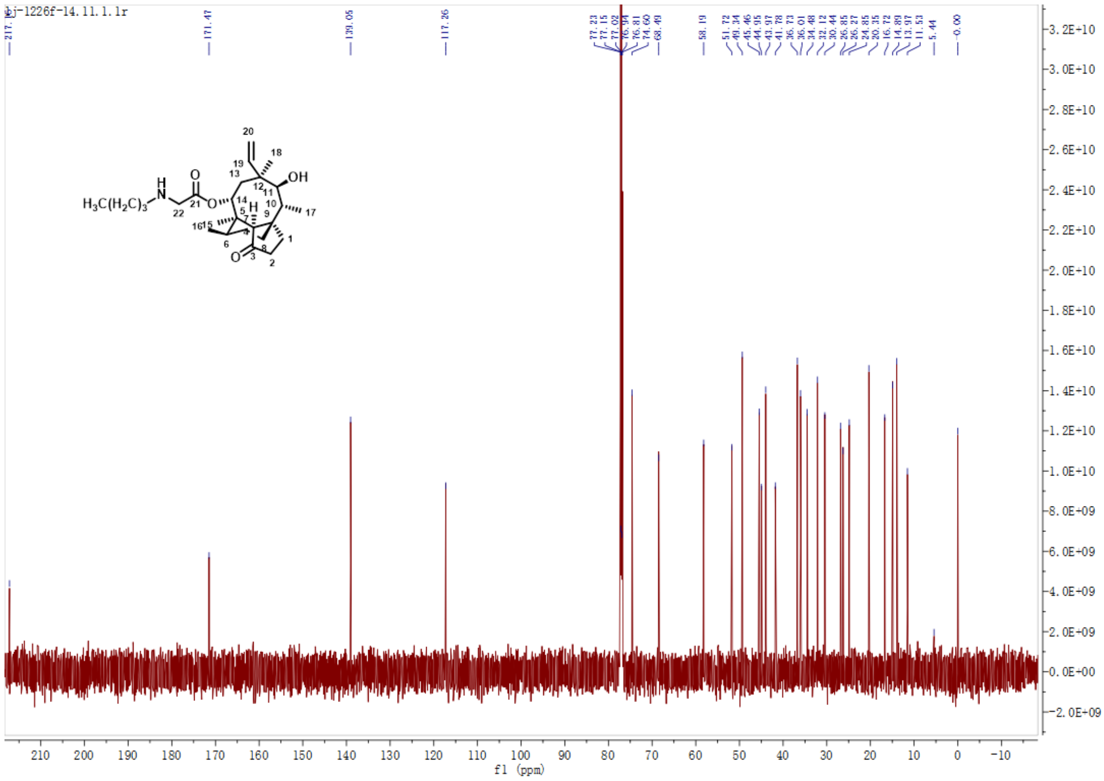

Figure SI 14-2. ^{13}C-NMR spectrum ($CDCl_3$, 101MHz) of compound **21**.

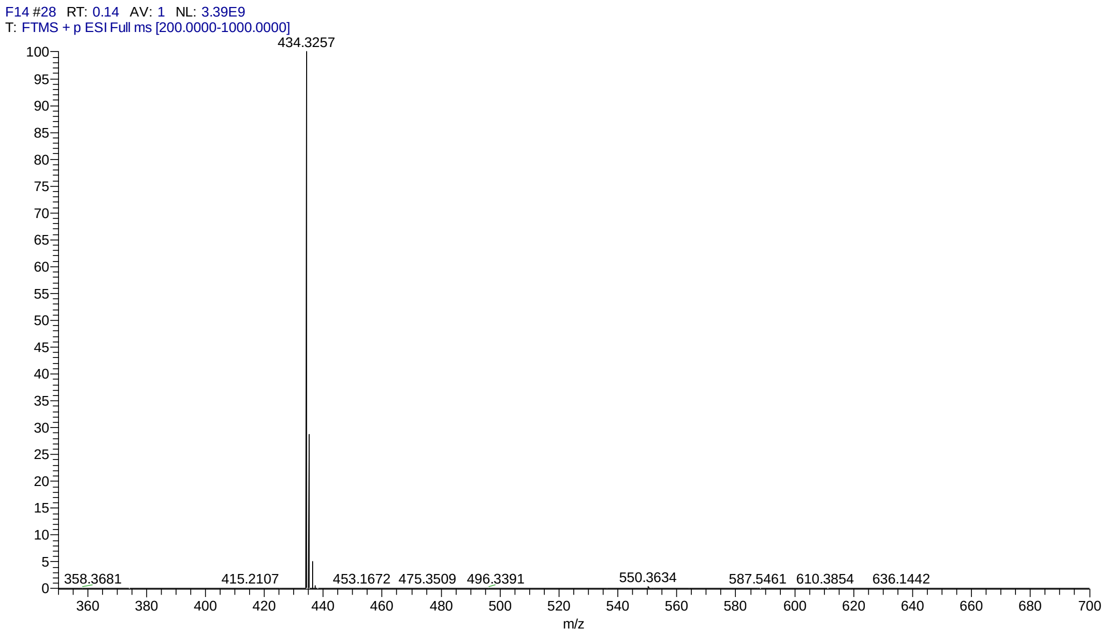

Figure SI 14-3. HR Mass spectrum (ESI) of compound **21**.

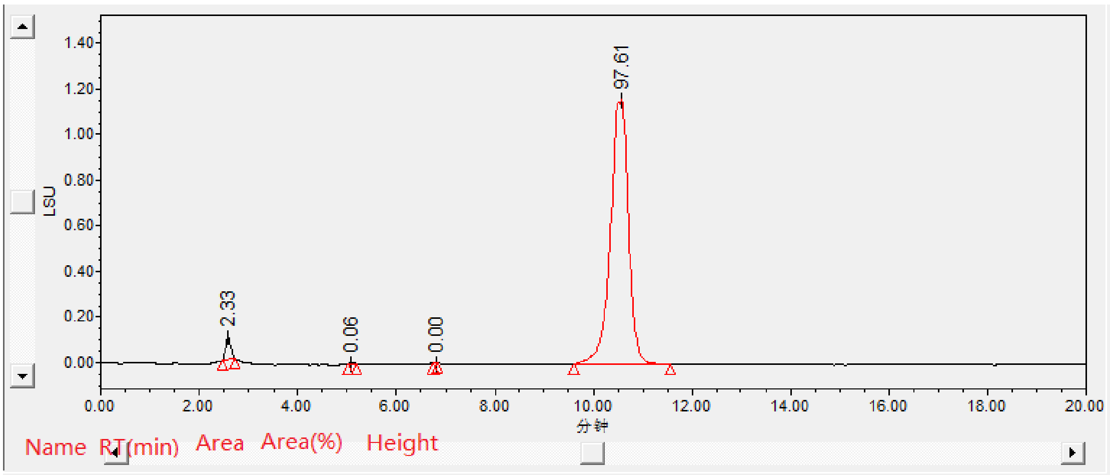

	名称	保留时间 (分钟)	面积 (微伏*秒)	% 面积	高度 (微伏)
1		2.580	708	2.33	100
2		5.079	18	0.06	3
3		6.813	1	0.00	0
4		10.576	29638	97.61	1164

Figure SI 14-4. HPLC of compound **21.**

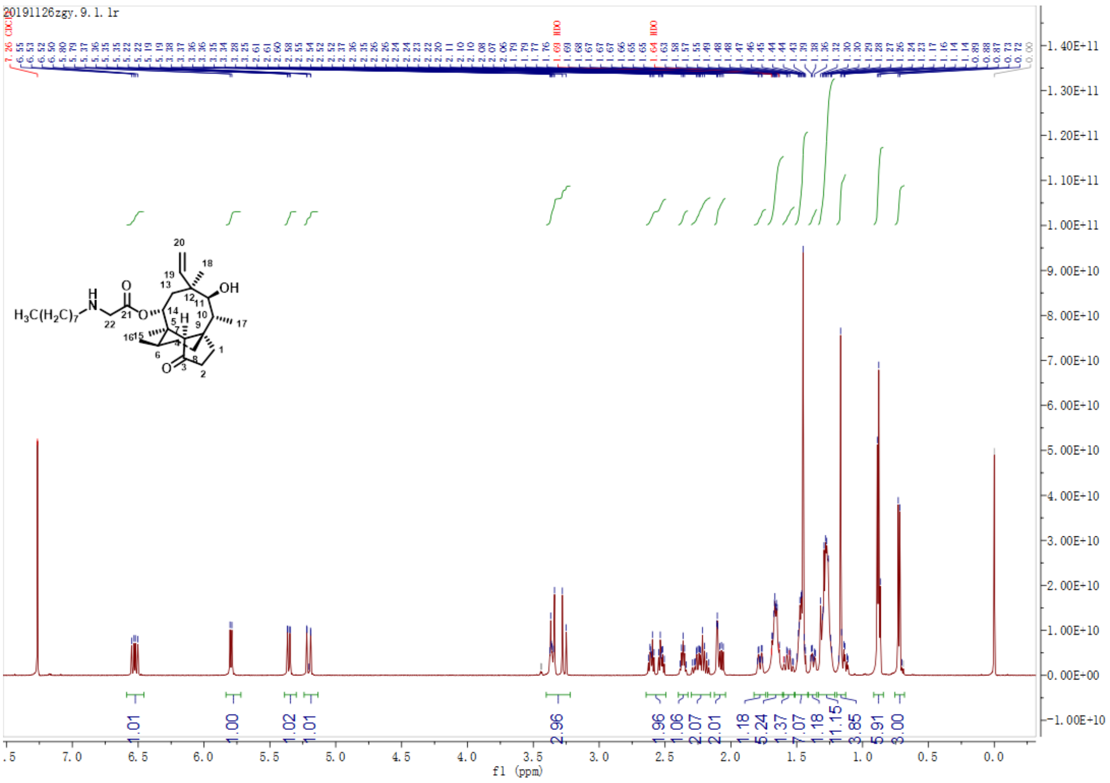

Figure SI 15-1. ^{1}H-NMR spectrum ($CDCl_3$, 400MHz) of compound **22**.

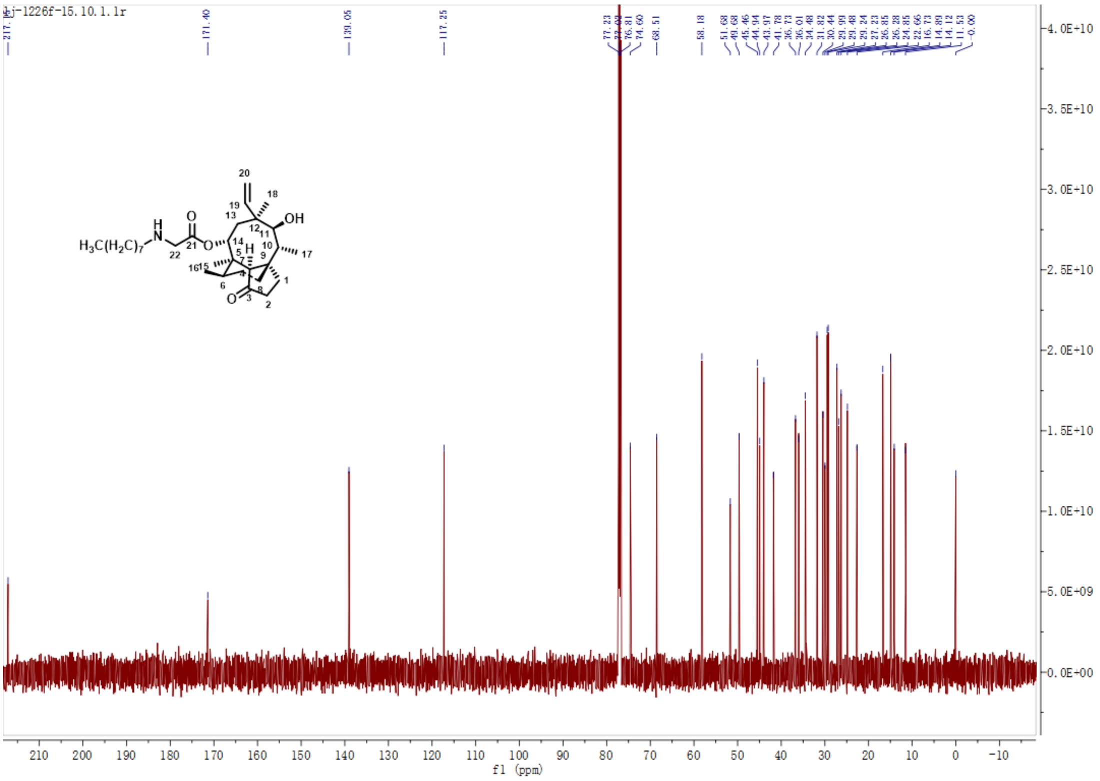

Figure SI 15-2. ^{13}C-NMR spectrum ($CDCl_3$, 101MHz) of compound **22**.

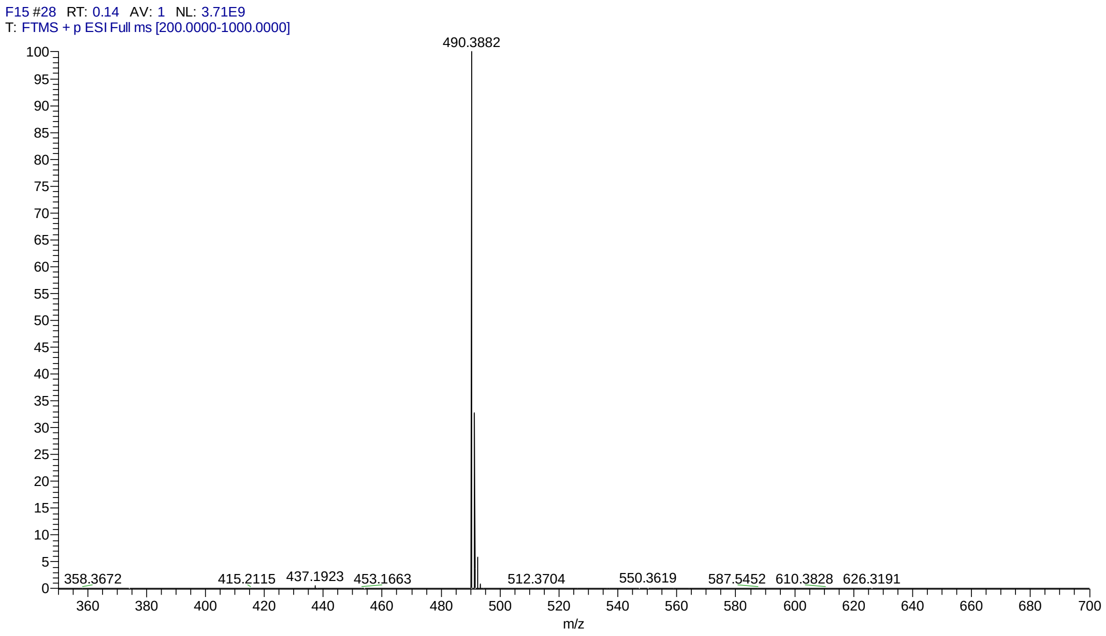

Figure SI 15-3. HR Mass spectrum (ESI) of compound **22**.

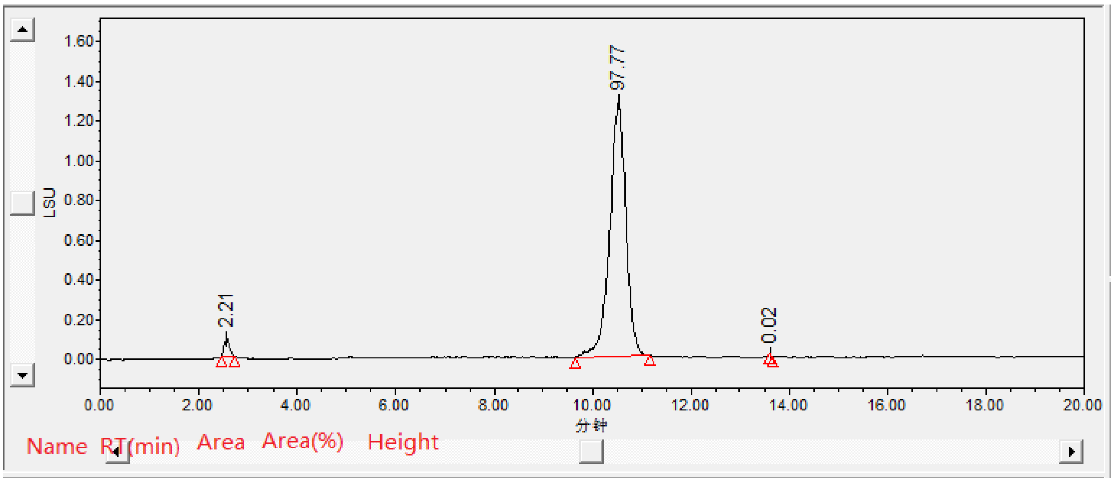

	名称	保留时间 (分钟)	面积 (微伏*秒)	% 面积	高度 (微伏)
1		2.556	661	2.21	91
2		10.534	29256	97.77	1282
3		13.617	7	0.02	4

Figure SI 15-4. HPLC of compound **22.**

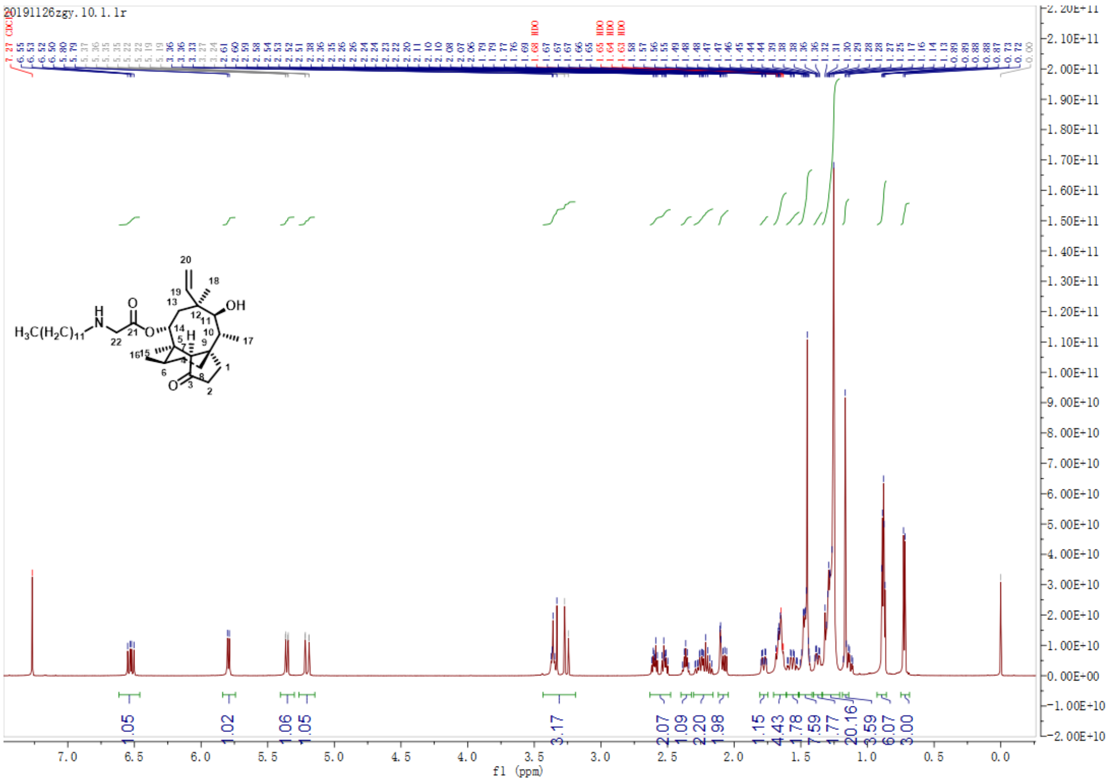

Figure SI 16-1. ^{1}H-NMR spectrum ($CDCl_3$, 400MHz) of compound **23**.

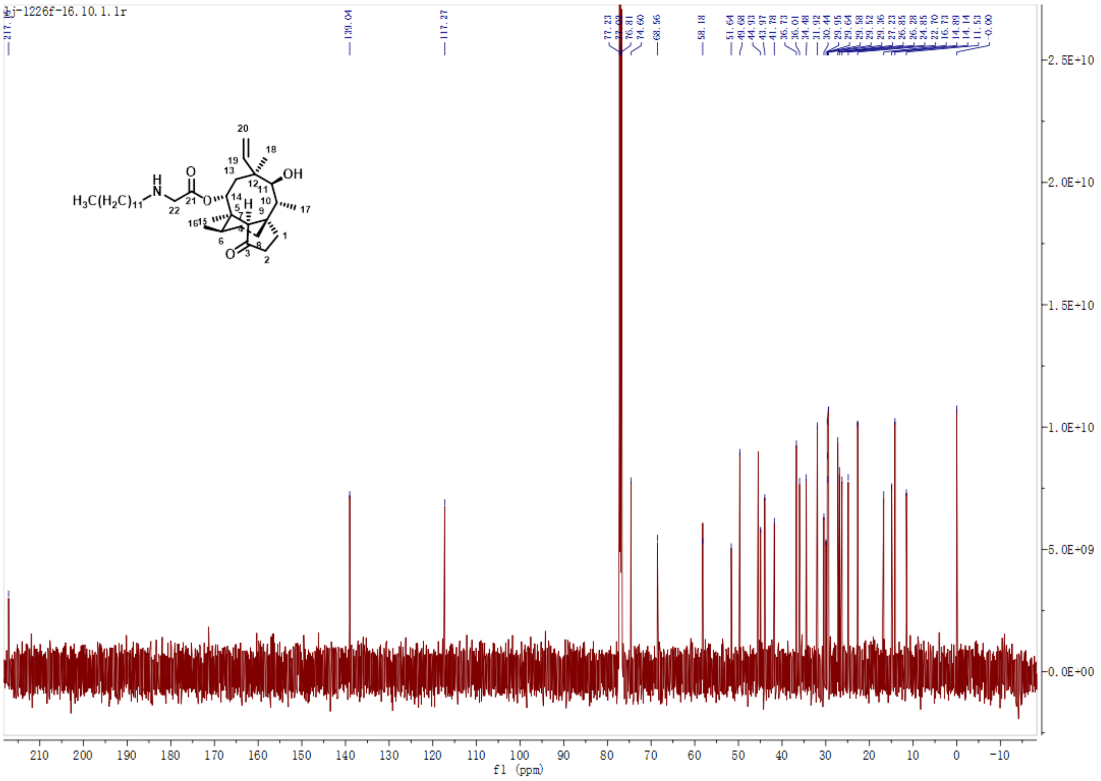

Figure SI 16-2. ^{13}C-NMR spectrum ($CDCl_3$, 101MHz) of compound **23**.

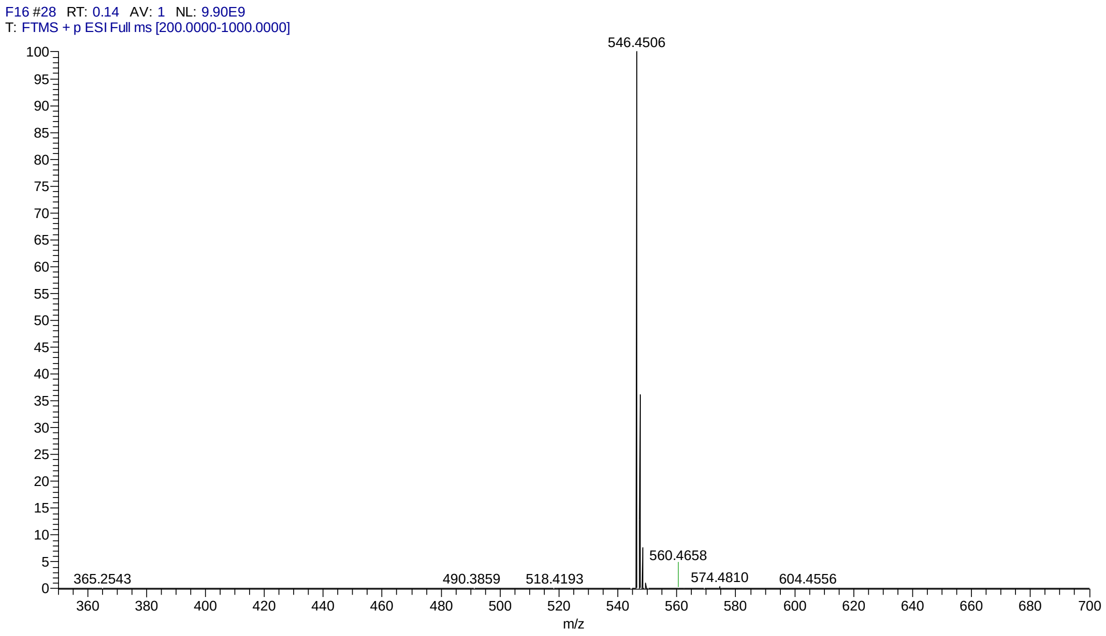

Figure SI 16-3. HR Mass spectrum (ESI) of compound **23**.

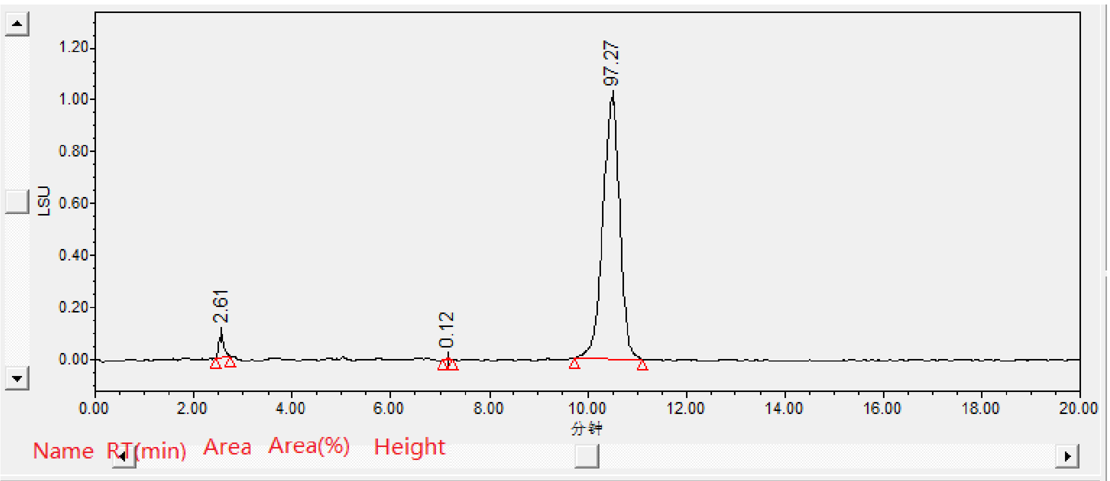

	名称	保留时间 (分钟)	面积 (微伏*秒)	% 面积	高度 (微伏)
1		2.551	649	2.61	89
2		7.156	29	0.12	6
3		10.502	24178	97.27	1009

Figure SI 16-4. HPLC of compound **23.**

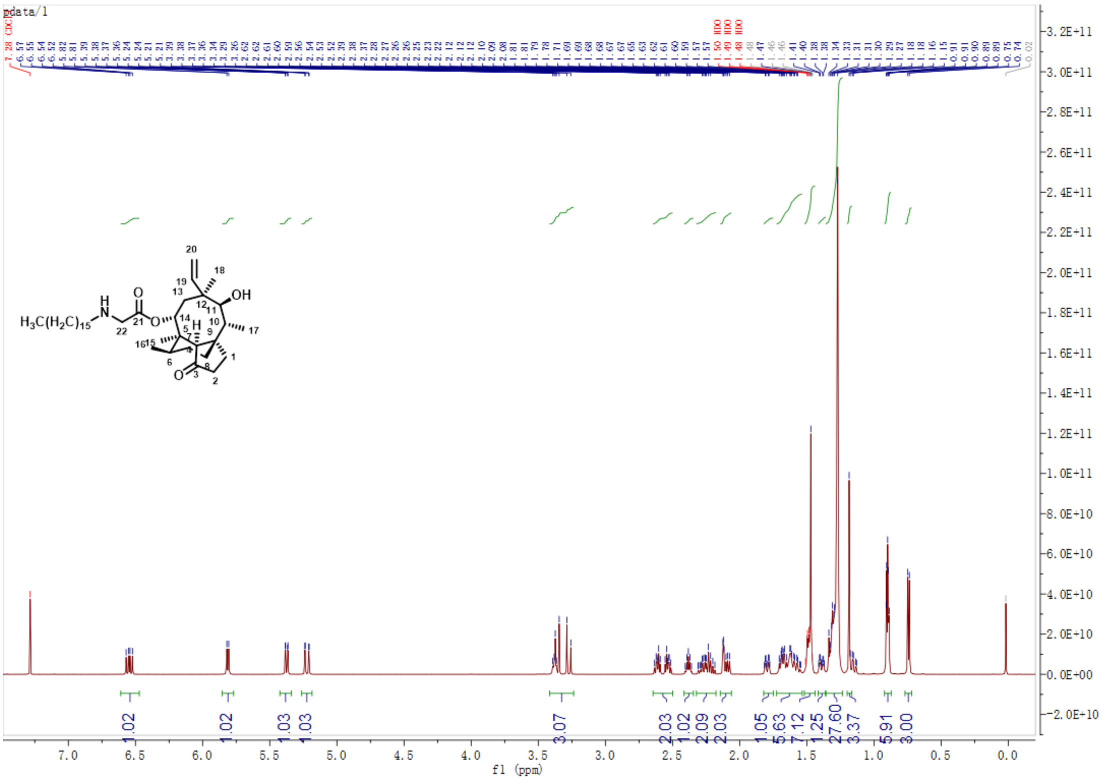

Figure SI 17-1. ^{1}H-NMR spectrum ($CDCl_3$, 400MHz) of compound **24**.

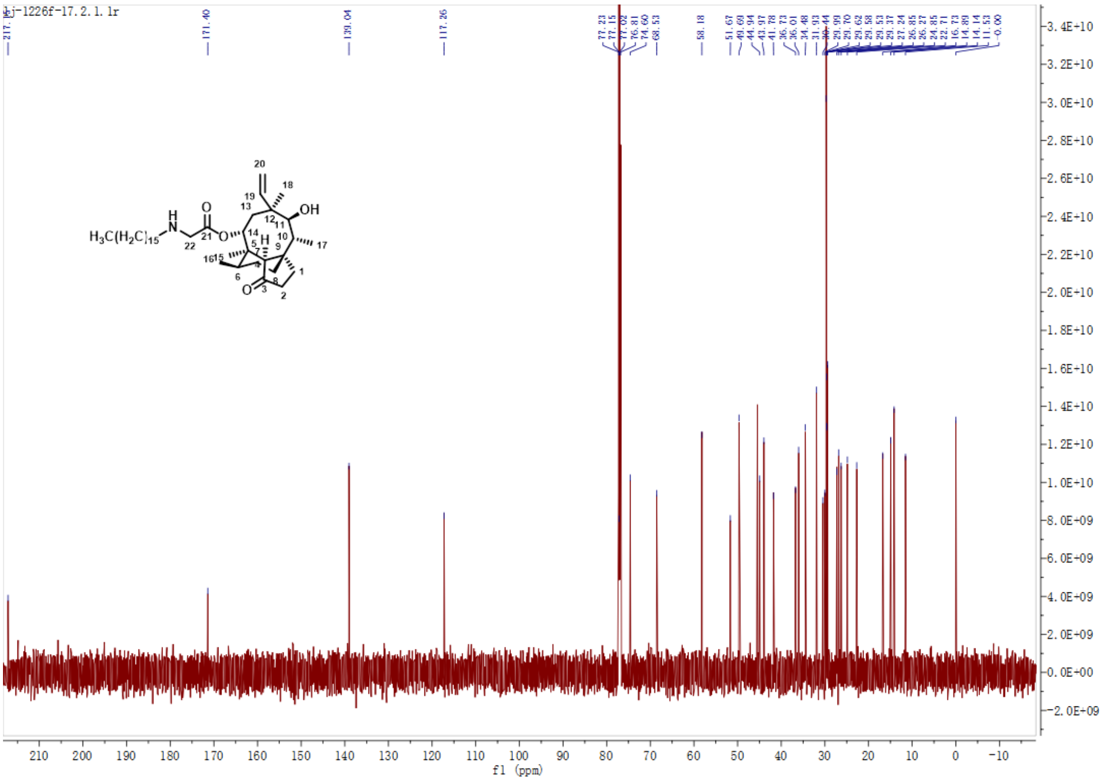

Figure SI 17-2. ^{13}C-NMR spectrum ($CDCl_3$, 101MHz) of compound **24**.

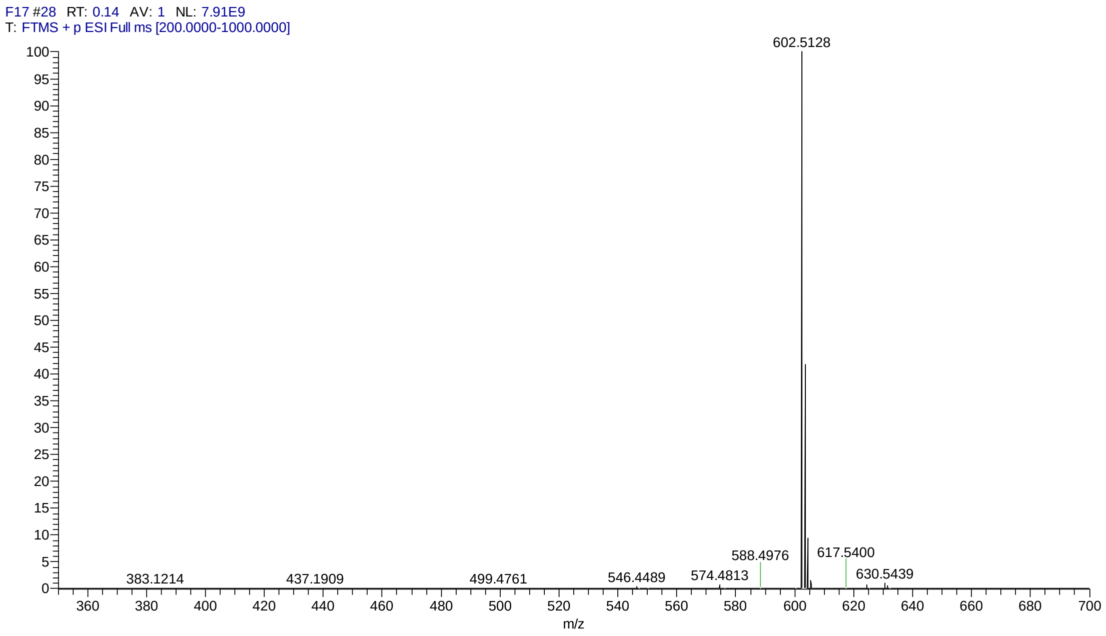

Figure SI 17-3. HR Mass spectrum (ESI) of compound **24**.

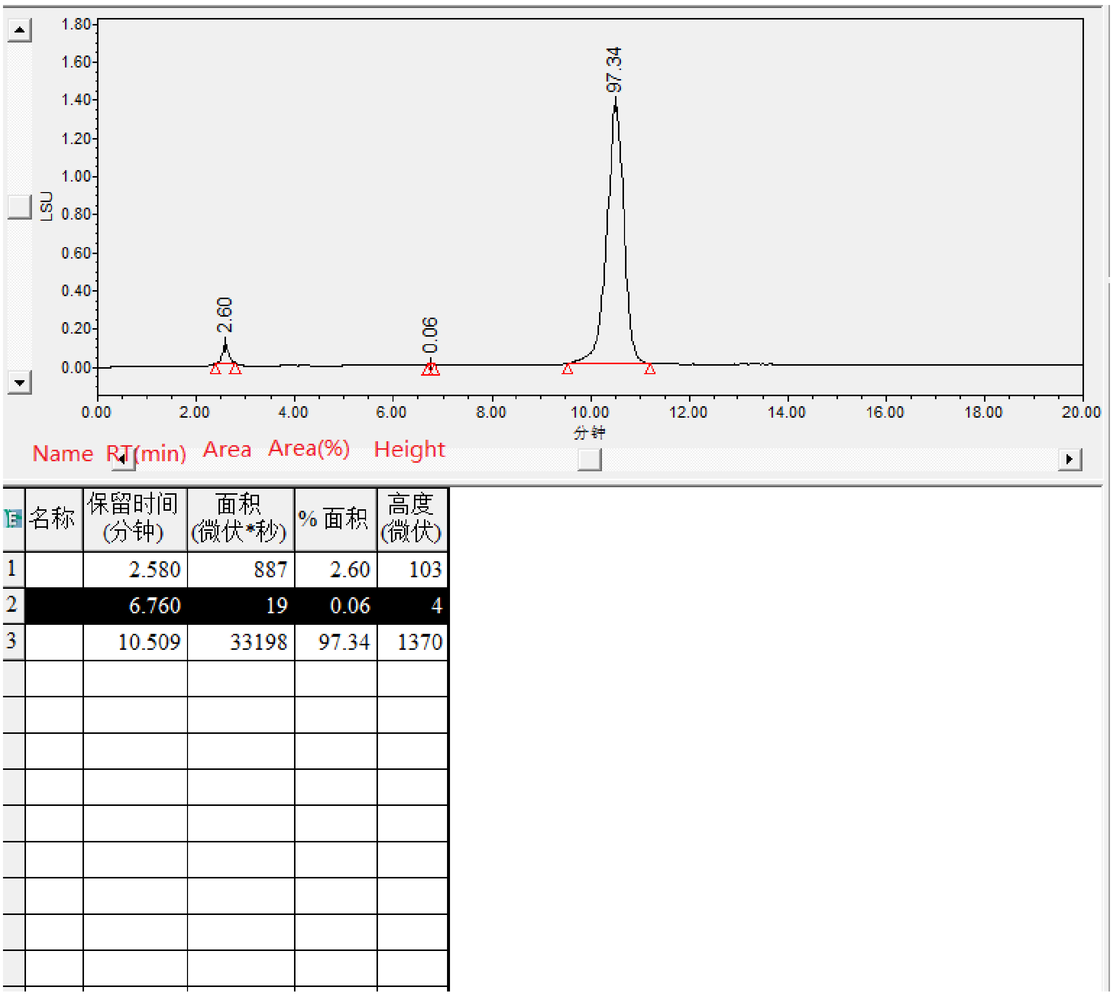

	名称	保留时间 (分钟)	面积 (微伏*秒)	% 面积	高度 (微伏)
1		2.580	887	2.60	103
2		6.760	19	0.06	4
3		10.509	33198	97.34	1370

Figure SI 17-4. HPLC of compound **24.**

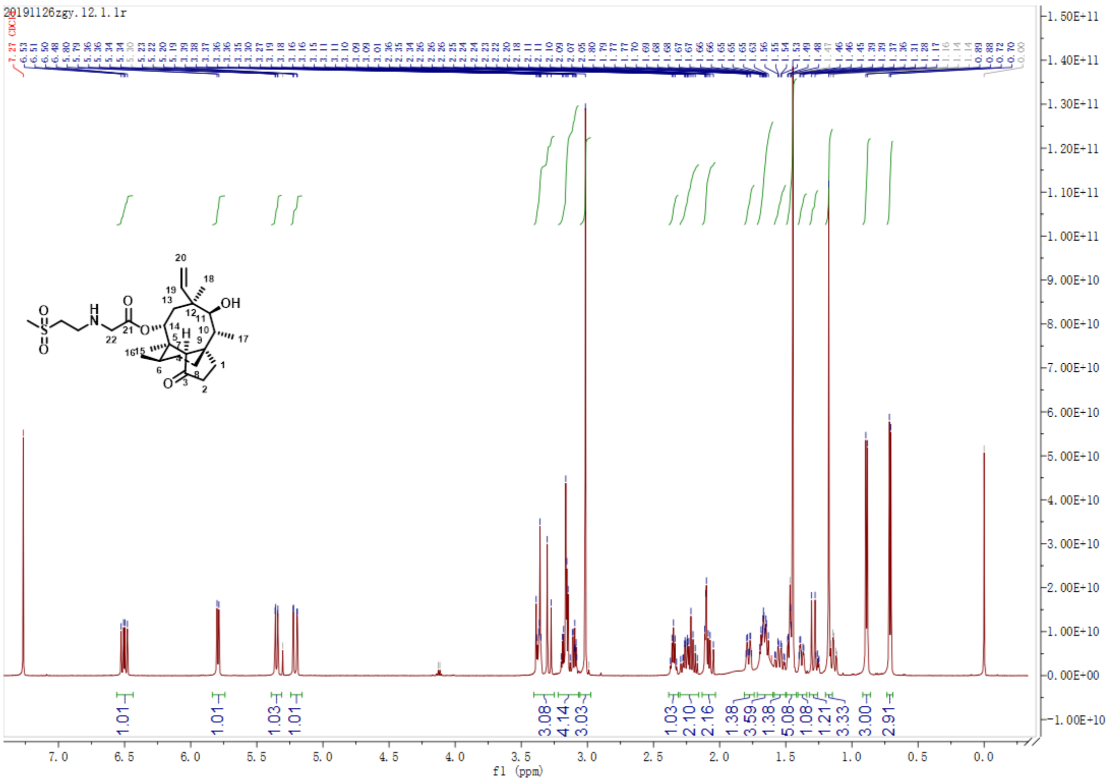

Figure SI 18-1. ^{1}H-NMR spectrum ($CDCl_3$, 400MHz) of compound **25**.

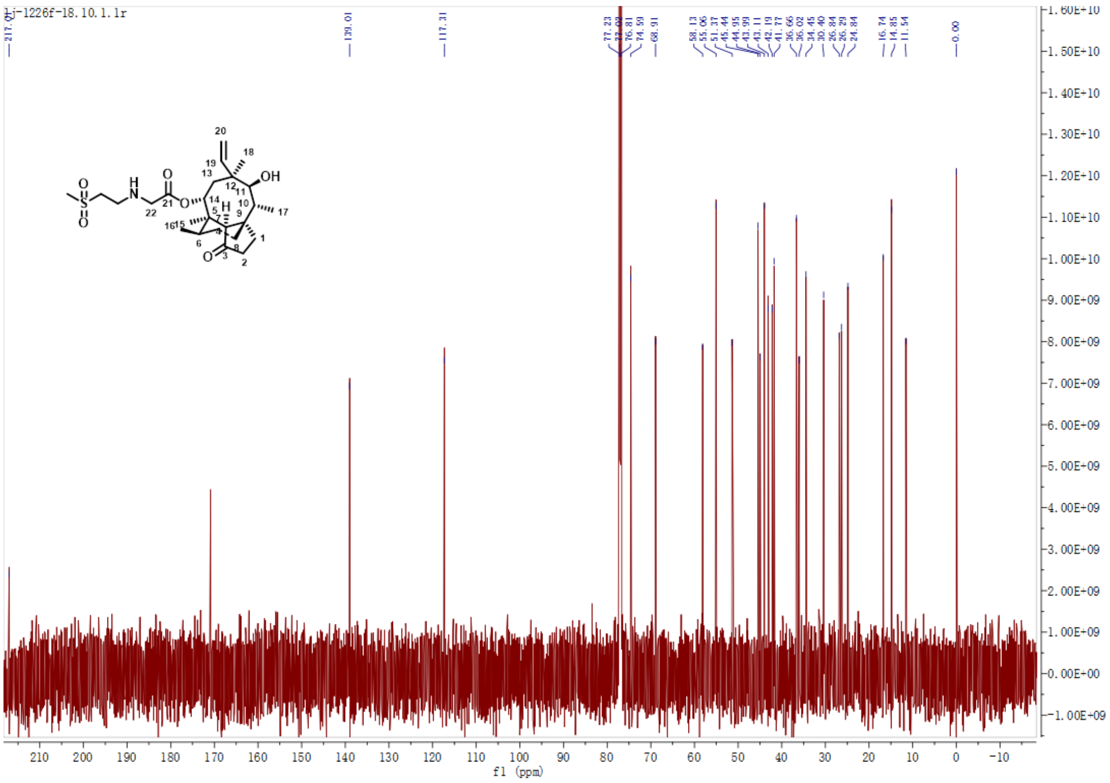

Figure SI 18-2. ^{13}C-NMR spectrum ($CDCl_3$, 101MHz) of compound **25**.

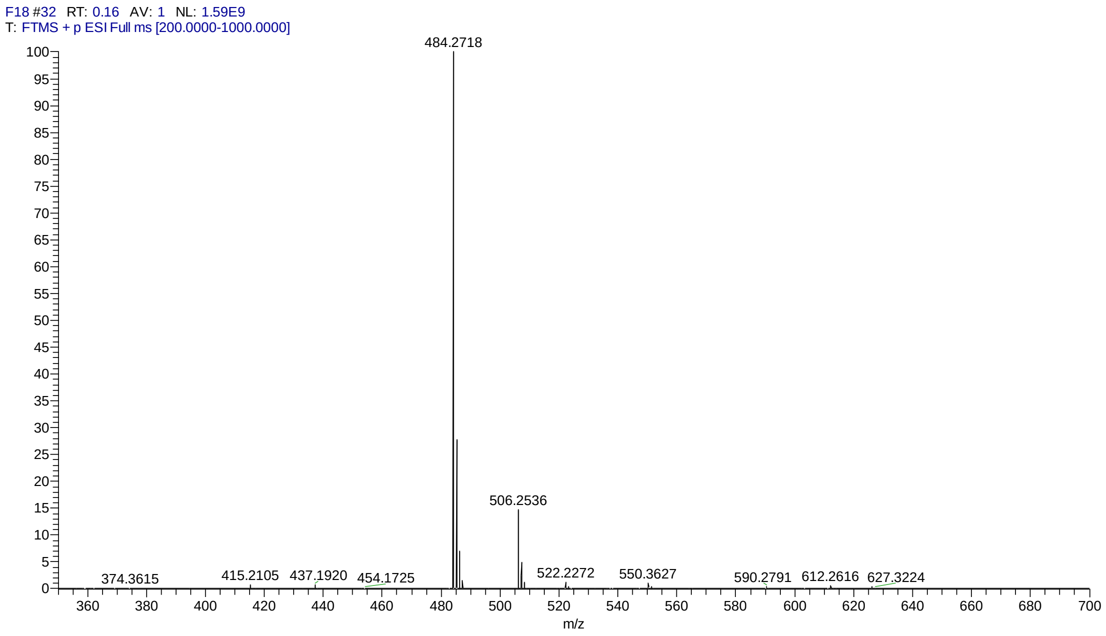

Figure SI 18-3. HR Mass spectrum (ESI) of compound **25**.

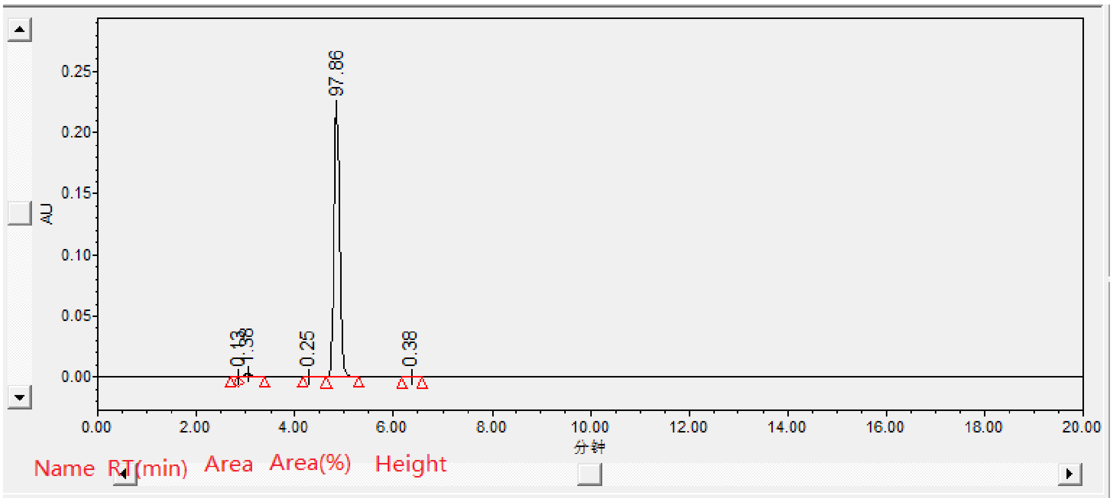

	名称	保留时间 (分钟)	面积 (微伏*秒)	% 面积	高度 (微伏)
1		2.850	2304	0.13	349
2		3.032	25199	1.38	2660
3		4.274	4546	0.25	501
4		4.839	1786471	97.86	223151
5		6.366	6976	0.38	733

Figure SI 18-4. HPLC of compound **25.**

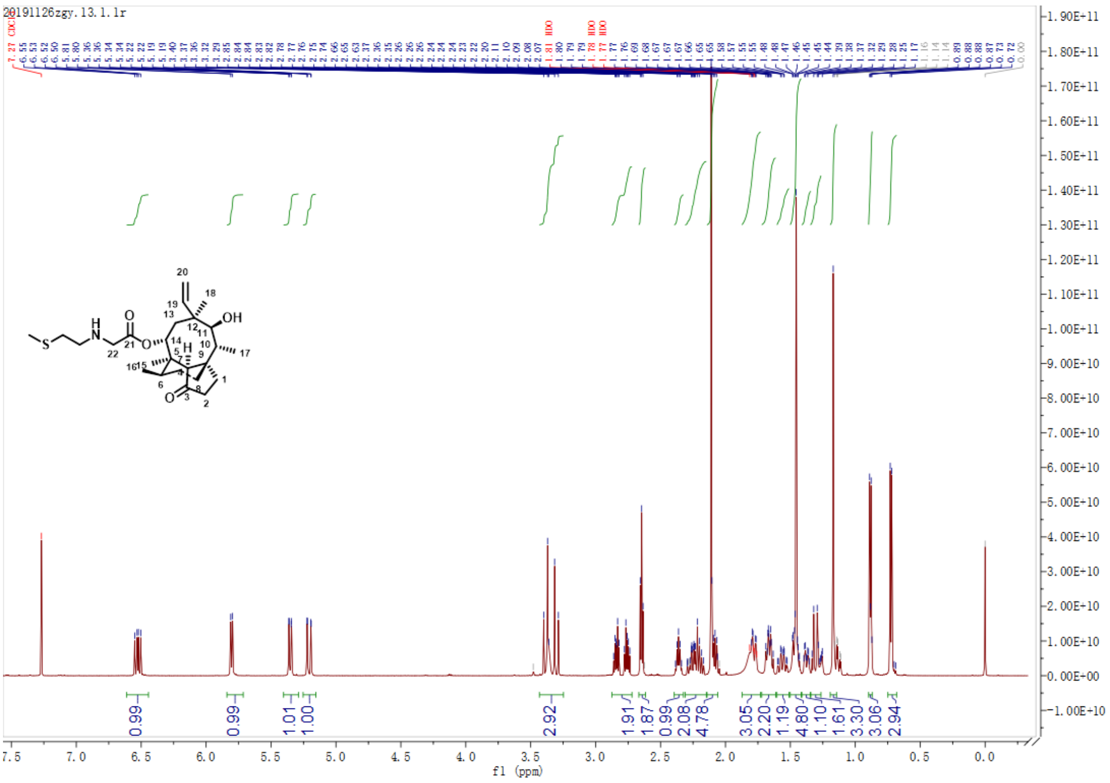

Figure SI 19-1. ^{1}H-NMR spectrum ($CDCl_3$, 400MHz) of compound **26**.

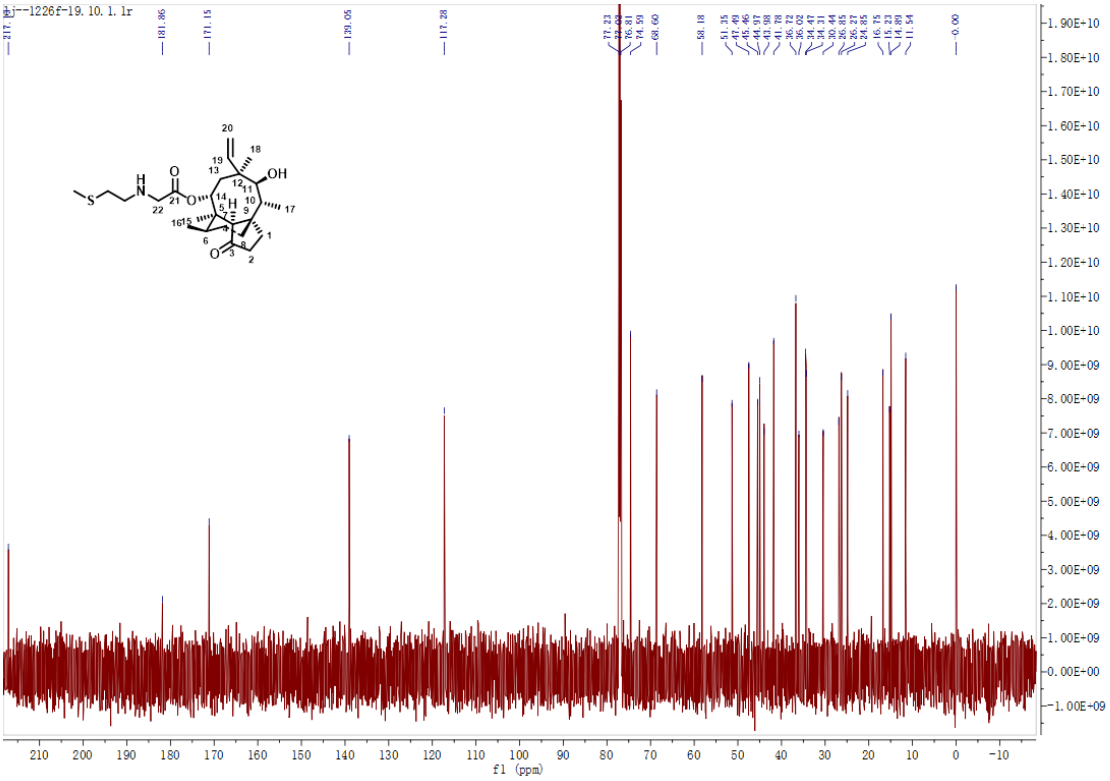

Figure SI 19-2. ^{13}C-NMR spectrum ($CDCl_3$, 101MHz) of compound **26**.

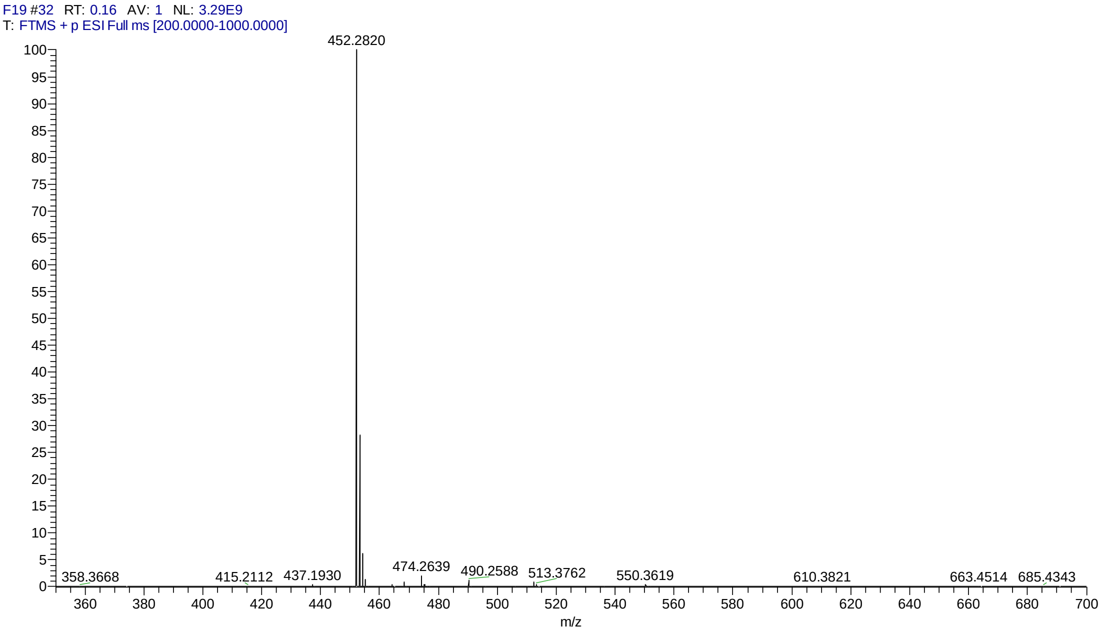

Figure SI 19-3. HR Mass spectrum (ESI) of compound **26**.

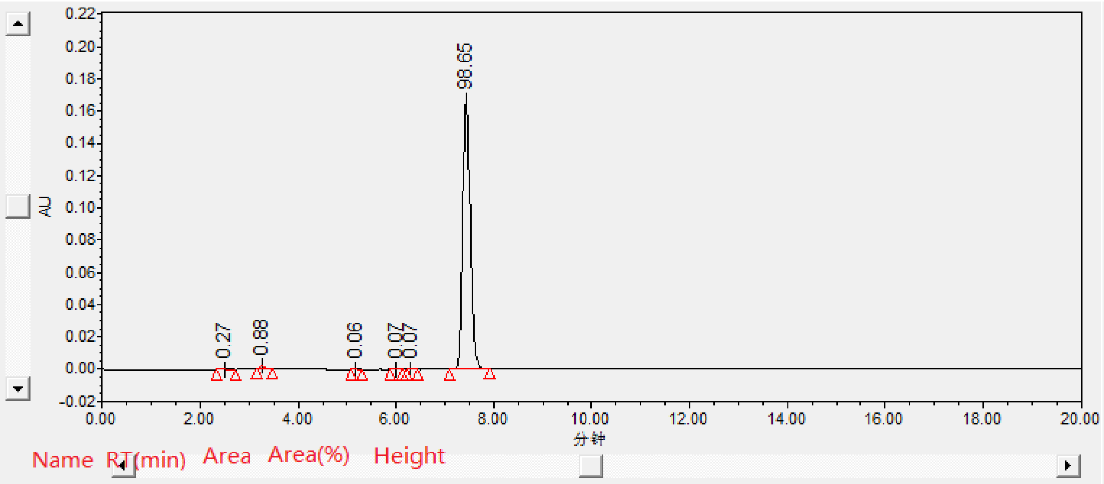

	名称	保留时间 (分钟)	面积 (微伏*秒)	% 面积	高度 (微伏)
1		2.492	4965	0.27	324
2		3.257	15942	0.88	2290
3		5.170	1009	0.06	146
4		5.994	1343	0.07	163
5		6.300	1324	0.07	179
6		7.436	1794042	98.65	167498

Figure SI 19-4. HPLC of compound **26.**

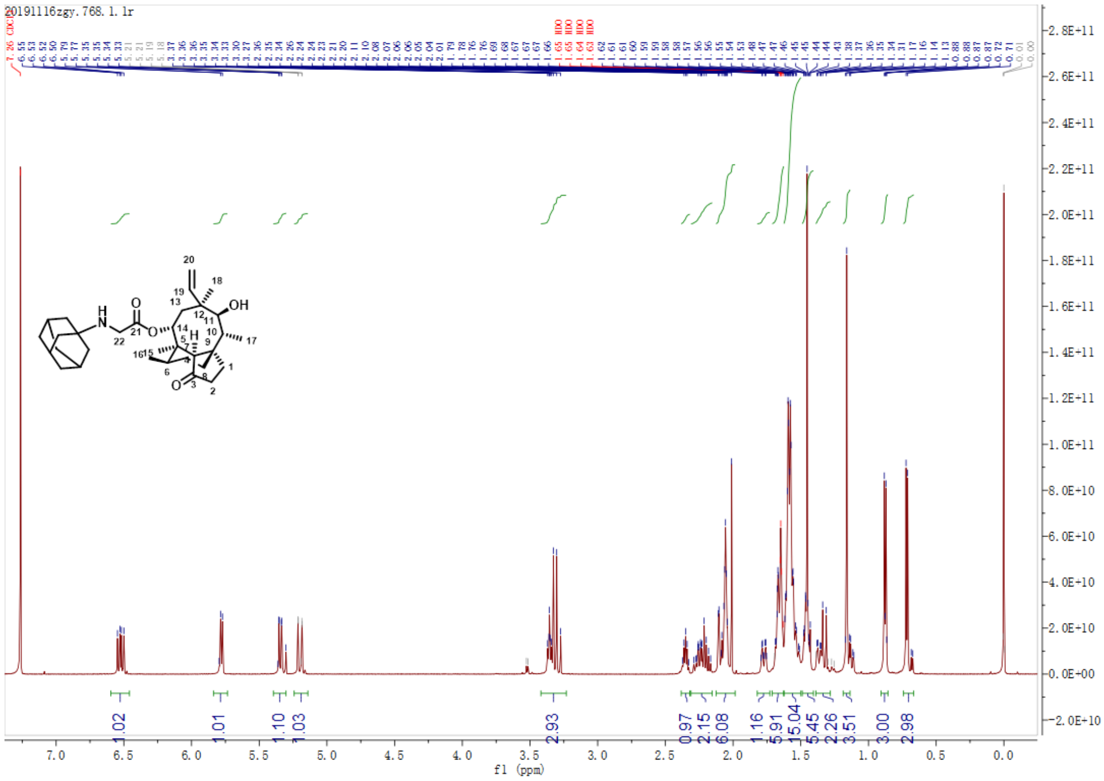

Figure SI 20-1. ^{1}H-NMR spectrum ($CDCl_3$, 400MHz) of compound **27**.

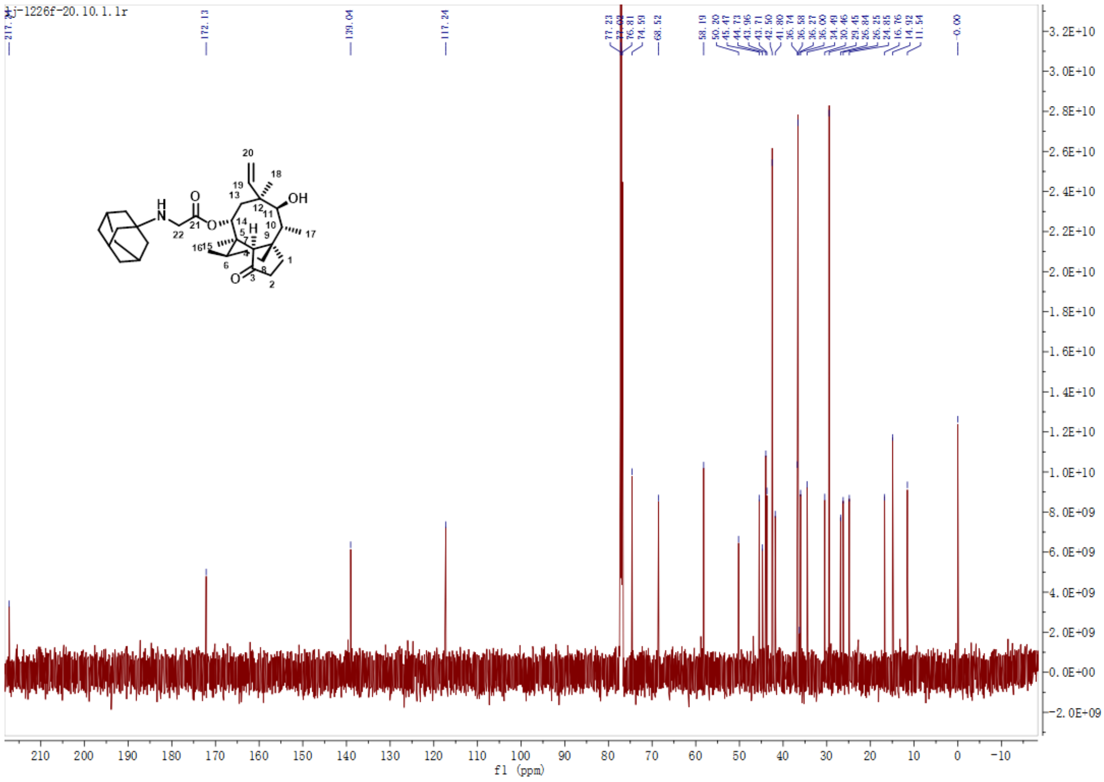

Figure SI 20-2. ^{13}C-NMR spectrum ($CDCl_3$, 101MHz) of compound **27**.

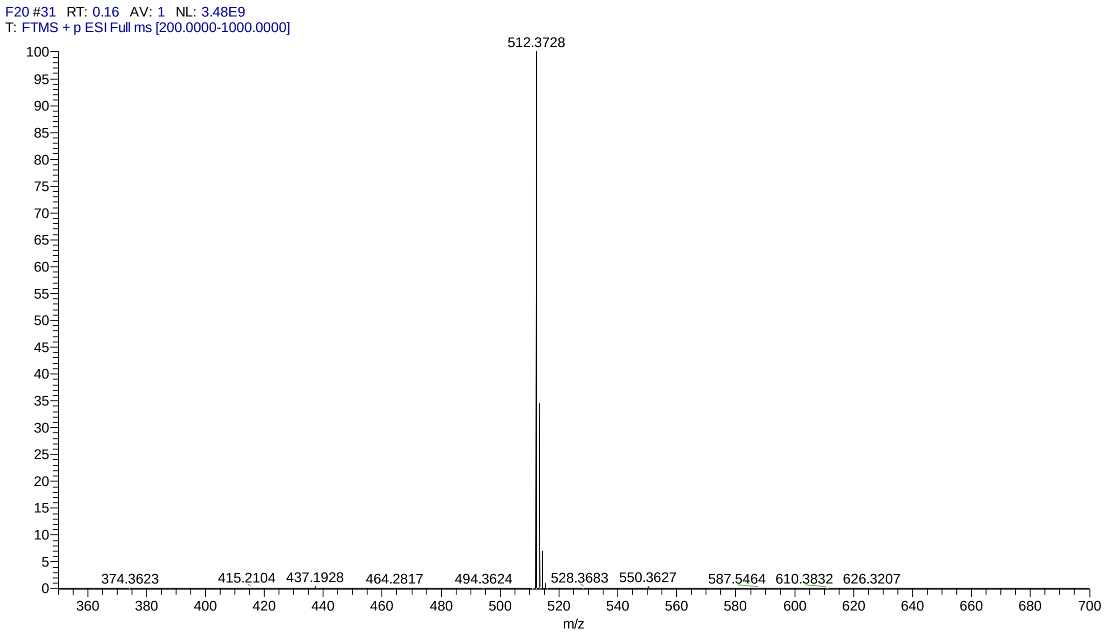

Figure SI 20-3. HR Mass spectrum (ESI) of compound **27**.

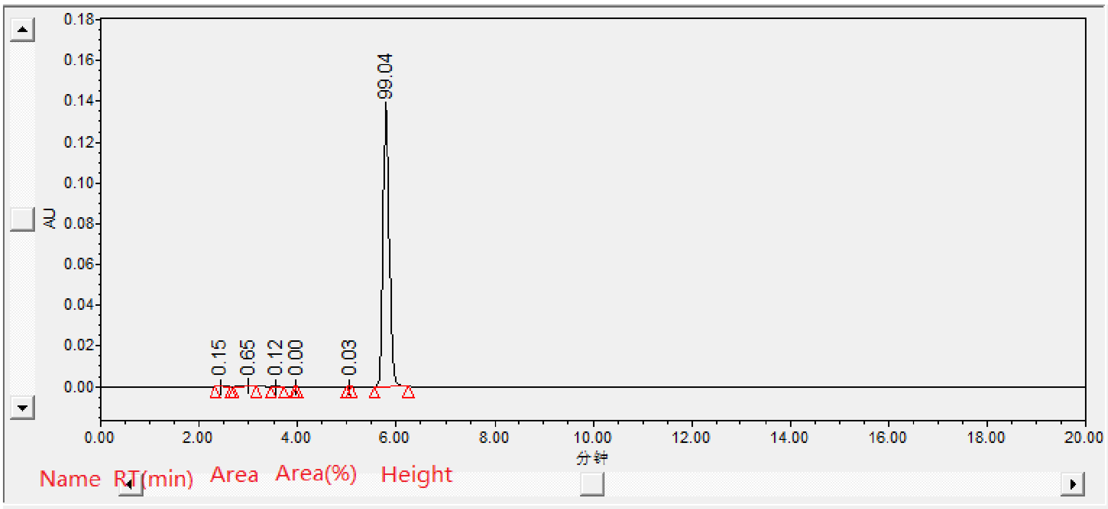

	名称	保留时间 (分钟)	面积 (微伏*秒)	% 面积	高度 (微伏)
1		2.414	1885	0.15	184
2		2.989	8147	0.65	503
3		3.549	1543	0.12	236
4		3.967	27	0.00	14
5		5.057	342	0.03	76
6		5.789	1234048	99.04	137265

Figure SI 20-4. HPLC of compound **27.**

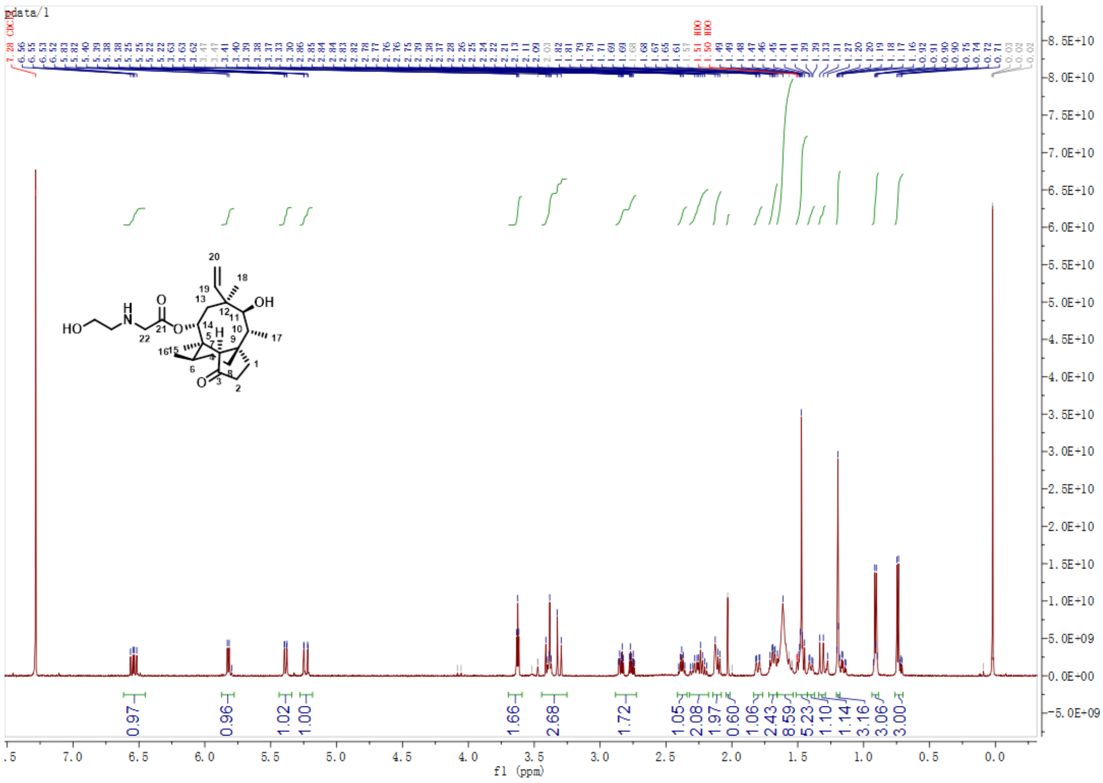

Figure SI 21-1. ^{1}H-NMR spectrum ($CDCl_3$, 400MHz) of compound **28**.

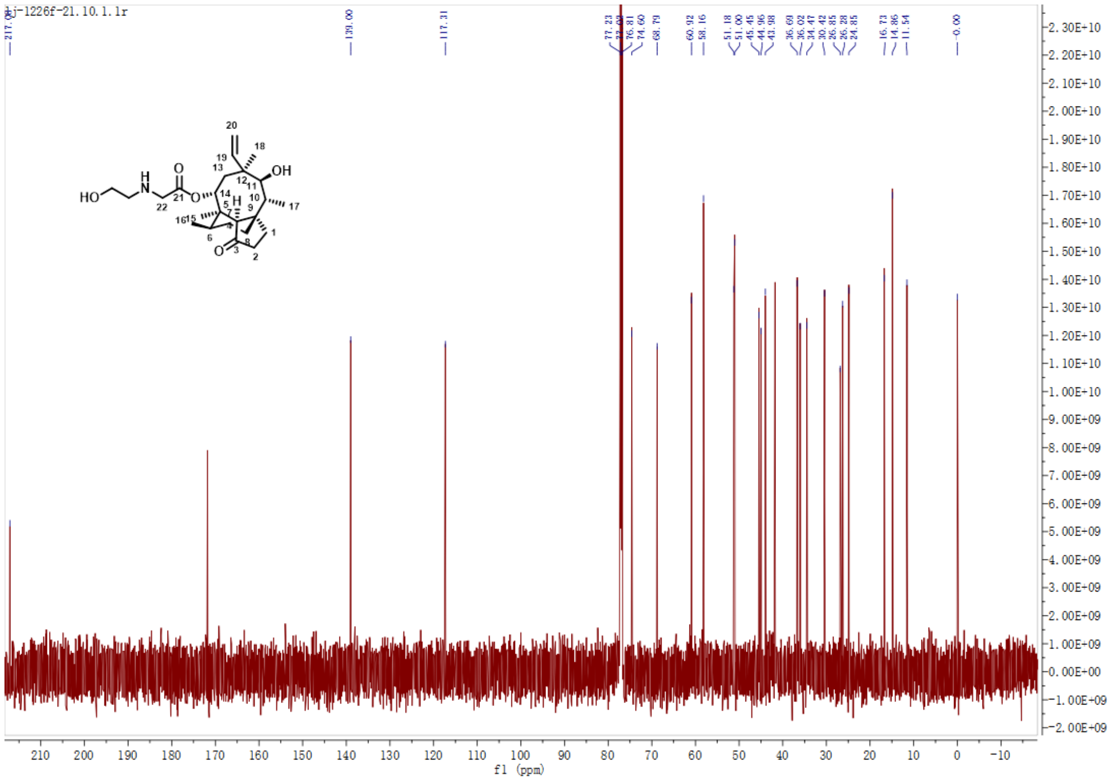

Figure SI 21-2. ^{13}C-NMR spectrum ($CDCl_3$, 101MHz) of compound **28**.

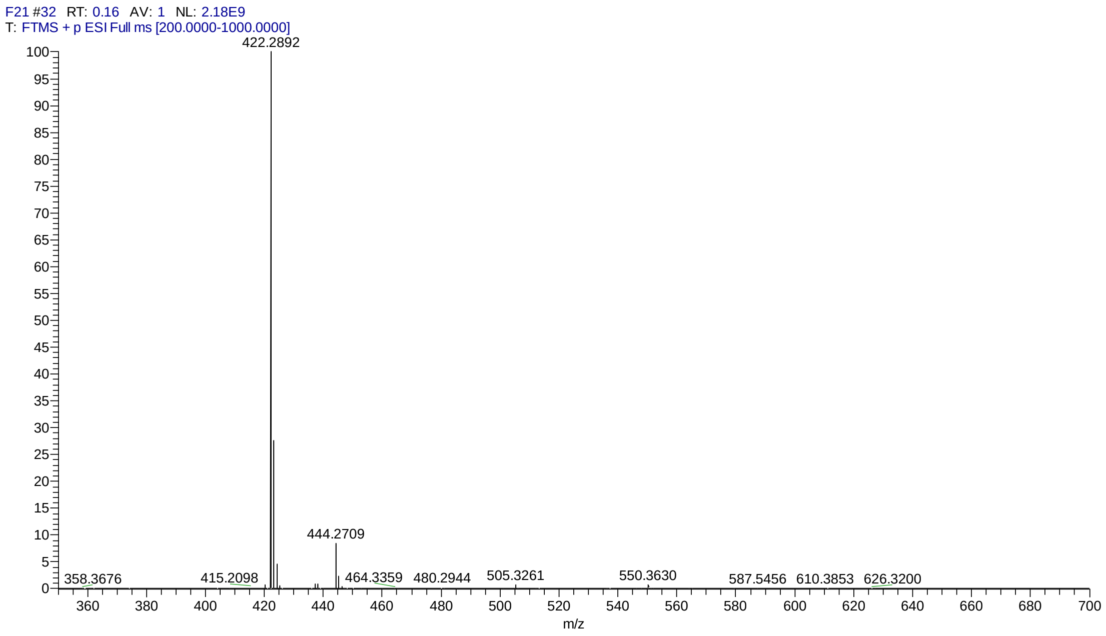

Figure SI 21-3. HR Mass spectrum (ESI) of compound **28**.

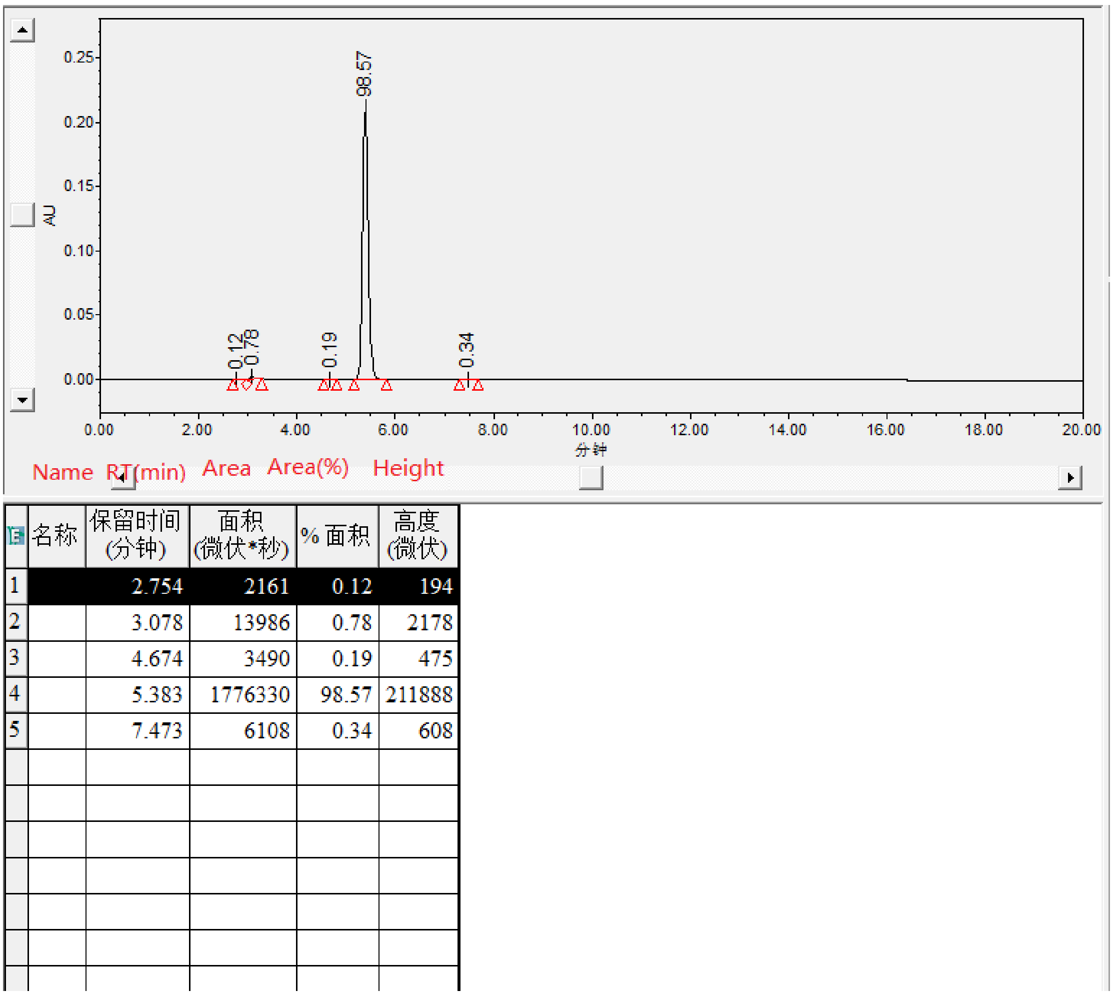

	名称	保留时间 (分钟)	面积 (微伏*秒)	% 面积	高度 (微伏)
1		2.754	2161	0.12	194
2		3.078	13986	0.78	2178
3		4.674	3490	0.19	475
4		5.383	1776330	98.57	211888
5		7.473	6108	0.34	608

Figure SI 21-4. HPLC of compound **28.**

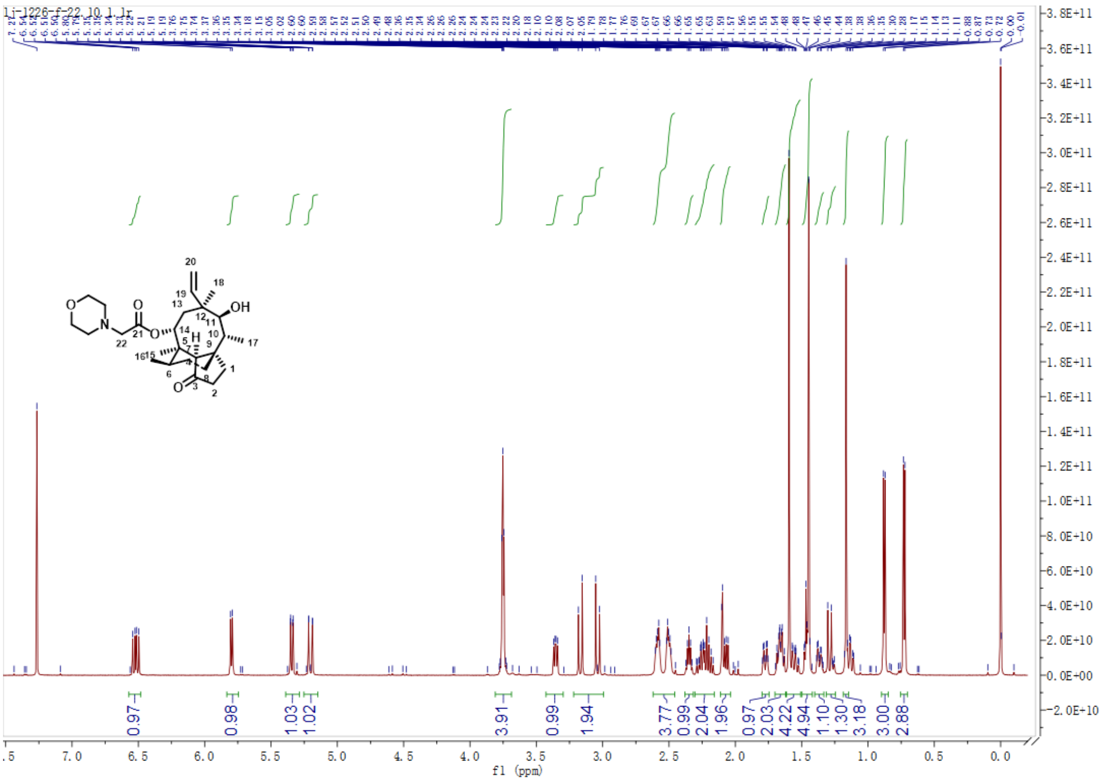

Figure SI 22-1. ^{1}H-NMR spectrum ($CDCl_3$, 400MHz) of compound **29**.

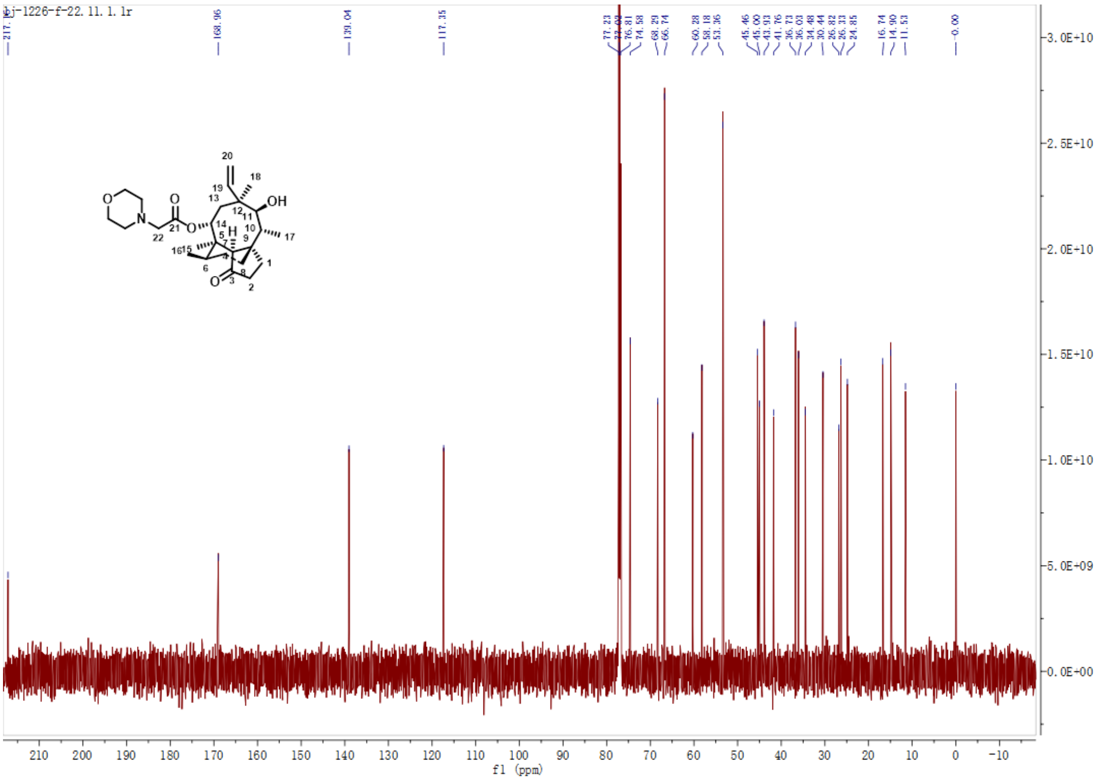

Figure SI 22-2. ^{13}C-NMR spectrum ($CDCl_3$, 101MHz) of compound **29**.

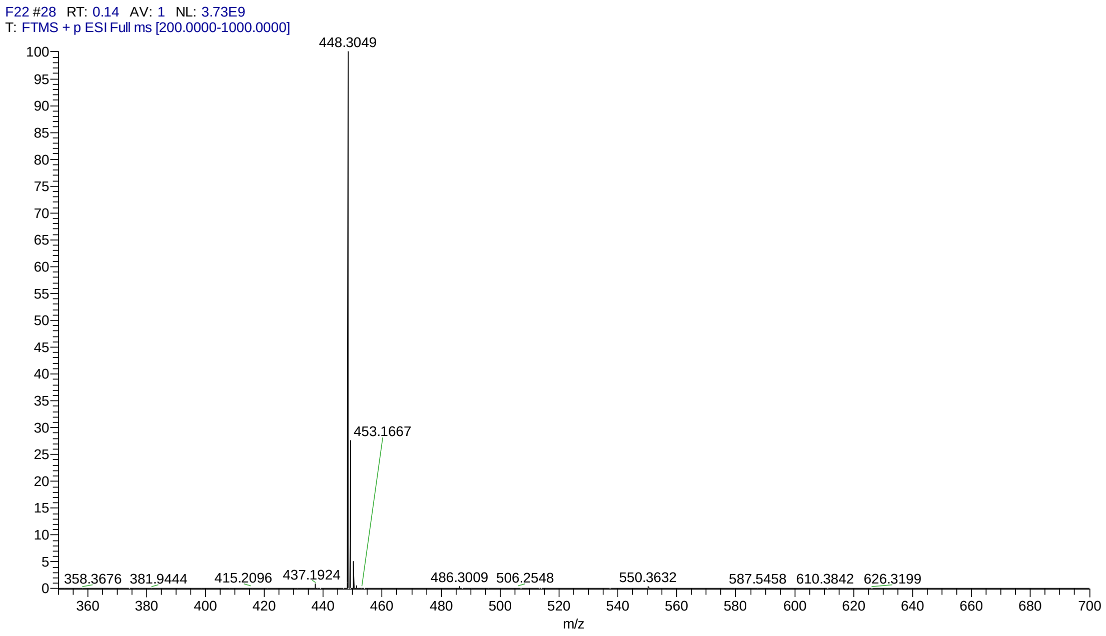

Figure SI 22-3. HR Mass spectrum (ESI) of compound **29**.

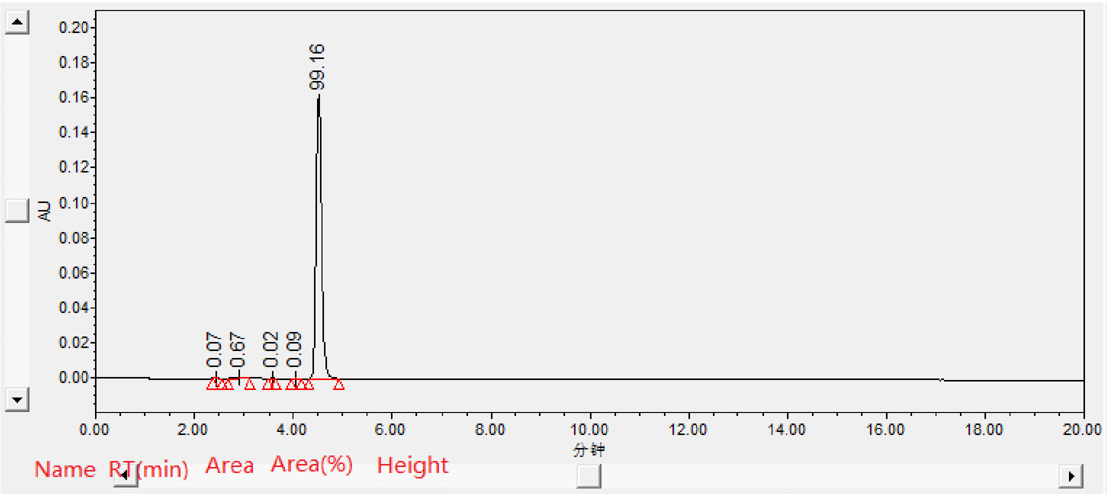

	名称	保留时间 (分钟)	面积 (微伏*秒)	% 面积	高度 (微伏)
1		2.424	855	0.07	115
2		2.893	8419	0.67	595
3		3.573	222	0.02	46
4		4.037	1187	0.09	186
5		4.507	1253631	99.16	160096

Figure SI 22-4. HPLC of compound **29.**

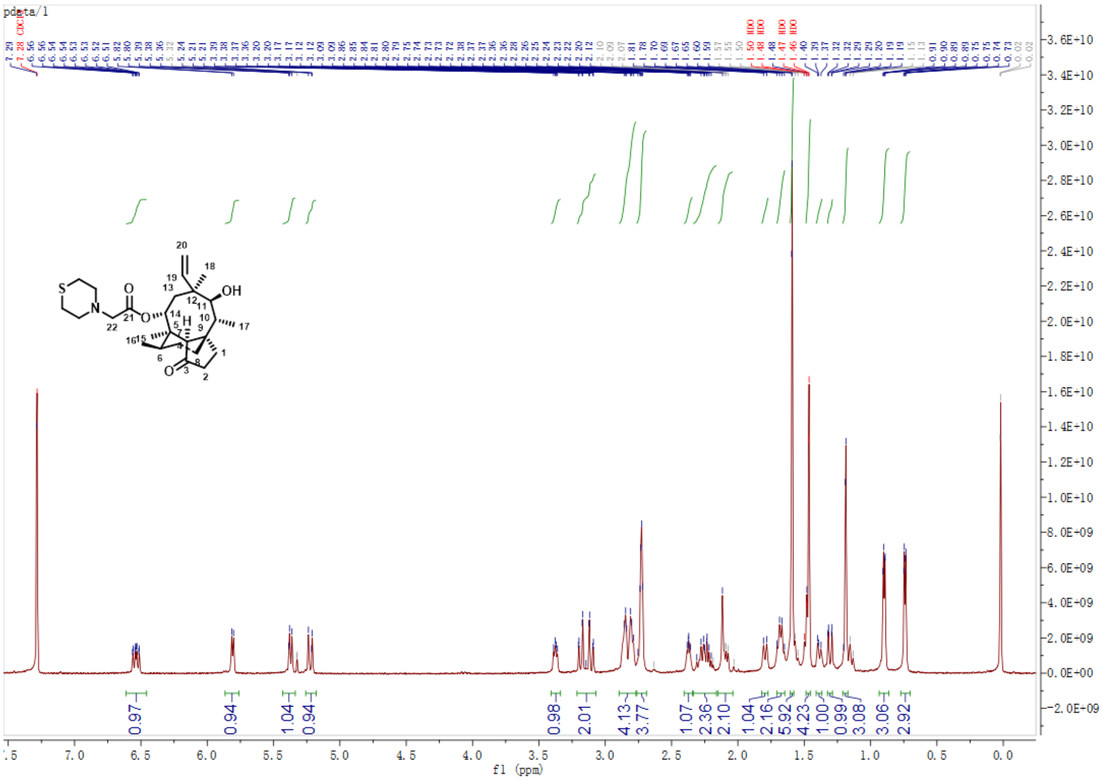

Figure SI 23-1. ^{1}H-NMR spectrum ($CDCl_3$, 400MHz) of compound **30**.

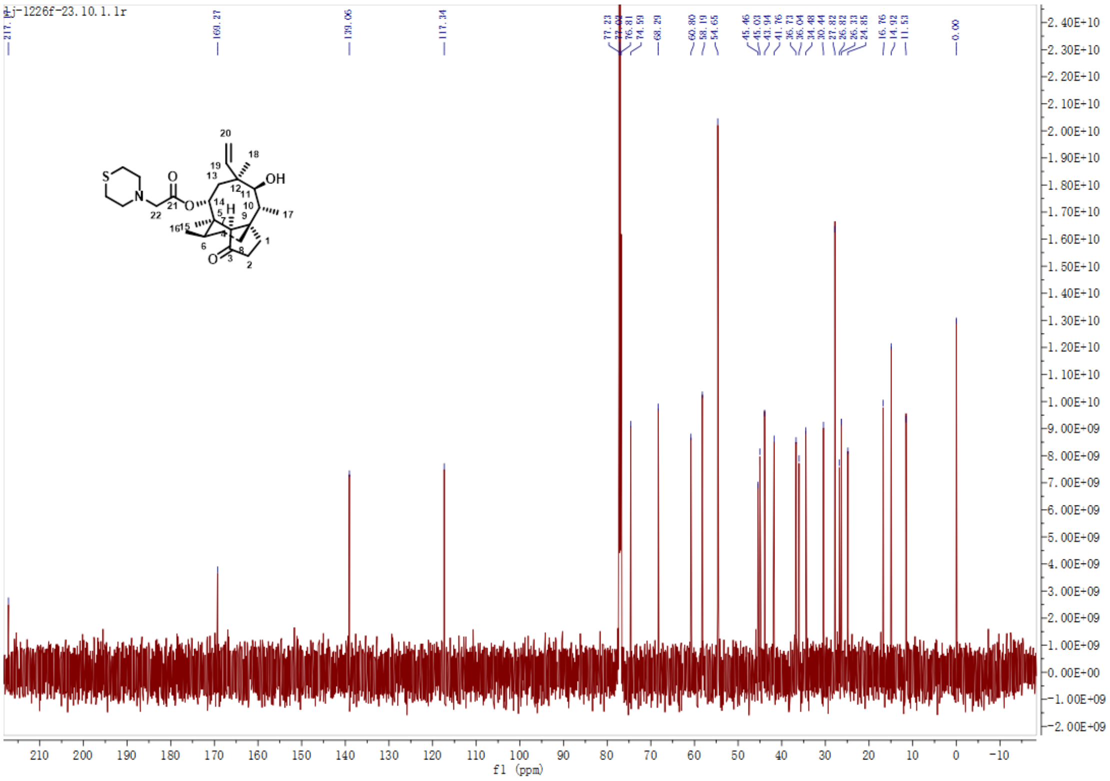

Figure SI 23-2. ^{13}C-NMR spectrum ($CDCl_3$, 101MHz) of compound **30**.

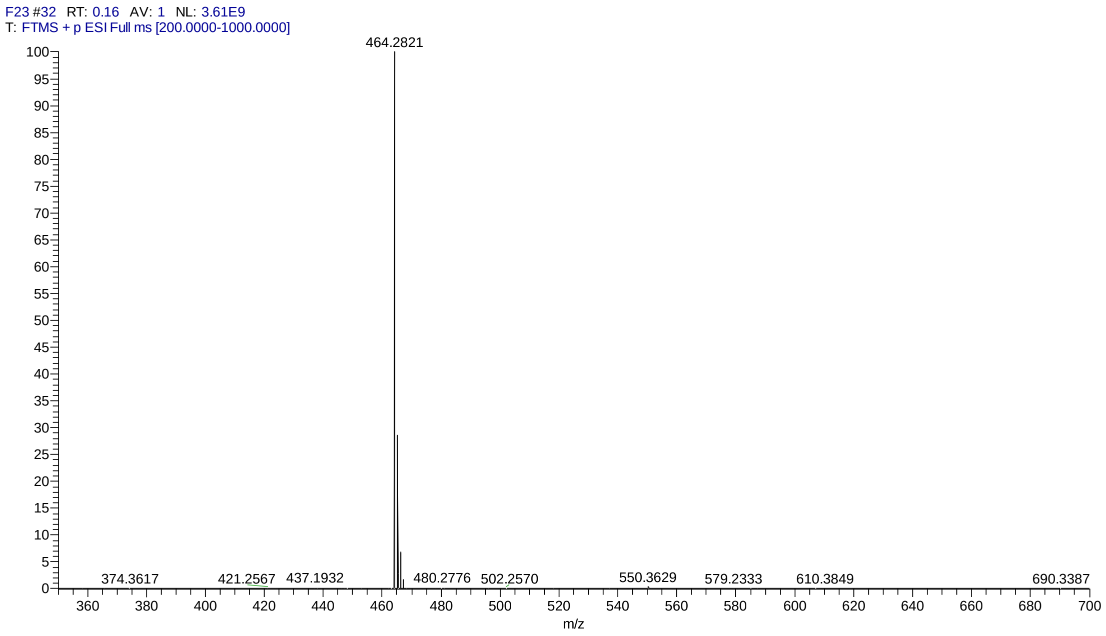

Figure SI 23-3. HR Mass spectrum (ESI) of compound **30**.

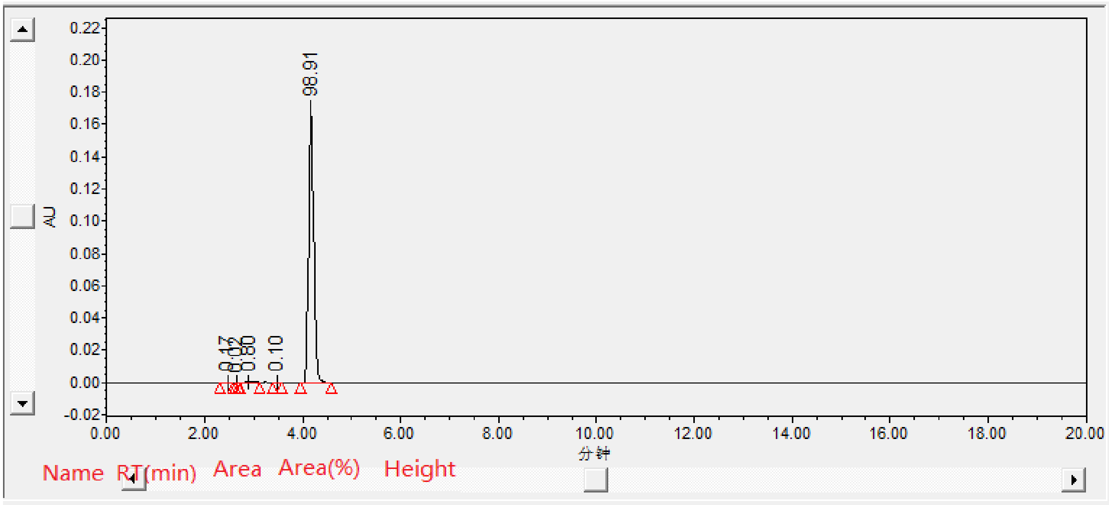

	名称	保留时间 (分钟)	面积 (微伏*秒)	% 面积	高度 (微伏)
1		2.460	2140	0.17	263
2		2.636	248	0.02	83
3		2.896	10216	0.80	769
4		3.466	1342	0.10	230
5		4.167	1267700	98.91	170589

Figure SI 23-4. HPLC of compound **30.**

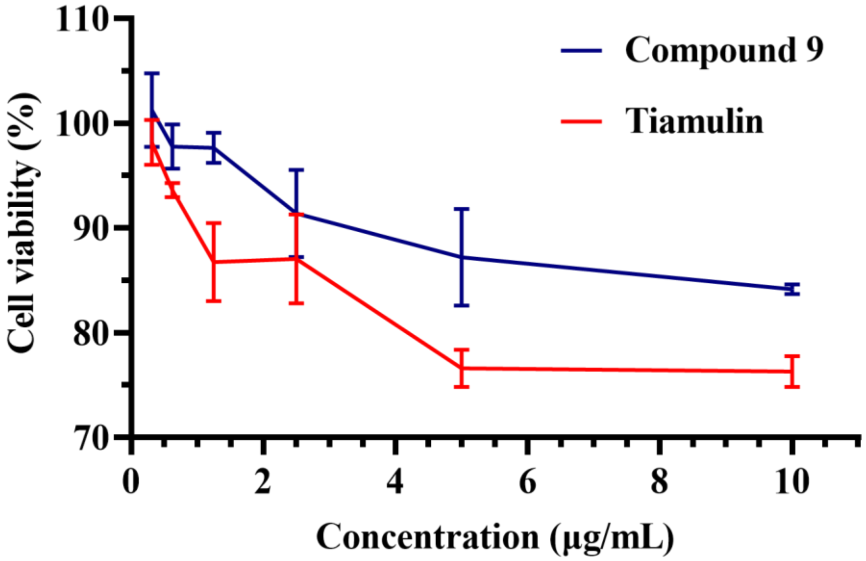

Figure SI 24. The cytotoxicity assay of compound **9** and tiamulin to RAW 264.7 cells.
